# Supplementary material for: Intersection of the Orphan G Protein-Coupled Receptor, GPR19, with the Aging Process
Source: Int J Mol Sci. 2022 Nov 6;23(21):13598. doi: 10.3390/ijms232113598 (PMC9653598; doi:10.3390/ijms232113598)
Supplement: Supplementary file 1 [file ijms-23-13598-s001.zip › Table S1.pdf]

Table S1. Gene Expression Omnibus (GEO) GPR19 associated datasets demonstrating upregulation. The specific GEO datasets (GSE) are indicated and the presence of the associated Gene Symbol for a transcript measured is shown in the different columns.

Gene Symbol	GSE8157	GSE31102	GSE49506	GSE10309	GSE12881	GSE31812	GSE9754	Common Sets
gpr19	1	1	1	1	1	1	1	7
fasn	1	1	1	0	1	1	1	6
flcn	1	1	1	0	1	1	1	6
h6pd	1	1	1	1	1	0	1	6
tmtc4	1	1	1	0	1	1	1	6
fkbp11	1	1	1	1	1	0	1	6
acly	1	1	1	0	1	1	1	6
nampt	1	1	1	0	1	1	1	6
tes	1	1	1	0	1	1	1	6
cab39l	1	1	0	1	1	1	1	6
strbp	1	1	1	0	1	1	1	6
dixdc1	1	0	1	1	1	1	1	6
ddx21	1	1	1	0	1	1	1	6
rif1	1	1	1	0	1	0	1	5
tfrc	1	1	1	0	1	0	1	5
oxr1	1	1	1	0	1	0	1	5
n4bp2l1	1	1	1	0	1	0	1	5
ncapg	1	1	1	0	1	1	0	5
pawr	1	1	1	0	1	1	0	5
ebp	1	1	1	0	0	1	1	5
glrb	1	1	1	0	1	1	0	5
ndufaf4	1	1	1	0	1	0	1	5
acsl4	1	1	1	0	1	1	0	5
slc35b4	1	1	1	0	1	1	0	5
ica1	1	1	0	0	1	1	1	5
proser2	1	0	1	1	1	0	1	5
sox4	1	1	0	0	1	1	1	5
wdr12	1	1	1	0	0	1	1	5
hnrnpd	1	1	1	0	0	1	1	5
sesn3	1	1	0	0	1	1	1	5
nav1	1	0	1	1	1	0	1	5
mcm10	1	1	1	0	0	1	1	5
ccnd2	1	1	1	0	1	0	1	5
tfpi	1	0	1	1	1	1	0	5
gar1	1	1	1	0	1	0	1	5
mtap	1	1	1	0	1	0	1	5
nasp	1	1	1	0	1	1	0	5
dhfr	1	1	0	0	1	1	1	5
serp1	1	1	1	0	0	1	1	5
xbp1	1	1	1	0	1	1	0	5

gnl3	1	1	1	0	0	1	1	5
sept6	1	1	1	0	1	1	0	5
nusap1	1	1	1	0	0	1	1	5
nop58	1	1	0	0	1	1	1	5
rilpl1	1	1	1	0	0	1	1	5
sqle	1	1	1	0	0	1	1	5
cbx5	1	1	1	1	0	0	1	5
wdr4	1	1	1	0	0	1	1	5
fktn	1	1	0	0	1	1	1	5
zbtb20	1	1	1	0	1	0	1	5
aldh1l2	1	0	1	0	1	1	1	5
srr	1	1	1	0	1	0	1	5
dpyd	1	1	1	0	1	1	0	5
skp2	1	1	0	0	1	1	1	5
ldlr	1	1	1	0	1	1	0	5
hk2	1	0	1	0	1	1	1	5
prmt1	1	1	1	0	1	0	1	5
larp6	1	0	0	1	1	1	1	5
nfib	1	1	0	0	1	1	1	5
slc30a4	1	1	0	0	1	1	1	5
cttn	1	1	0	1	1	0	1	5
thbs1	1	0	1	1	1	1	0	5
mapre2	1	1	0	1	1	0	1	5
pdia6	1	1	1	0	1	0	1	5
coa7	1	1	1	0	1	0	1	5
myc	1	0	1	0	1	1	1	5
fkbp4	1	0	1	0	1	1	1	5
sars	1	1	1	0	1	1	0	5
gpsm2	1	0	1	0	1	1	1	5
stmn1	1	1	0	0	1	1	1	5
svil	1	1	1	0	0	1	1	5
farsb	1	1	1	0	0	1	1	5
idi1	1	1	0	0	1	1	1	5
hnrnpab	1	1	1	0	1	0	1	5
pmp22	1	1	0	0	1	1	1	5
syt11	1	1	1	0	1	1	0	5
cad	1	1	1	0	1	0	1	5
syncrip	1	1	1	0	1	0	1	5
vars	1	1	0	0	1	1	1	5
ctsb	1	1	1	1	1	0	0	5
crebrf	1	1	1	0	0	1	1	5
stc2	1	1	0	1	1	1	0	5
vdr	1	0	1	0	1	1	1	5
chpt1	1	1	1	0	1	1	0	5
rabgap1	1	1	0	0	1	1	1	5

ddx54	1	1	1	0	1	0	1	5
stc1	1	1	1	0	0	1	1	5
maoa	1	1	1	0	1	1	0	5
hsph1	1	1	1	0	0	1	1	5
tpd52	1	1	1	0	1	0	1	5
eif3b	1	1	1	0	1	0	1	5
bclaf1	1	1	0	0	1	1	1	5
pes1	1	1	1	0	1	0	1	5
cct5	1	1	1	0	1	0	1	5
higd1a	1	1	1	0	1	0	1	5
mkks	1	1	0	0	1	1	1	5
cyb5b	1	1	1	0	1	0	1	5
dhcr24	1	1	0	0	1	1	1	5
dlgap5	1	1	1	0	0	1	1	5
txnl4a	1	1	1	0	1	0	1	5
abhd17c	1	1	1	0	1	0	1	5
socs2	1	1	1	0	1	0	1	5
pnp	1	1	1	0	1	1	0	5
wars	1	1	1	0	0	1	1	5
sfr1	1	1	1	1	0	1	0	5
slc1a3	1	0	1	0	1	1	1	5
upp1	1	1	0	1	0	1	1	5
kpna2	1	1	0	0	1	1	1	5
cdk1	1	1	0	0	1	1	1	5
cstf3	1	1	0	0	1	1	1	5
bves	1	1	1	0	0	1	1	5
mcl1	1	1	1	0	0	1	1	5
agap1	1	1	1	0	1	0	1	5
adat2	1	0	1	0	1	1	1	5
atf3	1	1	0	0	1	1	1	5
tbc1d15	1	1	0	0	1	1	1	5
mydgf	1	1	1	0	1	0	1	5
aspm	1	1	1	0	0	1	1	5
mina	1	1	1	0	0	1	1	5
cycs	1	1	1	0	1	0	1	5
rnf38	1	1	1	0	1	1	0	5
tspan13	0	1	1	1	1	1	0	5
hspa9	0	1	1	0	1	1	1	5
itga6	0	1	1	0	1	1	1	5
cdkn1a	0	1	1	1	1	1	0	5
srxn1	0	1	1	0	1	1	1	5
fam69b	1	1	0	0	1	0	1	4
nudt13	1	1	1	0	1	0	0	4
tpcn1	1	1	1	0	0	0	1	4
diexf	1	0	1	0	0	1	1	4

polr3g	1	1	1	0	1	0	0	4
jund	1	1	1	0	1	0	0	4
tpm4	1	1	0	0	1	1	0	4
tmem134	1	1	1	0	1	0	0	4
stard10	1	1	1	0	1	0	0	4
abcb6	1	1	0	0	0	1	1	4
rasa3	1	1	0	0	1	1	0	4
dbp	1	1	1	0	0	0	1	4
cask	1	1	0	0	1	1	0	4
fam20c	1	1	0	0	1	0	1	4
gsn	1	0	1	0	1	0	1	4
slc9a3r1	1	1	0	0	1	0	1	4
thra	1	1	0	0	1	0	1	4
fam132a	1	1	0	0	1	0	1	4
anpep	1	0	0	0	1	1	1	4
hipk2	1	1	1	0	1	0	0	4
letm1	1	1	1	0	1	0	0	4
abca7	1	1	1	0	1	0	0	4
st3gal3	1	1	0	0	1	1	0	4
cbfa2t3	1	1	0	0	1	0	1	4
arhgdia	1	1	1	0	0	0	1	4
f11r	1	1	1	0	1	0	0	4
sorbs1	1	1	0	0	1	1	0	4
arl4c	1	1	1	0	0	0	1	4
tmem19	1	1	1	0	0	1	0	4
cdc34	1	1	0	0	1	0	1	4
gatad2a	1	1	1	0	0	0	1	4
dhodh	1	1	1	0	0	0	1	4
gale	1	1	1	0	0	0	1	4
fzd5	1	1	0	0	1	1	0	4
plpp2	1	1	0	0	1	0	1	4
inf2	1	0	1	0	1	1	0	4
fblim1	1	1	0	1	1	0	0	4
six1	1	1	0	0	1	0	1	4
sqstm1	1	1	1	0	0	1	0	4
ikbke	1	1	0	0	0	1	1	4
scara3	1	0	0	0	1	1	1	4
ptpn2	1	1	0	0	1	0	1	4
rdh10	1	1	0	0	1	0	1	4
myo19	1	1	1	0	0	0	1	4
enah	1	1	1	0	0	0	1	4
tgfb2	1	0	0	0	1	1	1	4
wdr1	1	1	0	0	1	0	1	4
kctd10	1	1	0	1	0	1	0	4
tob2	1	1	1	0	1	0	0	4

lpcat3	1	1	1	0	0	1	0	4
pafah1b3	1	0	0	0	1	1	1	4
ppp2r1b	1	1	0	0	1	1	0	4
cenpf	1	1	1	0	0	1	0	4
cd44	1	1	1	0	0	0	1	4
specc1	1	1	1	0	1	0	0	4
hspd1	1	1	1	0	0	0	1	4
nolc1	1	1	1	0	0	0	1	4
iah1	1	0	1	0	1	0	1	4
adgrg1	1	0	1	0	0	1	1	4
exosc1	1	1	1	0	0	0	1	4
dlg3	1	1	0	0	1	0	1	4
egln1	1	1	0	0	1	0	1	4
odf2	1	1	0	0	1	0	1	4
sidt2	1	1	1	0	1	0	0	4
ebf1	1	1	0	0	1	0	1	4
rrp12	1	0	1	0	0	1	1	4
etf1	1	1	1	0	0	0	1	4
ehd4	1	1	1	0	0	0	1	4
mgst1	1	1	1	0	0	0	1	4
lpxn	1	0	1	0	1	1	0	4
pus1	1	1	1	0	0	0	1	4
rab27a	1	1	0	0	1	1	0	4
hbp1	1	0	1	0	1	0	1	4
ppm1l	1	0	1	0	1	1	0	4
utp14a	1	0	1	0	1	0	1	4
klf4	1	1	0	1	0	1	0	4
chka	1	1	0	0	1	0	1	4
mkrn2	1	1	0	0	1	1	0	4
sap30	1	1	0	0	1	0	1	4
plk3	1	1	1	0	0	0	1	4
psmc3ip	1	1	0	0	1	0	1	4
coch	1	1	1	0	0	1	0	4
gosr2	1	1	0	0	0	1	1	4
rbm14	1	1	0	0	0	1	1	4
cmbl	1	0	1	0	1	0	1	4
kif2c	1	1	0	0	0	1	1	4
wdr74	1	0	1	0	0	1	1	4
yipf2	1	1	0	0	1	0	1	4
ppargc1b	1	1	1	0	0	0	1	4
nefl	1	1	0	1	0	0	1	4
tra2a	1	1	0	0	1	0	1	4
stx3	1	1	0	0	1	0	1	4
trim2	1	1	1	0	1	0	0	4
atxn7l3b	1	1	0	1	1	0	0	4

ube2g2	1	1	1	0	0	0	1	4
slc24a3	1	0	1	0	1	1	0	4
brd8	1	1	1	0	0	0	1	4
tspan15	1	1	0	0	0	1	1	4
dnajc10	1	1	1	0	0	1	0	4
hpcal1	1	1	1	0	0	0	1	4
mcfd2	1	1	0	1	0	0	1	4
jak1	1	0	1	0	1	1	0	4
idh1	1	1	0	0	1	0	1	4
hip1	1	0	1	0	1	1	0	4
ap1s1	1	1	0	1	0	0	1	4
celf4	1	1	0	0	1	0	1	4
cib2	1	1	0	0	1	0	1	4
slc7a6	1	1	1	0	0	0	1	4
crtap	1	1	1	0	0	0	1	4
hsp90b1	1	1	1	0	0	0	1	4
tmem33	1	1	1	0	0	0	1	4
tgfb3	1	1	0	0	1	0	1	4
acy3	1	1	0	0	1	1	0	4
nrp2	1	1	0	1	0	0	1	4
pla2g2d	1	1	0	0	1	0	1	4
pfkfb4	1	0	0	0	1	1	1	4
akap9	1	1	1	0	1	0	0	4
polr3d	1	1	0	0	0	1	1	4
polr3h	1	1	1	0	0	0	1	4
atp6v1c2	1	0	0	0	1	1	1	4
glmp	1	1	1	0	0	1	0	4
dnajc18	1	1	0	0	1	0	1	4
pdzrn3	1	1	0	0	1	0	1	4
apaf1	1	0	1	0	0	1	1	4
srsf7	1	1	1	0	1	0	0	4
nop16	1	0	1	0	0	1	1	4
manf	1	1	1	0	0	0	1	4
ppp2cb	1	1	0	0	1	0	1	4
shmt1	1	1	0	0	1	0	1	4
perp	1	1	1	0	1	0	0	4
smim8	1	1	0	0	1	0	1	4
nfkbiz	1	1	0	0	0	1	1	4
pa2g4	1	1	1	0	1	0	0	4
tfdp1	1	1	0	1	0	0	1	4
pum3	1	0	1	0	0	1	1	4
ralgps2	1	1	1	0	0	0	1	4
glcci1	1	1	1	0	1	0	0	4
arpc5	1	1	0	0	1	0	1	4
rcc1	1	0	1	0	1	0	1	4

tra2b	1	1	0	0	1	0	1	4
srsf6	1	1	1	0	1	0	0	4
itm2c	1	0	0	0	1	1	1	4
st14	1	1	0	0	1	1	0	4
wwc1	1	1	1	0	1	0	0	4
gpd2	1	0	0	0	1	1	1	4
chaf1b	1	1	0	0	1	0	1	4
znrf2	1	1	1	0	0	0	1	4
tbrg1	1	1	0	0	1	1	0	4
abhd6	1	1	1	0	1	0	0	4
ikzf4	1	1	1	0	1	0	0	4
eif4ebp2	1	1	0	0	0	1	1	4
spata5	1	1	1	0	0	1	0	4
gpd1	1	1	0	0	1	0	1	4
rhoc	1	1	1	0	0	0	1	4
srsf3	1	1	0	0	1	0	1	4
plp2	1	1	0	0	1	1	0	4
alcam	1	1	0	0	1	0	1	4
zfand3	1	1	0	0	1	1	0	4
fignl1	1	1	1	0	0	0	1	4
iqgap1	1	1	0	0	1	0	1	4
chd1	1	1	0	0	1	1	0	4
usp3	1	1	1	0	1	0	0	4
pip4k2a	1	0	1	0	1	1	0	4
tpp1	1	1	1	0	0	0	1	4
tmem120b	1	1	1	0	1	0	0	4
ska2	1	1	0	0	0	1	1	4
clasp2	1	1	0	0	1	1	0	4
cdkn2b	1	1	1	0	1	0	0	4
tial1	1	1	1	0	0	0	1	4
emp2	1	1	0	0	0	1	1	4
fbn2	1	1	0	1	1	0	0	4
rbp1	1	1	0	0	1	0	1	4
cmas	1	1	0	0	1	0	1	4
arhgap9	1	1	1	0	1	0	0	4
serbp1	1	1	1	0	0	0	1	4
btg2	1	1	1	0	0	0	1	4
lpcat2	1	0	0	0	1	1	1	4
usp2	1	0	1	0	1	0	1	4
tspan31	1	1	1	1	0	0	0	4
ptprf	1	1	1	0	1	0	0	4
cfb	1	1	0	0	0	1	1	4
cmtm7	1	1	1	1	0	0	0	4
mgp	1	0	0	0	1	1	1	4
triobp	1	1	0	0	1	0	1	4

hbegf	1	1	0	0	0	1	1	4
lamb3	1	0	0	0	1	1	1	4
use1	1	1	0	0	0	1	1	4
trib3	1	1	1	0	0	1	0	4
phlda1	1	1	0	0	1	0	1	4
snx5	1	1	1	0	0	0	1	4
srprb	1	1	1	0	0	0	1	4
klhl13	1	1	1	0	0	0	1	4
pxk	1	1	0	0	1	1	0	4
grina	1	1	0	0	1	0	1	4
bcl2l13	1	0	0	0	1	1	1	4
rabgap1l	1	1	0	0	1	0	1	4
mdm1	1	1	1	0	0	0	1	4
rhpn2	1	1	0	0	1	0	1	4
cpeb2	1	0	1	0	1	1	0	4
haus6	1	0	1	0	1	0	1	4
bicd1	1	1	1	0	0	0	1	4
orc1	1	1	0	0	0	1	1	4
cadm1	1	1	1	0	1	0	0	4
sord	1	1	1	0	0	0	1	4
polr2f	1	1	1	0	0	0	1	4
antxr1	1	0	0	1	1	1	0	4
kpnb1	1	1	1	0	0	0	1	4
bccip	1	1	1	0	0	0	1	4
uhrf1bp1l	1	1	0	0	1	0	1	4
tubb2b	1	1	0	0	1	0	1	4
cnot6l	1	1	0	0	0	1	1	4
gtse1	1	1	0	0	0	1	1	4
mfsd6	1	1	0	0	1	0	1	4
nol4l	1	0	1	0	1	0	1	4
cryzl1	1	1	1	1	0	0	0	4
brca1	1	1	0	0	1	0	1	4
npas3	1	1	1	0	1	0	0	4
timm44	1	1	1	0	0	0	1	4
agfg2	1	1	1	0	1	0	0	4
nek2	1	1	1	0	0	0	1	4
dgat2	1	1	0	0	1	0	1	4
insig1	1	0	1	0	1	1	0	4
gucy1b3	1	1	0	1	0	1	0	4
srsf1	1	1	1	0	0	0	1	4
abhd2	1	1	1	0	0	0	1	4
ctbp2	1	1	0	0	1	0	1	4
trmt1	1	1	1	0	0	1	0	4
il17ra	1	1	1	0	0	0	1	4
fbxo2	1	1	0	0	0	1	1	4

gltp	1	1	0	0	1	1	0	4
hnrnpu	1	1	1	0	0	0	1	4
man2c1	1	1	1	0	0	0	1	4
cdc25c	1	0	1	0	0	1	1	4
plscr1	1	0	0	0	1	1	1	4
tjp2	1	0	0	1	1	0	1	4
gata3	1	0	0	0	1	1	1	4
chac2	1	1	1	0	1	0	0	4
lpp	1	1	0	1	1	0	0	4
rab3ip	1	1	1	0	0	0	1	4
rerg	1	0	0	0	1	1	1	4
ftsj3	1	1	1	0	0	0	1	4
ssbp3	1	1	1	0	0	1	0	4
b4galt3	1	1	1	0	1	0	0	4
qtrt1	1	0	0	0	1	1	1	4
utrn	1	1	1	0	1	0	0	4
hectd1	1	1	0	0	1	1	0	4
sec14l1	1	1	1	0	0	0	1	4
rhob	1	1	1	0	0	0	1	4
id2	1	0	1	0	1	1	0	4
caprin1	1	1	0	0	1	0	1	4
ctsa	1	1	0	0	0	1	1	4
csnk2a2	1	1	0	0	1	1	0	4
myo5b	1	0	1	0	1	1	0	4
noc2l	1	0	1	0	1	1	0	4
s100a1	1	1	0	0	1	0	1	4
usp6nl	1	0	1	0	1	0	1	4
rpe	1	1	0	0	0	1	1	4
orc2	1	0	0	0	1	1	1	4
ncl	1	1	0	0	1	0	1	4
sec63	1	1	1	0	0	1	0	4
hlx	1	1	0	0	1	0	1	4
tnfrsf21	1	1	0	1	1	0	0	4
slco3a1	1	0	1	0	1	0	1	4
naa25	1	0	1	0	0	1	1	4
hnrnpa2b1	1	1	0	0	1	0	1	4
mcm5	1	0	0	0	1	1	1	4
tspan6	1	1	0	0	0	1	1	4
eif5a	1	1	1	0	0	0	1	4
mrps25	1	1	1	0	0	0	1	4
fdft1	1	1	1	0	1	0	0	4
prelid1	1	1	0	0	1	0	1	4
fitm2	1	0	1	0	1	0	1	4
arih2	1	1	1	0	0	1	0	4
ankef1	1	0	1	1	1	0	0	4

pwp1	1	0	1	0	1	0	1	4
frmd4b	1	1	0	0	1	1	0	4
eif4g1	1	1	0	0	1	0	1	4
mis18bp1	1	0	1	0	1	0	1	4
ddr2	1	0	0	0	1	1	1	4
tufm	1	1	1	0	0	0	1	4
frk	1	1	0	0	1	1	0	4
spata6	1	1	0	1	0	0	1	4
malat1	1	1	1	0	1	0	0	4
ptprk	1	1	0	1	1	0	0	4
ywhag	1	0	1	0	1	0	1	4
eif2s2	1	1	1	0	1	0	0	4
phgdh	1	1	0	0	1	0	1	4
bhlha15	1	0	1	0	1	0	1	4
metap2	1	1	1	0	0	0	1	4
fyn	1	0	1	0	1	1	0	4
stau2	1	1	0	0	1	1	0	4
slc25a51	1	1	0	0	1	1	0	4
tmpo	1	1	0	0	1	0	1	4
slc39a6	1	1	1	0	0	0	1	4
plscr2	1	0	0	0	1	1	1	4
msn	1	1	0	0	0	1	1	4
exph5	1	0	1	0	1	1	0	4
sfxn4	1	0	1	0	1	0	1	4
tcn2	1	1	0	0	0	1	1	4
smim10l1	1	1	0	1	1	0	0	4
rell1	1	1	0	0	1	0	1	4
prdm1	1	0	1	0	1	1	0	4
tsc22d1	1	1	1	0	0	0	1	4
hnrnpdl	1	1	1	0	1	0	0	4
snai2	1	1	1	0	0	0	1	4
col17a1	1	0	0	1	1	0	1	4
ppib	1	1	1	0	1	0	0	4
racgap1	1	1	0	0	0	1	1	4
adcy9	1	1	1	0	0	0	1	4
parp9	1	1	1	0	1	0	0	4
atad2b	1	1	1	0	1	0	0	4
wdr55	1	1	0	0	1	0	1	4
kctd5	1	1	1	0	0	0	1	4
tmem106a	1	1	1	0	1	0	0	4
dhx9	1	0	1	0	0	1	1	4
snapc3	1	1	0	0	1	0	1	4
tm9sf3	1	1	1	0	0	0	1	4
tkt	1	1	1	0	1	0	0	4
clip4	1	1	0	0	1	1	0	4

pcmtd1	1	0	1	0	1	0	1	4
cd9	1	0	1	0	1	1	0	4
tap2	1	1	1	0	1	0	0	4
brd3	1	1	1	0	1	0	0	4
gdpd3	1	1	0	0	1	1	0	4
aimp2	1	1	0	0	1	0	1	4
iffo1	1	1	0	0	1	0	1	4
taf9b	1	0	1	0	0	1	1	4
zmpste24	1	1	1	0	0	0	1	4
prkd1	1	1	0	0	1	0	1	4
heatr1	1	0	1	0	0	1	1	4
dhcr7	1	1	1	0	0	1	0	4
sft2d2	1	1	0	0	1	1	0	4
ckb	1	0	1	0	1	1	0	4
nudcd1	1	0	1	0	1	0	1	4
mecom	1	0	1	0	1	0	1	4
ppif	1	0	1	0	1	0	1	4
dyrk1a	1	1	1	0	0	0	1	4
polr2h	1	1	1	0	0	0	1	4
arl6ip5	1	0	0	0	1	1	1	4
zmym1	1	0	1	0	1	0	1	4
adm	1	0	1	1	0	0	1	4
papola	1	1	1	0	0	0	1	4
mllt3	1	1	0	0	1	0	1	4
b4galt6	1	1	0	0	0	1	1	4
sdhd	1	1	0	0	1	0	1	4
pnpt1	1	0	1	0	0	1	1	4
hey1	1	0	1	0	1	0	1	4
mylk	1	1	0	0	1	0	1	4
srsf10	1	1	1	0	0	0	1	4
ezr	1	1	1	0	0	0	1	4
pdcd11	1	0	1	0	0	1	1	4
emc6	1	1	0	0	1	0	1	4
ltbp3	1	1	0	1	0	0	1	4
csnk2a1	1	1	1	0	1	0	0	4
pard3	1	1	0	0	1	1	0	4
tax1bp1	1	1	0	0	1	1	0	4
kpna3	1	1	0	0	1	0	1	4
txn2	1	1	0	0	1	0	1	4
tab2	1	0	1	0	1	1	0	4
lypd6	1	0	0	1	1	0	1	4
prps2	1	0	1	0	1	0	1	4
elf2	1	0	1	0	1	0	1	4
pde8a	1	1	0	0	1	0	1	4
cdca5	1	1	0	0	1	0	1	4

cttnbp2nl	1	0	1	0	1	0	1	4
far1	1	1	1	0	0	0	1	4
tcof1	1	0	1	0	0	1	1	4
ece2	1	0	1	0	1	0	1	4
errfi1	1	1	0	0	0	1	1	4
suox	1	0	1	0	0	1	1	4
dera	1	1	0	0	1	0	1	4
mylip	1	1	1	0	0	0	1	4
dctpp1	1	0	1	0	1	0	1	4
hspa5	1	1	1	0	0	1	0	4
hexa	1	1	1	0	0	0	1	4
gabarap	1	1	1	0	0	0	1	4
rprd1a	1	1	1	0	1	0	0	4
rnf145	1	1	0	0	1	0	1	4
ift81	1	1	1	0	0	0	1	4
sdcbp	1	1	1	0	0	0	1	4
sugt1	1	1	1	1	0	0	0	4
bcl6	1	1	1	0	1	0	0	4
rbm17	1	1	0	1	0	0	1	4
tmed5	1	1	1	0	0	0	1	4
taf7	1	1	0	0	1	1	0	4
pole2	1	1	0	0	0	1	1	4
dusp6	1	0	0	1	1	0	1	4
fkbp1b	1	1	1	0	0	0	1	4
arl6ip1	1	1	1	0	0	0	1	4
ak2	1	1	1	0	1	0	0	4
fez1	1	1	1	0	1	0	0	4
mybl2	1	1	0	0	0	1	1	4
sel1l	1	1	1	0	0	1	0	4
anapc5	1	0	1	0	1	0	1	4
epb41l5	1	0	1	0	1	1	0	4
bub1	1	1	1	0	0	0	1	4
tmed3	1	1	1	0	0	0	1	4
cybrd1	1	0	1	1	1	0	0	4
nus1	1	1	1	0	0	0	1	4
nrm	1	1	0	0	0	1	1	4
hnrnpll	1	1	0	0	1	0	1	4
stat3	1	0	1	0	1	1	0	4
samd9l	1	0	1	0	1	0	1	4
cct2	1	1	1	0	1	0	0	4
il1r1	1	1	0	0	1	0	1	4
khdrbs3	1	1	0	0	1	0	1	4
glul	1	1	0	0	1	0	1	4
bcl2	1	0	0	0	1	1	1	4
p2rx7	1	0	0	0	1	1	1	4

calml4	1	1	0	0	1	0	1	4
map2k5	1	1	1	0	0	1	0	4
slc44a1	1	1	1	0	1	0	0	4
arhgap24	1	1	0	0	1	0	1	4
atf7ip	1	1	1	0	1	0	0	4
trps1	1	1	1	0	1	0	0	4
phip	1	1	1	0	1	0	0	4
rragc	1	1	0	0	1	1	0	4
cyp39a1	1	1	0	0	0	1	1	4
tmem144	1	1	0	0	1	0	1	4
diaph3	1	1	0	0	0	1	1	4
aurka	1	1	0	0	0	1	1	4
morf4l2	1	1	0	0	1	0	1	4
hspa4l	1	1	1	0	0	0	1	4
epb41l4a	1	0	1	0	1	0	1	4
ndufa9	1	1	0	0	1	0	1	4
junb	1	1	0	0	0	1	1	4
sort1	1	1	0	0	1	0	1	4
msmo1	1	1	0	0	1	0	1	4
c1qbp	1	1	1	0	0	0	1	4
nedd9	1	1	0	0	0	1	1	4
fmo1	1	1	0	0	1	0	1	4
clic5	1	0	1	0	1	1	0	4
zeb1	1	1	0	0	1	0	1	4
alk	1	1	0	0	1	0	1	4
plpp3	1	1	1	0	0	0	1	4
pigp	1	1	0	0	1	0	1	4
aasdhppt	1	0	1	0	1	0	1	4
tspan7	1	1	0	0	1	0	1	4
plekha7	1	1	1	0	1	0	0	4
knop1	1	1	1	0	1	0	0	4
flad1	1	1	1	0	1	0	0	4
mrpl35	1	1	1	0	0	0	1	4
snd1	1	1	0	0	1	0	1	4
pgam1	1	1	0	0	1	0	1	4
fam162a	1	1	1	0	0	0	1	4
ppp1r15a	1	1	0	0	0	1	1	4
rpia	1	1	1	0	0	0	1	4
amd1	1	0	1	0	1	0	1	4
mcm4	1	1	0	0	1	0	1	4
kif1b	1	1	1	0	1	0	0	4
nrp1	1	1	1	0	0	1	0	4
cnpy2	1	1	1	0	1	0	0	4
wdr43	1	1	1	0	0	1	0	4
pfkp	1	1	1	0	1	0	0	4

ywhaz	1	1	0	0	1	0	1	4
wdr36	1	0	1	0	0	1	1	4
klhl7	1	1	1	0	1	0	0	4
uchl5	1	1	0	0	0	1	1	4
opa1	1	1	0	0	1	0	1	4
polr3k	1	0	1	0	1	0	1	4
nqo2	1	1	0	0	1	0	1	4
ino80c	1	1	1	0	0	0	1	4
set	1	1	0	0	1	0	1	4
mns1	1	0	0	0	1	1	1	4
bid	1	1	1	0	0	0	1	4
mtdh	1	1	1	0	0	1	0	4
klf6	1	1	0	0	0	1	1	4
prcp	1	1	0	0	1	1	0	4
uchl1	1	1	0	0	0	1	1	4
csnk1d	1	1	0	0	1	0	1	4
oard1	1	1	0	0	0	1	1	4
hmox1	1	1	0	1	0	0	1	4
rrp9	1	0	1	0	0	1	1	4
zfyve1	1	0	1	0	1	0	1	4
mras	1	1	0	0	1	1	0	4
aida	1	1	0	0	1	0	1	4
zmynd19	1	0	1	0	1	0	1	4
csgalnact2	1	1	0	0	1	1	0	4
ucp2	1	0	1	0	1	1	0	4
palld	1	1	0	0	1	1	0	4
isg20	1	1	1	1	0	0	0	4
ybx3	1	1	1	0	0	0	1	4
trim27	1	1	1	0	1	0	0	4
picalm	1	1	1	0	1	0	0	4
rps24	1	1	1	0	0	0	1	4
col8a1	1	1	0	0	1	0	1	4
nhp2	1	1	0	0	1	0	1	4
ppp2r5b	1	0	0	0	1	1	1	4
sfpq	1	1	0	0	0	1	1	4
pqlc3	1	1	0	0	1	0	1	4
slc52a2	1	1	1	0	1	0	0	4
itgb1bp1	1	1	1	0	1	0	0	4
ncapd2	1	0	0	0	1	1	1	4
inafm2	1	1	1	0	1	0	0	4
tmem168	1	1	1	0	0	1	0	4
gpatch4	1	1	1	0	0	0	1	4
cited2	1	1	1	0	0	1	0	4
wdr5	1	1	1	0	0	0	1	4
jun	1	1	1	0	1	0	0	4

aldh6a1	1	0	1	0	1	0	1	4
pask	1	0	0	0	1	1	1	4
wdr3	1	0	1	0	0	1	1	4
fam13b	1	1	1	0	1	0	0	4
spdl1	1	1	0	0	1	0	1	4
serping1	1	0	0	0	1	1	1	4
tmem106b	1	1	0	1	1	0	0	4
cox5b	1	1	0	0	1	0	1	4
jmy	1	1	0	0	1	1	0	4
psma5	1	1	1	0	0	0	1	4
gtf3a	1	1	1	0	0	0	1	4
carhsp1	1	1	0	0	0	1	1	4
armcx3	1	1	1	0	1	0	0	4
rpp40	1	0	1	0	0	1	1	4
pbx1	1	0	1	0	1	1	0	4
dtl	1	1	0	0	0	1	1	4
eif1ax	1	1	1	0	0	0	1	4
ahsa1	1	1	1	0	0	0	1	4
tomm20	1	1	1	0	0	0	1	4
psme3	1	1	1	0	0	0	1	4
pex7	1	1	0	0	1	0	1	4
fam213a	1	0	0	0	1	1	1	4
hspe1	1	1	1	0	0	0	1	4
rftn1	1	0	1	0	1	0	1	4
zwint	0	1	0	0	1	1	1	4
il2rg	0	1	0	0	1	1	1	4
nars	0	1	0	0	1	1	1	4
cebpb	0	1	1	1	0	0	1	4
mcm2	0	1	0	0	1	1	1	4
emb	0	1	0	1	1	0	1	4
slc7a5	0	1	1	0	1	1	0	4
timp2	0	1	1	0	1	0	1	4
pttg1	0	1	0	0	1	1	1	4
reep5	0	1	0	0	1	1	1	4
mcm6	0	1	0	0	1	1	1	4
trim37	0	1	0	0	1	1	1	4
tmem97	0	1	1	0	0	1	1	4
syne2	0	1	1	0	1	0	1	4
pmm2	0	1	1	0	0	1	1	4
ldha	0	1	1	0	0	1	1	4
elovl1	0	1	0	0	1	1	1	4
acat2	0	1	0	0	1	1	1	4
hs6st2	0	1	0	0	1	1	1	4
tpx2	0	1	0	0	1	1	1	4
ccnb2	0	1	1	0	0	1	1	4

nap1l1	0	1	1	0	1	0	1	4
utp6	0	1	1	0	0	1	1	4
hsp90ab1	0	1	1	0	1	0	1	4
fam129a	0	1	1	0	0	1	1	4
noc3l	0	1	1	0	0	1	1	4
nrep	0	1	0	1	1	1	0	4
rfk	0	1	1	0	1	0	1	4
pstpip2	0	1	0	0	1	1	1	4
nme7	0	1	1	0	1	0	1	4
trpt1	0	1	1	0	1	0	1	4
anxa7	0	1	1	0	1	0	1	4
top2a	0	1	1	0	0	1	1	4
mybbp1a	0	1	1	0	1	0	1	4
cdkn2c	0	1	1	0	1	1	0	4
pcolce2	0	1	1	0	1	0	1	4
zpr1	0	1	1	0	0	1	1	4
cdc37l1	0	1	1	0	1	1	0	4
maged2	0	1	1	0	0	1	1	4
slc12a2	0	1	1	0	1	1	0	4
cd55	0	1	1	0	1	1	0	4
cdca7	0	1	0	0	1	1	1	4
frat2	0	1	1	1	1	0	0	4
cdc25a	0	1	1	0	1	0	1	4
smpdl3b	0	1	0	0	1	1	1	4
slc25a33	0	1	1	0	1	0	1	4
nudt19	0	1	1	0	1	0	1	4
parp1	0	1	0	0	1	1	1	4
slc39a14	0	1	1	0	1	0	1	4
ect2	0	1	0	0	1	1	1	4
dhrs7	0	1	1	0	1	0	1	4
sh3bp5	0	1	1	0	1	0	1	4
kdelr2	0	1	1	0	1	0	1	4
tjp1	0	1	0	1	1	0	1	4
slc1a5	0	1	1	0	1	0	1	4
nufip1	0	1	1	0	0	1	1	4
dnaja3	0	1	1	0	1	1	0	4
maged1	0	1	0	1	0	1	1	4
appl2	0	1	1	0	1	0	1	4
polr3gl	0	1	1	0	1	0	1	4
fam43a	0	1	1	0	0	1	1	4
rgs2	0	1	1	0	0	1	1	4
rabggtb	0	1	1	0	0	1	1	4
odc1	0	1	1	0	1	0	1	4
egr1	0	0	1	0	1	1	1	4
cth	0	0	1	0	1	1	1	4

pus7	0	0	1	0	1	1	1	4
slc25a32	0	0	1	0	1	1	1	4
arg2	0	0	1	1	1	1	0	4
sqrdl	0	0	0	1	1	1	1	4
ppp1r3c	0	0	0	1	1	1	1	4
exo1	0	0	0	1	1	1	1	4
pcdhb13	1	0	0	0	0	1	1	3
mast4	1	0	0	1	1	0	0	3
gdf15	1	0	0	0	0	1	1	3
mov10	1	0	1	0	0	1	0	3
cdk6	1	0	0	0	1	0	1	3
psmg4	1	1	1	0	0	0	0	3
isyna1	1	1	0	0	0	0	1	3
fam134b	1	1	0	0	1	0	0	3
arhgef37	1	0	1	0	0	1	0	3
slc37a1	1	0	0	0	1	1	0	3
nfkbia	1	0	0	0	1	1	0	3
cdk14	1	1	0	0	0	1	0	3
cd40	1	1	0	0	1	0	0	3
slc19a1	1	0	1	0	0	0	1	3
meltf	1	0	1	0	0	1	0	3
nup210	1	1	0	0	1	0	0	3
tro	1	1	0	0	1	0	0	3
ehd1	1	1	0	0	0	1	0	3
arhgef12	1	0	1	0	1	0	0	3
ap1g2	1	1	0	0	0	0	1	3
calca	1	1	0	0	1	0	0	3
kif22	1	0	0	0	1	0	1	3
dtnb	1	1	0	0	1	0	0	3
hist3h2a	1	1	0	0	0	0	1	3
ssu72	1	1	0	0	0	0	1	3
nin	1	1	0	0	0	0	1	3
slc15a4	1	1	1	0	0	0	0	3
farp1	1	1	0	0	1	0	0	3
rbbp6	1	1	0	0	0	0	1	3
psmd4	1	1	0	0	0	0	1	3
ptprc	1	0	1	0	1	0	0	3
vamp1	1	0	0	0	1	1	0	3
yme1l1	1	1	0	0	0	1	0	3
fam19a5	1	1	0	0	0	0	1	3
chst15	1	0	0	0	1	0	1	3
cep104	1	1	0	0	0	1	0	3
agfg1	1	1	0	0	1	0	0	3
tlcd2	1	1	0	0	1	0	0	3
atf5	1	1	0	0	0	0	1	3

parp3	1	0	0	0	1	0	1	3
fer	1	1	0	0	1	0	0	3
taf6l	1	1	0	0	0	0	1	3
epb41l1	1	1	0	0	1	0	0	3
avil	1	1	1	0	0	0	0	3
grk6	1	0	0	0	1	0	1	3
fam104a	1	1	0	0	0	0	1	3
syt9	1	1	0	0	1	0	0	3
arhgap44	1	0	0	0	1	0	1	3
nt5c	1	1	0	0	1	0	0	3
nr2f1	1	1	1	0	0	0	0	3
arfrp1	1	1	0	0	0	0	1	3
dnajc4	1	0	1	0	1	0	0	3
auts2	1	1	0	0	1	0	0	3
mical2	1	0	0	0	0	1	1	3
os9	1	1	0	0	0	0	1	3
pcp4	1	1	0	0	1	0	0	3
atf2	1	1	0	0	0	0	1	3
trio	1	1	0	0	1	0	0	3
ptpn6	1	1	0	0	1	0	0	3
jag1	1	0	1	0	0	1	0	3
cntrob	1	1	0	0	0	0	1	3
usp37	1	0	0	0	1	0	1	3
stx2	1	0	0	0	1	1	0	3
fam83h	1	0	0	1	1	0	0	3
pdlim5	1	1	0	0	0	1	0	3
irak1	1	1	1	0	0	0	0	3
opa3	1	1	0	0	0	0	1	3
st3gal6	1	0	0	0	1	1	0	3
fbrsl1	1	0	0	0	1	0	1	3
epha1	1	0	0	0	1	0	1	3
usp49	1	0	0	0	1	1	0	3
slc1a1	1	1	0	0	0	1	0	3
spire2	1	1	0	0	0	0	1	3
cadm4	1	0	0	0	0	1	1	3
hs3st3b1	1	0	1	0	1	0	0	3
nsun6	1	1	0	0	0	0	1	3
sptlc2	1	1	0	0	1	0	0	3
fam84a	1	1	0	0	0	1	0	3
gtf2f1	1	1	0	0	0	0	1	3
trafd1	1	0	1	0	0	0	1	3
ap2a2	1	1	0	0	0	0	1	3
rab40b	1	1	0	0	1	0	0	3
aurkb	1	0	0	0	0	1	1	3
tpm3	1	1	0	0	0	0	1	3

parvb	1	0	1	0	1	0	0	3
pld1	1	1	1	0	0	0	0	3
rfx3	1	0	1	0	1	0	0	3
grik5	1	1	0	0	0	0	1	3
lrig1	1	0	0	0	0	1	1	3
pabpc1	1	1	0	0	0	0	1	3
slc27a4	1	1	1	0	0	0	0	3
marveld2	1	1	0	0	1	0	0	3
mafk	1	1	1	0	0	0	0	3
ankrd44	1	1	0	0	1	0	0	3
aldh7a1	1	0	0	0	0	1	1	3
pex16	1	1	0	0	1	0	0	3
faf1	1	1	0	0	0	1	0	3
mib1	1	1	0	0	0	0	1	3
wnk1	1	1	0	0	0	0	1	3
aebp1	1	0	0	0	1	0	1	3
ids	1	1	1	0	0	0	0	3
hspa4	1	1	0	0	0	0	1	3
pappa	1	0	0	1	1	0	0	3
sdsl	1	0	1	0	1	0	0	3
scara5	1	1	0	0	1	0	0	3
rbm19	1	1	0	0	0	0	1	3
psen2	1	0	0	0	1	0	1	3
igfbp5	1	0	0	0	0	1	1	3
tfb2m	1	0	1	0	1	0	0	3
pom121	1	1	0	0	0	0	1	3
fubp1	1	1	0	0	0	0	1	3
mvk	1	1	0	0	0	1	0	3
krt19	1	0	0	0	1	0	1	3
arx	1	1	0	0	0	0	1	3
dr1	1	1	0	0	0	0	1	3
stk24	1	1	0	0	0	1	0	3
mpc1	1	1	0	0	1	0	0	3
mbp	1	1	0	0	1	0	0	3
nabp1	1	0	0	0	1	0	1	3
pcm1	1	1	0	0	0	1	0	3
slc7a1	1	0	1	0	1	0	0	3
tnrc6c	1	1	1	0	0	0	0	3
atp7a	1	1	0	0	0	1	0	3
nppb	1	1	0	0	0	0	1	3
fzr1	1	1	0	0	0	0	1	3
nme5	1	0	0	0	1	0	1	3
dennd5b	1	0	1	0	1	0	0	3
por	1	1	0	0	1	0	0	3
cald1	1	1	0	0	0	0	1	3

pyroxd2	1	0	1	0	0	1	0	3
prkd2	1	1	1	0	0	0	0	3
ncf4	1	0	0	0	1	0	1	3
nadk	1	1	0	0	0	0	1	3
ehd3	1	1	0	0	0	0	1	3
ano1	1	0	0	0	1	1	0	3
abtb1	1	1	0	0	0	0	1	3
cdk18	1	0	0	0	1	0	1	3
cbfb	1	1	0	0	0	0	1	3
hgsnat	1	1	1	0	0	0	0	3
fam131a	1	1	0	0	0	0	1	3
ipo4	1	0	0	0	1	1	0	3
msra	1	1	0	0	1	0	0	3
wdr46	1	0	0	0	0	1	1	3
dlg1	1	1	0	0	1	0	0	3
dnajc9	1	0	1	0	0	0	1	3
parva	1	1	0	0	1	0	0	3
bmp2k	1	1	0	0	1	0	0	3
gfer	1	1	0	0	0	0	1	3
eml6	1	1	0	0	0	0	1	3
nos3	1	1	0	0	1	0	0	3
adh7	1	1	0	0	0	0	1	3
pik3c2a	1	1	0	0	1	0	0	3
smc1a	1	1	0	0	0	0	1	3
cchcr1	1	0	1	0	0	0	1	3
tcf3	1	0	1	0	0	0	1	3
pdgfc	1	0	0	0	1	1	0	3
inpp5a	1	1	0	0	1	0	0	3
pvr	1	1	0	0	0	0	1	3
p2rx6	1	0	0	0	1	0	1	3
dnajc21	1	1	0	0	1	0	0	3
trip11	1	1	0	0	1	0	0	3
itgb4	1	1	0	0	1	0	0	3
pck2	1	1	0	0	0	1	0	3
meg3	1	1	0	0	1	0	0	3
cenpi	1	0	0	0	1	1	0	3
cpsf3l	1	1	0	0	0	0	1	3
tmem156	1	0	1	0	1	0	0	3
usp36	1	0	1	0	0	1	0	3
synj2	1	0	1	1	0	0	0	3
igsf3	1	0	0	0	1	0	1	3
il21r	1	0	1	0	1	0	0	3
c3orf58	1	0	1	0	0	1	0	3
prkcb	1	1	1	0	0	0	0	3
mmd2	1	1	0	0	1	0	0	3

ptpn11	1	0	0	0	1	0	1	3
mtf1	1	0	1	0	0	0	1	3
trpv4	1	0	0	0	1	1	0	3
cenpv	1	1	1	0	0	0	0	3
traip	1	0	0	0	1	0	1	3
dusp9	1	1	0	0	0	0	1	3
b3galt2	1	0	1	0	1	0	0	3
il27ra	1	0	0	0	1	1	0	3
pdxk	1	0	0	1	0	0	1	3
slc25a53	1	1	0	0	1	0	0	3
cyhr1	1	1	0	0	0	0	1	3
inhbe	1	0	1	0	0	1	0	3
sec61a1	1	0	0	0	1	0	1	3
cln6	1	0	1	0	0	1	0	3
adi1	1	1	1	0	0	0	0	3
gnptab	1	1	0	0	1	0	0	3
samm50	1	0	0	0	1	0	1	3
mmaa	1	1	0	0	1	0	0	3
mettl9	1	1	1	0	0	0	0	3
tnfrsf1b	1	0	1	0	1	0	0	3
foxs1	1	0	0	0	1	0	1	3
gtf2a1	1	1	1	0	0	0	0	3
acox1	1	1	0	0	1	0	0	3
cdyl	1	1	0	0	0	1	0	3
tlcd1	1	0	0	0	1	0	1	3
lhx9	1	0	1	0	0	0	1	3
cep131	1	1	0	0	1	0	0	3
cntnap2	1	1	0	0	1	0	0	3
jmjd1c	1	1	1	0	0	0	0	3
trim8	1	1	0	0	0	0	1	3
rtkn	1	0	0	0	1	0	1	3
myef2	1	1	0	0	0	0	1	3
gtf2i	1	1	0	0	1	0	0	3
srek1ip1	1	1	1	0	0	0	0	3
slc1a2	1	1	0	0	0	1	0	3
celf2	1	0	1	0	1	0	0	3
rad51d	1	1	0	0	1	0	0	3
chgb	1	1	0	1	0	0	0	3
epb41l4b	1	0	0	0	1	1	0	3
padi2	1	0	0	0	1	1	0	3
gm2a	1	1	0	0	0	1	0	3
fbxw9	1	1	0	0	0	0	1	3
adtrp	1	1	0	0	1	0	0	3
sptbn1	1	1	0	0	0	0	1	3
kank2	1	0	0	0	1	1	0	3

rassf3	1	1	0	0	0	0	1	3
muc4	1	0	0	1	1	0	0	3
ntmt1	1	1	1	0	0	0	0	3
cep290	1	0	0	0	0	1	1	3
erbb3	1	1	0	0	1	0	0	3
plekha8	1	0	1	1	0	0	0	3
itgb2	1	0	0	0	1	1	0	3
nags	1	0	1	1	0	0	0	3
csnk1a1	1	1	1	0	0	0	0	3
prpsap1	1	1	1	0	0	0	0	3
ss18	1	0	1	0	1	0	0	3
kdm5b	1	1	0	0	1	0	0	3
il3ra	1	1	0	0	0	0	1	3
kif5c	1	1	0	0	1	0	0	3
pid1	1	0	0	0	1	0	1	3
hdlbp	1	1	0	0	1	0	0	3
exog	1	0	1	0	1	0	0	3
whsc1	1	0	1	0	1	0	0	3
myo7a	1	1	0	0	1	0	0	3
azin1	1	1	0	0	0	0	1	3
mphosph9	1	1	1	0	0	0	0	3
rcn3	1	1	0	0	0	0	1	3
nup50	1	1	0	0	0	0	1	3
cers4	1	1	0	0	1	0	0	3
dhx30	1	1	0	1	0	0	0	3
ncaph2	1	0	0	0	1	0	1	3
ckap2l	1	0	0	0	0	1	1	3
aprt	1	1	0	0	0	0	1	3
mta1	1	1	1	0	0	0	0	3
irf6	1	1	0	0	1	0	0	3
socs3	1	1	0	0	0	0	1	3
nfx1	1	1	0	0	0	1	0	3
dnaja4	1	1	0	0	1	0	0	3
fzd2	1	1	0	0	0	1	0	3
itgb7	1	0	0	0	1	0	1	3
vrk1	1	1	0	0	0	0	1	3
rnf125	1	0	1	0	0	1	0	3
pcgf3	1	0	0	0	1	0	1	3
slc29a2	1	0	0	0	0	1	1	3
senp6	1	1	0	0	0	1	0	3
pwp2	1	0	1	0	0	0	1	3
akap12	1	1	0	0	0	0	1	3
krt6b	1	1	0	0	0	1	0	3
tgif1	1	1	0	0	1	0	0	3
pcdhb6	1	0	1	1	0	0	0	3

pcyt2	1	1	0	0	1	0	0	3
lims2	1	1	0	0	1	0	0	3
acsbg1	1	1	0	0	0	0	1	3
exoc7	1	1	1	0	0	0	0	3
scd	1	0	1	0	0	1	0	3
plxnc1	1	1	1	0	0	0	0	3
msi2	1	1	0	0	0	0	1	3
nhsl1	1	0	0	1	1	0	0	3
rfwd3	1	0	0	0	1	0	1	3
nfia	1	0	1	0	1	0	0	3
cep162	1	0	1	0	1	0	0	3
aftph	1	1	0	0	0	1	0	3
tnfaip8	1	1	1	0	0	0	0	3
anp32e	1	1	0	0	0	0	1	3
ranbp1	1	0	1	0	0	0	1	3
