# Supplementary material for: Intersection of the Orphan G Protein-Coupled Receptor, GPR19, with the Aging Process
Source: Int J Mol Sci. 2022 Nov 6;23(21):13598. doi: 10.3390/ijms232113598 (PMC9653598; doi:10.3390/ijms232113598)
Supplement: Supplementary file 1 [file ijms-23-13598-s001.zip › Table S2.pdf]

Table S2. Gene Expression Omnibus (GEO) GPR19 associated datasets demonstrating downregulation. The specific GEO datasets (GSE) are indicated and the presence of the associated Gene Symbol for a transcript measured is shown in the different columns.

Gene Symbol	GSE14428	GSE16048	GSE37894	GSE47363	GSE28598	GSE14773	GSE49185	GSE5668	GSE23031	Common Sets
gpr19	1	1	1	1	1	1	1	1	1	9
aurkb	1	0	1	1	1	1	1	1	1	8
ctsb	1	1	1	1	1	1	0	1	1	8
fscn1	1	0	1	1	1	1	1	1	1	8
gart	1	0	1	1	1	1	1	1	1	8
gsg2	1	0	1	1	1	1	1	1	1	8
plpp3	1	0	1	1	1	1	1	1	1	8
tmpo	1	0	1	1	1	1	1	1	1	8
smim19	1	0	1	1	1	1	1	1	1	8
fam102a	1	0	1	1	1	1	1	1	1	8
lamp2	1	0	1	1	1	1	1	1	1	8
itgb3bp	1	0	1	1	1	1	1	1	1	8
mkks	1	0	1	1	1	1	0	1	1	7
atf4	1	0	1	0	1	1	1	1	1	7
pprc1	1	0	1	0	1	1	1	1	1	7
fdft1	1	0	0	1	1	1	1	1	1	7
pbk	1	0	1	0	1	1	1	1	1	7
pxmp2	1	0	1	0	1	1	1	1	1	7
ifnar1	1	0	0	1	1	1	1	1	1	7
ddit4	1	0	1	1	1	1	1	0	1	7
cab39l	1	0	1	0	1	1	1	1	1	7
ogfod3	1	0	1	1	1	1	0	1	1	7
gphn	1	0	1	1	1	1	0	1	1	7
tmem185b	1	0	1	0	1	1	1	1	1	7
mthfd2	1	0	1	0	1	1	1	1	1	7
poglut1	1	0	1	0	1	1	1	1	1	7
smyd3	1	0	0	1	1	1	1	1	1	7
msmo1	1	0	1	1	1	1	1	0	1	7
pmpca	1	0	0	1	1	1	1	1	1	7
rabl6	1	0	1	0	1	1	1	1	1	7
rpa1	1	0	1	1	1	1	1	1	0	7
zxdc	1	0	1	0	1	1	1	1	1	7
vrk1	1	0	1	0	1	1	1	1	1	7
prkacb	1	0	1	1	1	1	1	0	1	7
glg1	1	0	1	0	1	1	1	1	1	7
mief1	1	0	1	1	1	1	0	1	1	7
gnpda2	1	0	1	0	1	1	1	1	1	7
palld	1	1	1	1	1	1	0	1	0	7
gins3	1	0	0	1	1	1	1	1	1	7
pdss2	1	0	0	1	1	1	1	1	1	7

cdk2ap2	1	0	1	0	1	1	1	1	1	7
lrp8	1	0	1	1	1	1	1	0	1	7
cyb561a3	1	0	0	1	1	1	1	1	1	7
nacc2	1	1	1	1	1	0	0	1	1	7
cggbp1	1	0	1	1	1	1	0	1	1	7
pelp1	1	0	1	0	1	1	1	1	1	7
pdzd8	1	1	1	1	1	1	0	1	0	7
tbcd	1	0	0	1	1	1	1	1	1	7
surf4	1	0	0	1	1	1	1	1	1	7
coq2	1	0	1	0	1	1	1	1	1	7
itsn1	1	0	1	1	1	1	0	1	1	7
ctdsp2	1	0	1	0	1	1	1	1	1	7
psat1	1	0	1	1	1	1	1	0	1	7
dynlrb1	1	0	1	1	1	0	1	1	1	7
acaa2	1	0	1	0	1	1	1	1	1	7
msi2	1	0	1	1	1	1	1	0	1	7
zcchc7	1	0	1	0	1	1	1	1	1	7
aplp2	1	0	1	1	1	0	1	1	1	7
snx5	1	0	0	1	1	1	1	1	1	7
irak2	1	0	1	1	1	1	1	0	1	7
plpp5	1	0	1	0	1	1	1	1	1	7
mfng	1	0	1	1	1	1	0	1	1	7
faap100	1	0	1	1	1	0	1	1	1	7
enpp4	1	0	1	1	1	1	1	0	1	7
rabgap1	1	0	1	1	1	1	1	1	0	7
gsk3b	1	0	1	0	1	1	1	1	1	7
mrps33	1	0	1	1	1	1	1	1	0	7
pard6a	1	0	1	1	1	1	0	1	1	7
p2rx4	1	0	1	1	1	0	1	1	1	7
mapkap1	1	0	1	1	1	0	1	1	1	7
mgll	1	0	1	1	1	1	1	0	1	7
lgmn	0	1	1	1	1	0	1	1	1	7
ahnak	0	1	1	1	1	1	1	0	1	7
slc31a1	0	1	1	1	1	1	1	0	1	7
il1rap	0	1	1	1	1	1	0	1	1	7
ivd	0	1	1	0	1	1	1	1	1	7
smad3	0	0	1	1	1	1	1	1	1	7
hbp1	0	0	1	1	1	1	1	1	1	7
mapkapk3	0	0	1	1	1	1	1	1	1	7
bmpr2	0	0	1	1	1	1	1	1	1	7
zmym3	0	0	1	1	1	1	1	1	1	7
carhsp1	0	0	1	1	1	1	1	1	1	7
vps41	0	0	1	1	1	1	1	1	1	7
prmt1	0	0	1	1	1	1	1	1	1	7
mcm5	0	0	1	1	1	1	1	1	1	7

ebp	0	0	1	1	1	1	1	1	1	7
calu	0	0	1	1	1	1	1	1	1	7
elk4	0	0	1	1	1	1	1	1	1	7
aldh2	0	0	1	1	1	1	1	1	1	7
sbno1	0	0	1	1	1	1	1	1	1	7
ypel5	0	0	1	1	1	1	1	1	1	7
cdc25a	0	0	1	1	1	1	1	1	1	7
mprip	0	0	1	1	1	1	1	1	1	7
ercc6l2	0	0	1	1	1	1	1	1	1	7
gins2	0	0	1	1	1	1	1	1	1	7
smc4	0	0	1	1	1	1	1	1	1	7
nasp	0	0	1	1	1	1	1	1	1	7
dnajc10	0	0	1	1	1	1	1	1	1	7
eif2s2	0	0	1	1	1	1	1	1	1	7
spc25	0	0	1	1	1	1	1	1	1	7
aldh18a1	0	0	1	1	1	1	1	1	1	7
nfat5	0	0	1	1	1	1	1	1	1	7
bcl6	0	0	1	1	1	1	1	1	1	7
lrpap1	0	0	1	1	1	1	1	1	1	7
ino80c	0	0	1	1	1	1	1	1	1	7
pdha1	0	0	1	1	1	1	1	1	1	7
mmab	0	0	1	1	1	1	1	1	1	7
tcf4	0	0	1	1	1	1	1	1	1	7
polh	0	0	1	1	1	1	1	1	1	7
ermp1	0	0	1	1	1	1	1	1	1	7
nfia	0	0	1	1	1	1	1	1	1	7
serpine2	0	0	1	1	1	1	1	1	1	7
lmbr1	0	0	1	1	1	1	1	1	1	7
dusp12	0	0	1	1	1	1	1	1	1	7
sars	0	0	1	1	1	1	1	1	1	7
h2afy	0	0	1	1	1	1	1	1	1	7
slc41a3	0	0	1	1	1	1	1	1	1	7
raf1	0	0	1	1	1	1	1	1	1	7
myo19	0	0	1	1	1	1	1	1	1	7
sh3gl1	0	0	1	1	1	1	1	1	1	7
pip4k2b	0	0	1	1	1	1	1	1	1	7
mcm2	0	0	1	1	1	1	1	1	1	7
tpm4	0	0	1	1	1	1	1	1	1	7
sh3glb1	0	0	1	1	1	1	1	1	1	7
msrb1	0	0	1	1	1	1	1	1	1	7
ets2	0	0	1	1	1	1	1	1	1	7
fxr1	0	0	1	1	1	1	1	1	1	7
sdcbp	0	0	1	1	1	1	1	1	1	7
pkmyt1	0	0	1	1	1	1	1	1	1	7
gna13	0	0	1	1	1	1	1	1	1	7

ivns1abp	0	0	1	1	1	1	1	1	1	7
zdhhc24	0	0	1	1	1	1	1	1	1	7
sbf2	0	0	1	1	1	1	1	1	1	7
bcl2l1	0	0	1	1	1	1	1	1	1	7
plekha2	0	0	1	1	1	1	1	1	1	7
map1lc3b	0	0	1	1	1	1	1	1	1	7
tom1l2	0	0	1	1	1	1	1	1	1	7
tmcc1	0	0	1	1	1	1	1	1	1	7
acer3	0	0	1	1	1	1	1	1	1	7
ybx2	1	0	1	0	1	1	0	1	1	6
snx10	1	0	1	1	1	1	0	0	1	6
dpysl3	1	0	0	1	1	1	0	1	1	6
dut	1	0	0	1	1	1	1	0	1	6
sh3rf1	1	0	1	0	1	1	1	0	1	6
snrpb	1	0	1	1	1	1	0	1	0	6
rin2	1	0	1	1	1	0	0	1	1	6
nup88	1	0	1	0	1	1	1	0	1	6
gstk1	1	0	1	0	1	0	1	1	1	6
rapgef1	1	0	1	1	1	0	0	1	1	6
cbx6	1	0	1	1	1	1	0	0	1	6
rfng	1	0	0	1	1	1	1	1	0	6
ppp2r2b	1	1	1	1	1	0	0	1	0	6
asah1	1	0	1	0	1	1	0	1	1	6
abr	1	0	1	1	1	1	1	0	0	6
ppfibp2	1	0	1	1	0	1	0	1	1	6
cds2	1	0	1	0	1	1	0	1	1	6
tctex1d2	1	0	0	1	1	1	0	1	1	6
ncor1	1	0	0	0	1	1	1	1	1	6
sppl2a	1	0	0	0	1	1	1	1	1	6
dusp16	1	0	0	0	1	1	1	1	1	6
nmt2	1	0	0	1	1	1	0	1	1	6
sec22a	1	0	1	0	1	1	0	1	1	6
pde6d	1	0	0	0	1	1	1	1	1	6
hist1h1c	1	0	1	0	1	1	0	1	1	6
vps26b	1	0	0	1	1	1	0	1	1	6
ntm	1	0	1	1	1	1	1	0	0	6
zfp90	1	0	1	1	1	1	0	1	0	6
dlst	1	0	1	1	1	1	0	1	0	6
cited2	1	0	1	1	1	0	1	1	0	6
vps37a	1	0	0	0	1	1	1	1	1	6
trappc1	1	0	0	1	1	0	1	1	1	6
nek1	1	0	1	0	1	1	0	1	1	6
ift43	1	0	0	1	1	1	1	1	0	6
pank2	1	0	0	0	1	1	1	1	1	6
lars2	1	0	0	0	1	1	1	1	1	6

etaa1	1	0	0	0	1	1	1	1	1	6
noa1	1	0	1	0	1	1	1	1	0	6
tmx4	1	0	1	0	1	1	0	1	1	6
wls	1	0	1	1	1	1	0	0	1	6
pfas	1	0	0	1	1	1	1	0	1	6
ppp2r1b	1	0	1	1	1	1	0	0	1	6
nek6	1	0	0	1	1	1	1	0	1	6
parm1	1	0	1	0	1	1	1	0	1	6
h2afz	1	0	0	1	1	1	0	1	1	6
mrps7	1	0	1	1	1	0	0	1	1	6
tgfbi	1	0	1	1	1	1	0	0	1	6
apbb2	1	0	0	1	1	1	1	1	0	6
dtna	1	0	1	1	1	0	0	1	1	6
dhrs1	1	0	1	0	1	1	0	1	1	6
armc7	1	0	0	1	1	1	0	1	1	6
ptpa	1	0	1	1	1	1	0	1	0	6
mastl	1	0	0	1	1	1	1	1	0	6
asns	1	0	0	0	1	1	1	1	1	6
slc30a3	1	0	1	1	1	0	0	1	1	6
sspn	1	0	1	0	1	1	0	1	1	6
prc1	1	0	0	1	1	1	0	1	1	6
ptpn12	1	0	0	1	1	1	1	1	0	6
pkd2	1	0	1	1	1	1	0	0	1	6
pcm1	1	0	0	1	1	1	0	1	1	6
cadm1	1	1	1	1	0	0	0	1	1	6
prepl	1	0	0	0	1	1	1	1	1	6
pitpna	1	0	1	0	1	0	1	1	1	6
dctd	1	0	0	1	1	1	0	1	1	6
gtse1	1	0	1	1	1	1	0	0	1	6
naglu	1	0	0	1	1	0	1	1	1	6
ang	1	0	1	1	1	1	0	0	1	6
csnk1d	1	0	1	1	1	1	0	1	0	6
ubac1	1	0	1	1	1	1	0	1	0	6
nxn	1	0	1	1	1	1	0	1	0	6
alkbh5	1	0	0	1	1	0	1	1	1	6
cnot11	1	0	0	1	1	0	1	1	1	6
lmo4	1	0	1	1	1	1	0	0	1	6
dixdc1	1	0	1	0	1	1	1	0	1	6
dennd1a	1	0	1	1	1	1	0	0	1	6
upf3a	1	0	0	1	1	0	1	1	1	6
napg	1	0	1	1	1	1	0	1	0	6
vps18	1	0	1	1	1	1	0	0	1	6
mmp2	1	0	1	0	1	1	0	1	1	6
agpat2	1	0	1	1	0	1	0	1	1	6
slc7a1	1	0	0	0	1	1	1	1	1	6

f2r	1	0	1	1	1	1	0	1	0	6
camkmt	1	0	0	0	1	1	1	1	1	6
anapc11	1	0	0	1	1	1	0	1	1	6
hsdl2	1	0	0	1	1	1	1	1	0	6
asrgl1	1	0	1	0	1	1	0	1	1	6
phc1	1	0	1	1	1	1	0	1	0	6
kif1b	1	0	1	0	1	1	0	1	1	6
bhlhe40	1	0	1	0	1	1	0	1	1	6
emc9	1	0	1	0	1	1	1	0	1	6
sun1	1	0	0	1	1	1	0	1	1	6
nr2c1	1	0	1	0	1	1	0	1	1	6
kazn	1	0	1	1	1	0	1	1	0	6
g3bp1	1	0	1	1	1	0	1	0	1	6
dido1	1	0	1	0	1	1	0	1	1	6
spcs3	1	0	0	1	1	1	0	1	1	6
nup37	1	0	0	1	1	1	1	1	0	6
gspt2	1	0	1	1	1	0	1	1	0	6
ralbp1	1	0	1	1	1	0	0	1	1	6
ctnnbip1	1	0	1	1	1	1	0	1	0	6
casp6	1	0	1	1	1	0	1	1	0	6
antxr1	1	1	0	1	1	0	1	0	1	6
mrpl48	1	0	0	1	1	1	1	1	0	6
fosl2	1	0	1	0	1	1	1	1	0	6
nectin2	1	0	1	0	1	1	0	1	1	6
depdc1b	1	0	0	1	1	1	0	1	1	6
hipk2	1	0	1	1	1	1	0	1	0	6
dstn	1	0	1	0	1	1	1	1	0	6
fos	1	0	1	1	1	0	0	1	1	6
inpp5e	1	0	1	1	1	1	0	1	0	6
fastkd2	1	0	0	0	1	1	1	1	1	6
atp5o	1	0	0	0	1	1	1	1	1	6
smco4	1	0	1	1	1	1	0	1	0	6
nin	1	0	1	1	1	0	0	1	1	6
arhgef40	1	0	0	1	1	1	1	0	1	6
mpnd	1	0	1	0	1	1	1	1	0	6
dnajb6	1	0	1	0	1	1	0	1	1	6
elp4	1	0	0	0	1	1	1	1	1	6
bnip2	1	0	0	1	1	1	0	1	1	6
sugp2	1	0	0	1	1	1	0	1	1	6
cltb	1	0	1	1	1	0	0	1	1	6
crlf3	1	0	1	0	1	1	1	1	0	6
top1mt	1	0	0	1	1	1	1	0	1	6
paics	1	0	0	0	1	1	1	1	1	6
cipc	1	0	1	1	1	0	1	1	0	6
hadh	1	0	0	0	1	1	1	1	1	6

slx4ip	1	0	1	0	1	1	0	1	1	6
arhgef39	1	0	1	1	0	1	0	1	1	6
tmem62	1	0	1	1	1	0	1	1	0	6
panx1	1	1	0	0	0	1	1	1	1	6
pdlim7	1	0	1	1	1	1	0	0	1	6
mis18a	1	0	0	1	1	1	0	1	1	6
cstf3	1	0	1	1	1	1	0	1	0	6
aldh7a1	1	0	0	1	1	1	0	1	1	6
chchd7	1	0	0	0	1	1	1	1	1	6
gmnn	1	0	0	1	1	1	1	0	1	6
ppp2r2a	1	0	0	0	1	1	1	1	1	6
myo1c	1	0	1	0	1	1	0	1	1	6
slc7a2	1	0	0	0	1	1	1	1	1	6
abcg1	1	0	0	1	1	1	1	1	0	6
tor2a	1	0	0	1	1	0	1	1	1	6
rbms2	1	0	1	0	1	1	0	1	1	6
sec24d	1	0	0	0	1	1	1	1	1	6
phpt1	1	0	1	1	1	0	0	1	1	6
rock1	1	0	0	1	1	0	1	1	1	6
cep131	1	0	1	0	1	1	0	1	1	6
tatdn2	1	0	0	1	1	1	1	1	0	6
srr	1	0	1	0	1	1	0	1	1	6
ptbp1	1	0	0	1	1	1	0	1	1	6
stx12	1	0	1	0	1	1	1	0	1	6
vamp2	1	0	1	1	1	1	0	0	1	6
abi2	1	0	1	0	1	1	0	1	1	6
camsap1	1	0	0	1	1	1	0	1	1	6
trim32	1	0	1	1	1	0	1	1	0	6
aga	1	0	0	1	1	1	1	0	1	6
exoc7	1	0	0	1	1	1	0	1	1	6
rrm2	1	0	1	1	0	1	1	0	1	6
rnf187	1	0	1	0	1	0	1	1	1	6
myh9	1	0	1	1	1	1	0	1	0	6
socs2	0	1	1	0	1	0	1	1	1	6
slc2a13	0	1	1	0	1	1	0	1	1	6
tnip1	0	1	1	0	1	1	0	1	1	6
slc39a11	0	1	1	1	1	0	0	1	1	6
tnfaip8	0	1	0	0	1	1	1	1	1	6
hspb8	0	1	1	1	1	1	0	0	1	6
amigo2	0	1	1	0	1	1	1	0	1	6
minpp1	0	1	1	0	1	1	1	1	0	6
rap2b	0	1	1	0	1	1	1	1	0	6
sdc4	0	1	1	1	1	1	0	0	1	6
soat1	0	1	1	0	1	1	0	1	1	6
smarcb1	0	1	1	0	1	1	1	1	0	6

atf6	0	1	0	1	1	1	0	1	1	6
mcfd2	0	1	1	1	1	0	0	1	1	6
igf1r	0	0	1	0	1	1	1	1	1	6
tns3	0	0	1	1	1	1	1	0	1	6
ctdspl	0	0	1	1	1	1	0	1	1	6
dlgap4	0	0	1	0	1	1	1	1	1	6
furin	0	0	1	1	1	1	1	0	1	6
prmt2	0	0	1	1	1	1	0	1	1	6
sort1	0	0	1	1	1	1	0	1	1	6
abcd3	0	0	1	1	1	1	1	1	0	6
gpt2	0	0	1	0	1	1	1	1	1	6
dcakd	0	0	1	1	1	1	1	1	0	6
gnai2	0	0	1	1	1	0	1	1	1	6
llgl1	0	0	1	1	0	1	1	1	1	6
gsta4	0	0	1	0	1	1	1	1	1	6
gadd45a	0	0	1	1	1	1	1	0	1	6
kdm7a	0	0	1	0	1	1	1	1	1	6
map1b	0	0	1	1	1	1	0	1	1	6
arhgap1	0	0	1	1	1	0	1	1	1	6
pgm2	0	0	1	1	1	1	1	1	0	6
wnk1	0	0	1	0	1	1	1	1	1	6
tcf25	0	0	1	0	1	1	1	1	1	6
kif18b	0	0	1	0	1	1	1	1	1	6
mcmbp	0	0	1	1	1	1	1	1	0	6
fam210b	0	0	1	1	1	1	0	1	1	6
ptcd2	0	0	1	0	1	1	1	1	1	6
bcat2	0	0	1	1	1	1	1	0	1	6
gnb5	0	0	1	1	1	1	0	1	1	6
als2	0	0	1	0	1	1	1	1	1	6
vdac1	0	0	1	1	1	1	1	1	0	6
gdi1	0	0	1	1	1	1	0	1	1	6
mfap3	0	0	1	1	1	1	1	1	0	6
snd1	0	0	1	0	1	1	1	1	1	6
nrp1	0	0	1	1	1	1	0	1	1	6
hp1bp3	0	0	1	1	1	1	1	0	1	6
otud7b	0	0	1	0	1	1	1	1	1	6
ppp2ca	0	0	1	1	1	1	0	1	1	6
card10	0	0	1	1	1	1	0	1	1	6
tnfrsf21	0	0	1	1	1	1	0	1	1	6
atraid	0	0	1	1	1	0	1	1	1	6
tnrc6a	0	0	1	1	1	1	0	1	1	6
pign	0	0	1	1	1	1	1	1	0	6
tdrd3	0	0	1	0	1	1	1	1	1	6
sar1a	0	0	1	1	1	1	0	1	1	6
sh3glb2	0	0	1	0	1	1	1	1	1	6

cdk5	0	0	1	0	1	1	1	1	1	6
yipf6	0	0	1	0	1	1	1	1	1	6
sap30l	0	0	1	1	1	1	1	0	1	6
fchsd2	0	0	1	1	1	0	1	1	1	6
vcl	0	0	1	1	1	1	0	1	1	6
mdm4	0	0	1	0	1	1	1	1	1	6
ext1	0	0	1	1	1	1	1	1	0	6
glo1	0	0	1	1	1	1	1	1	0	6
aldh4a1	0	0	1	1	1	1	1	0	1	6
efl1	0	0	1	1	1	1	0	1	1	6
mapk1	0	0	1	1	1	1	0	1	1	6
mb21d2	0	0	1	1	1	1	1	1	0	6
pgpep1	0	0	1	1	1	1	0	1	1	6
hps4	0	0	1	0	1	1	1	1	1	6
ubap1	0	0	1	1	1	1	1	1	0	6
cnot4	0	0	1	0	1	1	1	1	1	6
urod	0	0	1	0	1	1	1	1	1	6
ccnt2	0	0	1	1	1	1	0	1	1	6
mtus1	0	0	1	1	1	1	0	1	1	6
katnal1	0	0	1	1	1	1	0	1	1	6
plat	0	0	1	1	1	1	1	1	0	6
pex1	0	0	1	1	1	1	1	1	0	6
ocrl	0	0	1	1	1	0	1	1	1	6
evi5	0	0	1	0	1	1	1	1	1	6
adk	0	0	1	1	1	1	0	1	1	6
kdsr	0	0	1	1	1	1	0	1	1	6
cenpn	0	0	1	1	1	1	0	1	1	6
cryz	0	0	1	1	1	1	1	1	0	6
ostm1	0	0	1	1	1	1	0	1	1	6
atg101	0	0	1	0	1	1	1	1	1	6
smurf1	0	0	1	0	1	1	1	1	1	6
endod1	0	0	1	1	1	0	1	1	1	6
kmt5a	0	0	1	1	1	0	1	1	1	6
oxct1	0	0	1	1	1	0	1	1	1	6
mmd	0	0	1	1	1	1	0	1	1	6
bckdhb	0	0	1	1	1	1	1	0	1	6
foxn3	0	0	1	0	1	1	1	1	1	6
rcl1	0	0	1	1	1	1	0	1	1	6
rdm1	0	0	1	0	1	1	1	1	1	6
cyb5b	0	0	1	1	1	1	0	1	1	6
eci1	0	0	1	0	1	1	1	1	1	6
tmed10	0	0	1	1	1	1	0	1	1	6
lpp	0	0	1	1	1	1	1	1	0	6
sept6	0	0	1	1	1	1	1	0	1	6
tsc22d3	0	0	1	1	1	1	1	0	1	6

foxm1	0	0	1	0	1	1	1	1	1	6
rassf1	0	0	1	1	1	1	0	1	1	6
bbs4	0	0	1	1	1	1	0	1	1	6
cenpk	0	0	1	0	1	1	1	1	1	6
smad2	0	0	1	0	1	1	1	1	1	6
nipbl	0	0	1	1	1	1	0	1	1	6
tmem214	0	0	1	1	1	0	1	1	1	6
bcap29	0	0	1	1	1	1	0	1	1	6
tada2a	0	0	1	0	1	1	1	1	1	6
tuft1	0	0	1	1	1	0	1	1	1	6
hnrnpdl	0	0	1	1	1	1	0	1	1	6
mrs2	0	0	1	1	1	1	0	1	1	6
noc2l	0	0	1	1	1	1	0	1	1	6
gaa	0	0	1	1	1	0	1	1	1	6
hibadh	0	0	1	1	1	1	1	1	0	6
rbm8a	0	0	1	0	1	1	1	1	1	6
nsmaf	0	0	1	0	1	1	1	1	1	6
scmh1	0	0	1	0	1	1	1	1	1	6
ccdc18	0	0	1	0	1	1	1	1	1	6
cst3	0	0	1	1	1	1	0	1	1	6
zwint	0	0	1	0	1	1	1	1	1	6
fibp	0	0	1	1	1	1	1	0	1	6
fer	0	0	1	1	1	1	1	1	0	6
aurka	0	0	1	1	1	1	0	1	1	6
dhodh	0	0	1	0	1	1	1	1	1	6
tmed9	0	0	1	1	1	1	1	0	1	6
mta3	0	0	1	1	1	1	1	1	0	6
ccng1	0	0	1	1	1	1	1	1	0	6
rrm1	0	0	1	1	1	1	1	0	1	6
ppp3r1	0	0	1	1	1	1	1	0	1	6
dnmt3b	0	0	1	1	1	1	0	1	1	6
srrd	0	0	1	0	1	1	1	1	1	6
mxra8	0	0	1	1	1	0	1	1	1	6
prim2	0	0	1	1	1	1	1	1	0	6
rrp1b	0	0	1	1	1	0	1	1	1	6
aaas	0	0	1	0	1	1	1	1	1	6
tshz1	0	0	1	0	1	1	1	1	1	6
l2hgdh	0	0	1	1	1	1	1	0	1	6
pcca	0	0	1	1	1	1	1	1	0	6
ide	0	0	1	0	1	1	1	1	1	6
lig3	0	0	1	0	1	1	1	1	1	6
fyn	0	0	1	0	1	1	1	1	1	6
nfatc2ip	0	0	1	1	1	1	0	1	1	6
rbfa	0	0	1	0	1	1	1	1	1	6
dbt	0	0	1	1	1	1	1	0	1	6

ndst1	0	0	1	1	1	1	0	1	1	6
rnf32	0	0	1	0	1	1	1	1	1	6
hddc2	0	0	1	1	1	1	0	1	1	6
cdc16	0	0	1	1	1	0	1	1	1	6
psen1	0	0	1	0	1	1	1	1	1	6
hacd3	0	0	1	1	1	1	0	1	1	6
kat5	0	0	1	0	1	1	1	1	1	6
acbd3	0	0	1	1	1	0	1	1	1	6
igf2bp3	0	0	1	1	1	1	0	1	1	6
esyt2	0	0	1	1	1	1	0	1	1	6
vps13a	0	0	1	1	1	1	0	1	1	6
kcmf1	0	0	1	0	1	1	1	1	1	6
srxn1	0	0	1	1	1	1	1	0	1	6
sar1b	0	0	1	0	1	1	1	1	1	6
ndrg3	0	0	1	1	1	1	0	1	1	6
rbbp9	0	0	1	1	1	1	1	0	1	6
wdr37	0	0	1	1	1	1	0	1	1	6
mrpl23	0	0	1	1	1	1	0	1	1	6
myh10	0	0	1	0	1	1	1	1	1	6
usp36	0	0	1	0	1	1	1	1	1	6
isca1	0	0	1	1	1	1	1	1	0	6
sertad2	0	0	1	1	1	1	0	1	1	6
nfe2l1	0	0	1	0	1	1	1	1	1	6
mcm7	0	0	1	0	1	1	1	1	1	6
ckap2l	0	0	1	1	1	1	0	1	1	6
mphosph9	0	0	1	0	1	1	1	1	1	6
bcl2l13	0	0	1	1	1	0	1	1	1	6
tmed3	0	0	1	0	1	1	1	1	1	6
klc1	0	0	1	1	1	1	0	1	1	6
rabgap1l	0	0	1	0	1	1	1	1	1	6
tbc1d2	0	0	1	1	1	1	1	0	1	6
ate1	0	0	1	1	1	1	1	1	0	6
fitm2	0	0	1	1	1	0	1	1	1	6
akirin1	0	0	1	1	1	1	0	1	1	6
ccnd1	0	0	1	1	1	1	0	1	1	6
cyth2	0	0	1	0	1	1	1	1	1	6
pdpr	0	0	1	1	1	1	1	1	0	6
rala	0	0	1	0	1	1	1	1	1	6
zc3h10	0	0	1	1	1	0	1	1	1	6
oma1	0	0	1	0	1	1	1	1	1	6
hmgcr	0	0	1	1	1	1	1	1	0	6
tsen2	0	0	1	1	1	0	1	1	1	6
ptbp2	0	0	1	1	1	1	1	0	1	6
hsbp1	0	0	1	1	1	1	1	1	0	6
slc10a7	0	0	1	1	1	1	0	1	1	6

rad51c	0	0	1	1	1	1	0	1	1	6
slc44a1	0	0	1	1	1	1	0	1	1	6
jade2	0	0	1	1	1	1	0	1	1	6
lancl1	0	0	1	1	1	1	1	1	0	6
atl3	0	0	1	0	1	1	1	1	1	6
chrna5	0	0	1	1	1	1	1	0	1	6
ncaph2	0	0	1	0	1	1	1	1	1	6
tle1	0	0	1	1	1	1	0	1	1	6
ift22	0	0	1	0	1	1	1	1	1	6
ppm1l	0	0	1	0	1	1	1	1	1	6
tnfaip1	0	0	1	1	1	1	0	1	1	6
blm	0	0	1	0	1	1	1	1	1	6
ldha	0	0	1	0	1	1	1	1	1	6
troap	0	0	1	0	1	1	1	1	1	6
gdpd1	0	0	1	0	1	1	1	1	1	6
ntpcr	0	0	1	1	1	1	0	1	1	6
pole	0	0	1	0	1	1	1	1	1	6
yme1l1	0	0	1	1	1	0	1	1	1	6
fbxo28	0	0	1	0	1	1	1	1	1	6
idnk	0	0	1	0	1	1	1	1	1	6
ttll4	0	0	1	1	1	1	1	1	0	6
cdv3	0	0	1	1	1	1	0	1	1	6
kctd1	0	0	1	0	1	1	1	1	1	6
mcl1	0	0	1	0	1	1	1	1	1	6
fnip1	0	0	1	1	1	1	0	1	1	6
lyplal1	0	0	1	0	1	1	1	1	1	6
ddx11	0	0	1	1	1	1	0	1	1	6
ppp2r5d	0	0	1	1	1	1	1	1	0	6
tm7sf3	0	0	1	1	1	1	1	0	1	6
srsf10	0	0	1	0	1	1	1	1	1	6
diexf	0	0	1	0	1	1	1	1	1	6
ylpm1	0	0	1	0	1	1	1	1	1	6
pold1	0	0	1	0	1	1	1	1	1	6
lpin1	0	0	1	1	1	1	0	1	1	6
smarcc2	0	0	1	0	1	1	1	1	1	6
naa25	0	0	1	0	1	1	1	1	1	6
oxnad1	0	0	1	1	1	0	1	1	1	6
usp42	0	0	1	1	1	1	0	1	1	6
arrb2	0	0	1	0	1	1	1	1	1	6
thg1l	0	0	1	0	1	1	1	1	1	6
cenpm	0	0	1	0	1	1	1	1	1	6
srsf6	0	0	1	1	1	0	1	1	1	6
cdca5	0	0	1	1	1	1	1	0	1	6
uchl5	0	0	1	0	1	1	1	1	1	6
susd6	0	0	1	1	1	1	1	0	1	6

melk	0	0	1	1	0	1	1	1	1	6
crebrf	0	0	1	1	1	0	1	1	1	6
hagh	0	0	1	1	1	0	1	1	1	6
nectin3	0	0	1	1	1	1	0	1	1	6
mcm4	0	0	1	1	1	1	1	0	1	6
rbm33	0	0	1	0	1	1	1	1	1	6
zfand2a	0	0	1	1	1	1	1	1	0	6
cebpb	0	0	1	1	1	0	1	1	1	6
ift27	0	0	1	0	1	1	1	1	1	6
e2f2	0	0	1	1	1	1	0	1	1	6
dzip3	0	0	1	0	1	1	1	1	1	6
ubqln1	0	0	1	1	1	1	0	1	1	6
acsl1	0	0	1	0	1	1	1	1	1	6
sdhc	0	0	1	1	1	1	1	1	0	6
leprotl1	0	0	1	1	1	1	0	1	1	6
hipk3	0	0	1	1	1	1	1	0	1	6
cdpf1	0	0	1	0	1	1	1	1	1	6
lgalsl	0	0	1	0	1	1	1	1	1	6
cenpw	0	0	1	0	1	1	1	1	1	6
icmt	0	0	1	1	1	1	0	1	1	6
psmc3ip	0	0	1	1	1	1	0	1	1	6
hnrnpul1	0	0	1	1	1	1	0	1	1	6
ilf3	0	0	1	0	1	1	1	1	1	6
cdc25c	0	0	1	1	1	1	0	1	1	6
thnsl1	0	0	1	1	1	1	1	0	1	6
sec22c	0	0	1	1	0	1	1	1	1	6
acyp1	0	0	1	1	1	1	0	1	1	6
dpp7	0	0	1	1	1	1	0	1	1	6
reep5	0	0	1	1	1	1	0	1	1	6
bcdin3d	0	0	1	1	1	1	1	0	1	6
rida	0	0	1	1	1	1	1	1	0	6
blmh	0	0	1	0	1	1	1	1	1	6
brca1	0	0	1	0	1	1	1	1	1	6
bax	0	0	1	1	1	1	0	1	1	6
itgb1bp1	0	0	1	1	1	1	1	0	1	6
idh1	0	0	1	1	1	1	1	0	1	6
eif1ad	0	0	1	0	1	1	1	1	1	6
cul4b	0	0	1	1	1	1	1	0	1	6
asxl1	0	0	1	1	1	1	0	1	1	6
cdc37l1	0	0	1	0	1	1	1	1	1	6
spop	0	0	1	0	1	1	1	1	1	6
whsc1	0	0	1	0	1	1	1	1	1	6
cetn3	0	0	1	0	1	1	1	1	1	6
b4gat1	0	0	1	1	1	1	0	1	1	6
acbd6	0	0	1	1	1	1	0	1	1	6

eefsec	0	0	1	0	1	1	1	1	1	6
golph3l	0	0	1	1	1	1	1	1	0	6
stx17	0	0	1	0	1	1	1	1	1	6
mrpl28	0	0	1	1	1	1	0	1	1	6
efcab14	0	0	1	0	1	1	1	1	1	6
sesn3	0	0	1	1	1	1	0	1	1	6
ppp1r15a	0	0	1	0	1	1	1	1	1	6
ergic1	0	0	1	1	1	1	1	0	1	6
ndel1	0	0	1	0	1	1	1	1	1	6
mast2	0	0	1	1	1	0	1	1	1	6
srm	0	0	1	1	1	0	1	1	1	6
thop1	0	0	1	0	1	1	1	1	1	6
rfc2	0	0	1	1	1	1	1	1	0	6
commd7	0	0	1	1	1	1	0	1	1	6
rapgef2	0	0	1	0	1	1	1	1	1	6
tubg1	0	0	1	1	1	1	1	0	1	6
dennd5b	0	0	1	1	1	1	1	0	1	6
lss	0	0	1	1	1	1	0	1	1	6
mto1	0	0	1	1	1	0	1	1	1	6
anapc16	0	0	1	0	1	1	1	1	1	6
rnaseh2a	0	0	1	1	1	1	1	1	0	6
ccdc86	0	0	1	1	1	1	0	1	1	6
xpr1	0	0	1	1	1	1	1	0	1	6
camsap2	0	0	1	0	1	1	1	1	1	6
tfip11	0	0	1	1	1	1	0	1	1	6
e2f6	0	0	1	1	1	1	0	1	1	6
kif23	0	0	1	0	1	1	1	1	1	6
cep57	0	0	1	0	1	1	1	1	1	6
kctd3	0	0	1	1	1	1	1	0	1	6
eprs	0	0	1	0	1	1	1	1	1	6
nek4	0	0	1	0	1	1	1	1	1	6
nucks1	0	0	1	1	1	1	0	1	1	6
rsl24d1	0	0	1	0	1	1	1	1	1	6
ercc2	0	0	1	1	1	1	1	0	1	6
adam17	0	0	1	1	1	1	1	1	0	6
cpt2	0	0	1	0	1	1	1	1	1	6
cdca7	0	0	1	1	1	1	1	0	1	6
pcgf5	0	0	1	1	1	1	0	1	1	6
rab2a	0	0	1	1	1	1	1	0	1	6
tmem63b	0	0	1	1	1	0	1	1	1	6
pold3	0	0	1	0	1	1	1	1	1	6
psip1	0	0	1	0	1	1	1	1	1	6
xpo4	0	0	1	1	1	0	1	1	1	6
uqcc1	0	0	1	1	1	0	1	1	1	6
kif20b	0	0	1	0	1	1	1	1	1	6

tcfl5	0	0	1	1	1	1	0	1	1	6
rptor	0	0	1	0	1	1	1	1	1	6
mkrn1	0	0	1	0	1	1	1	1	1	6
arfgap1	0	0	1	1	1	1	0	1	1	6
e2f5	0	0	1	0	1	1	1	1	1	6
cep89	0	0	1	1	1	1	0	1	1	6
thoc5	0	0	1	1	1	1	0	1	1	6
aprt	0	0	1	1	1	0	1	1	1	6
phf20	0	0	1	1	1	1	0	1	1	6
fsd1	0	0	1	1	1	0	1	1	1	6
nudt5	0	0	1	1	1	1	0	1	1	6
rab21	0	0	1	1	1	0	1	1	1	6
p3h1	0	0	1	1	1	1	1	1	0	6
clip4	0	0	1	1	1	1	0	1	1	6
rnf141	0	0	1	0	1	1	1	1	1	6
dicer1	0	0	1	1	1	1	0	1	1	6
xpot	0	0	1	0	1	1	1	1	1	6
atp6v1c1	0	0	1	1	1	1	0	1	1	6
tmem116	0	0	1	1	1	0	1	1	1	6
nudt7	0	0	1	0	1	1	1	1	1	6
usp21	0	0	1	1	1	1	0	1	1	6
ube3b	0	0	1	0	1	1	1	1	1	6
csnk2a2	0	0	1	1	1	1	1	1	0	6
nusap1	0	0	1	1	1	1	0	1	1	6
pawr	0	0	1	0	1	1	1	1	1	6
atf7ip	0	0	1	0	1	1	1	1	1	6
uba5	0	0	1	1	1	1	0	1	1	6
ankle2	0	0	1	1	1	1	0	1	1	6
ensa	0	0	1	1	1	1	0	1	1	6
slc15a4	0	0	1	1	1	0	1	1	1	6
alg9	0	0	1	1	1	1	0	1	1	6
ifngr1	0	0	1	1	1	1	1	0	1	6
ppif	0	0	1	1	1	1	0	1	1	6
sass6	0	0	1	0	1	1	1	1	1	6
bclaf1	0	0	1	1	1	1	0	1	1	6
fem1c	0	0	1	1	1	1	1	1	0	6
prkaca	0	0	1	0	1	1	1	1	1	6
nit1	0	0	1	0	1	1	1	1	1	6
cnnm2	0	0	1	0	1	1	1	1	1	6
cwf19l1	0	0	1	1	1	0	1	1	1	6
nap1l1	0	0	1	1	1	1	0	1	1	6
edem2	0	0	1	1	1	1	1	1	0	6
necap2	0	0	1	1	1	1	0	1	1	6
mettl1	0	0	1	0	1	1	1	1	1	6
chmp1b	0	0	1	1	1	1	1	0	1	6

ndufaf5	0	0	1	1	1	0	1	1	1	6
spag9	0	0	1	1	1	1	1	0	1	6
scarb1	0	0	1	0	1	1	1	1	1	6
cnih1	0	0	1	1	1	1	0	1	1	6
cdc42	0	0	1	1	1	1	0	1	1	6
l3mbtl2	0	0	1	1	1	0	1	1	1	6
slc19a1	0	0	1	0	1	1	1	1	1	6
dcaf7	0	0	1	1	1	1	0	1	1	6
jun	0	0	1	1	1	1	1	0	1	6
atf5	0	0	1	1	1	1	1	0	1	6
faf1	0	0	1	1	1	1	0	1	1	6
ube2h	0	0	1	1	1	1	1	0	1	6
derl1	0	0	0	1	1	1	1	1	1	6
pgk1	0	0	0	1	1	1	1	1	1	6
ube3c	0	0	0	1	1	1	1	1	1	6
polr3h	0	0	0	1	1	1	1	1	1	6
xrcc5	0	0	0	1	1	1	1	1	1	6
rit1	0	0	0	1	1	1	1	1	1	6
mcm6	0	0	0	1	1	1	1	1	1	6
rnf26	0	0	0	1	1	1	1	1	1	6
imp4	0	0	0	1	1	1	1	1	1	6
eef1a1	0	0	0	1	1	1	1	1	1	6
tomm20	0	0	0	1	1	1	1	1	1	6
mtmr6	0	0	0	1	1	1	1	1	1	6
pgp	0	0	0	1	1	1	1	1	1	6
rbl1	0	0	0	1	1	1	1	1	1	6
cdca4	0	0	0	1	1	1	1	1	1	6
mib2	0	0	0	1	1	1	1	1	1	6
h2afx	0	0	0	1	1	1	1	1	1	6
fanci	0	0	0	1	1	1	1	1	1	6
bre	0	0	0	1	1	1	1	1	1	6
mcm10	0	0	0	1	1	1	1	1	1	6
snap23	0	0	0	1	1	1	1	1	1	6
stx2	0	0	0	1	1	1	1	1	1	6
kif11	0	0	0	1	1	1	1	1	1	6
exo1	0	0	0	1	1	1	1	1	1	6
fen1	0	0	0	1	1	1	1	1	1	6
gss	0	0	0	1	1	1	1	1	1	6
fbxo9	0	0	0	1	1	1	1	1	1	6
gdap2	0	0	0	1	1	1	1	1	1	6
ankib1	0	0	0	1	1	1	1	1	1	6
rnmt	0	0	0	1	1	1	1	1	1	6
wac	0	0	0	1	1	1	1	1	1	6
cbll1	0	0	0	1	1	1	1	1	1	6
hspa5	0	0	0	1	1	1	1	1	1	6

cdc42bpa	0	0	0	1	1	1	1	1	1	6
rpl37a	0	0	0	1	1	1	1	1	1	6
coq10b	0	0	0	1	1	1	1	1	1	6
rhno1	0	0	0	1	1	1	1	1	1	6
rmnd5a	0	0	0	1	1	1	1	1	1	6
mturn	0	0	0	1	1	1	1	1	1	6
mcm3	0	0	0	1	1	1	1	1	1	6
sec63	0	0	0	1	1	1	1	1	1	6
dlgap5	0	0	0	1	1	1	1	1	1	6
golgb1	0	0	0	1	1	1	1	1	1	6
eif4b	0	0	0	1	1	1	1	1	1	6
zbtb44	0	0	0	1	1	1	1	1	1	6
slc7a11	0	0	0	1	1	1	1	1	1	6
lactb2	0	0	0	1	1	1	1	1	1	6
mrpl35	0	0	0	1	1	1	1	1	1	6
kif22	0	0	0	1	1	1	1	1	1	6
zak	0	0	0	1	1	1	1	1	1	6
ccnf	0	0	0	1	1	1	1	1	1	6
eef1d	0	0	0	1	1	1	1	1	1	6
pard3	0	0	0	1	1	1	1	1	1	6
il6st	0	0	0	1	1	1	1	1	1	6
chchd3	0	0	0	1	1	1	1	1	1	6
pphln1	0	0	0	1	1	1	1	1	1	6
recql4	0	0	0	1	1	1	1	1	1	6
mtdh	0	0	0	1	1	1	1	1	1	6
cand1	0	0	0	1	1	1	1	1	1	6
fam46c	0	0	0	1	1	1	1	1	1	6
stmn1	0	0	0	1	1	1	1	1	1	6
gramd4	0	0	0	1	1	1	1	1	1	6
slc12a2	0	0	0	1	1	1	1	1	1	6
jund	0	0	0	1	1	1	1	1	1	6
rad54l	0	0	0	1	1	1	1	1	1	6
fanca	0	0	0	1	1	1	1	1	1	6
rrp12	0	0	0	1	1	1	1	1	1	6
prim1	0	0	0	1	1	1	1	1	1	6
ints2	0	0	0	1	1	1	1	1	1	6
p4ha1	0	0	0	1	1	1	1	1	1	6
btbd7	0	0	0	1	1	1	1	1	1	6
ythdf2	0	0	0	1	1	1	1	1	1	6
bmp2k	0	0	0	1	1	1	1	1	1	6
fam160b1	0	0	0	1	1	1	1	1	1	6
agbl5	0	0	0	1	1	1	1	1	1	6
atad2	0	0	0	1	1	1	1	1	1	6
lcmt2	0	0	0	1	1	1	1	1	1	6
ccni	0	0	0	1	1	1	1	1	1	6

gpatch2	0	0	0	1	1	1	1	1	1	6
haus8	0	0	0	1	1	1	1	1	1	6
tpgs2	0	0	0	1	1	1	1	1	1	6
dtymk	0	0	0	1	1	1	1	1	1	6
higd1a	0	0	0	1	1	1	1	1	1	6
fem1b	0	0	0	1	1	1	1	1	1	6
gclm	0	0	0	1	1	1	1	1	1	6
ncor2	0	0	0	1	1	1	1	1	1	6
cebpg	0	0	0	1	1	1	1	1	1	6
ska2	0	0	0	1	1	1	1	1	1	6
gclc	0	0	0	1	1	1	1	1	1	6
chaf1b	0	0	0	1	1	1	1	1	1	6
rbm18	0	0	0	1	1	1	1	1	1	6
jmy	0	0	0	1	1	1	1	1	1	6
tug1	0	0	0	1	1	1	1	1	1	6
hnrnpu	0	0	0	1	1	1	1	1	1	6
cenpe	0	0	0	1	1	1	1	1	1	6
hnrnpll	0	0	0	1	1	1	1	1	1	6
cdc6	0	0	0	1	1	1	1	1	1	6
cse1l	0	0	0	1	1	1	1	1	1	6
slco2a1	1	0	1	1	1	0	0	0	1	5
cnot7	1	0	0	1	1	0	0	1	1	5
sun2	1	0	1	1	1	0	0	0	1	5
zfp82	1	0	1	0	1	1	0	1	0	5
tyro3	1	0	1	1	1	0	0	0	1	5
hrasls	1	0	1	1	1	1	0	0	0	5
tusc3	1	0	0	1	1	1	0	1	0	5
ints10	1	0	1	0	1	0	1	1	0	5
kpna1	1	0	0	1	1	1	0	0	1	5
frmd5	1	0	1	1	1	1	0	0	0	5
fkbp4	1	0	0	0	1	1	0	1	1	5
rgs17	1	0	0	1	1	1	0	1	0	5
rragd	1	0	1	0	1	0	1	0	1	5
smim14	1	0	0	1	1	0	0	1	1	5
slc2a8	1	0	1	1	1	0	0	0	1	5
tmem8a	1	0	0	1	0	1	1	0	1	5
crk	1	0	1	0	1	1	0	1	0	5
ak4	1	1	0	1	1	0	0	1	0	5
h2afj	1	0	0	0	1	1	0	1	1	5
dfna5	1	0	0	1	1	0	0	1	1	5
snrpb2	1	0	0	1	1	0	0	1	1	5
bcl7c	1	0	0	1	1	0	0	1	1	5
lias	1	0	0	0	1	1	1	0	1	5
rab40b	1	0	0	1	1	1	0	0	1	5
rora	1	0	0	1	1	1	0	1	0	5

nmrk1	1	0	0	1	1	1	0	0	1	5
nek3	1	0	0	0	1	1	1	1	0	5
cd109	1	0	0	0	1	1	1	0	1	5
mepce	1	0	1	0	1	1	0	1	0	5
polb	1	0	1	0	1	0	0	1	1	5
c3	1	1	0	0	1	1	0	0	1	5
snapc4	1	0	0	1	0	0	1	1	1	5
cryzl1	1	0	0	0	1	1	1	1	0	5
peli2	1	0	1	0	1	1	1	0	0	5
wdr45b	1	0	1	1	1	1	0	0	0	5
cbs	1	1	0	1	1	0	0	0	1	5
sec61a2	1	0	0	1	1	0	0	1	1	5
arrdc1	1	0	0	0	1	0	1	1	1	5
ddias	1	0	0	0	1	1	1	1	0	5
mbnl3	1	0	0	0	1	1	0	1	1	5
cog1	1	0	1	0	1	0	0	1	1	5
tbk1	1	0	1	1	1	1	0	0	0	5
b4galnt1	1	0	1	1	1	0	0	0	1	5
ddrgk1	1	0	1	1	1	0	0	1	0	5
plcxd1	1	0	0	1	1	1	0	0	1	5
ccna2	1	0	0	0	0	1	1	1	1	5
adck2	1	0	0	0	1	1	0	1	1	5
sept9	1	0	1	0	1	1	0	0	1	5
agpat5	1	0	0	0	1	0	1	1	1	5
fkbp1a	1	0	0	1	1	0	1	1	0	5
get4	1	0	1	0	1	0	0	1	1	5
mrps26	1	0	0	0	1	0	1	1	1	5
gsto2	1	0	1	1	0	1	0	1	0	5
fignl1	1	0	0	1	1	1	0	0	1	5
dock10	1	0	0	1	1	0	1	1	0	5
tmem107	1	0	0	0	1	1	1	0	1	5
shroom3	1	0	0	1	1	1	0	1	0	5
chrnb1	1	0	1	1	1	0	0	0	1	5
e2f8	1	0	0	0	1	1	1	0	1	5
dlc1	1	0	0	1	1	0	1	0	1	5
cpsf4	1	0	0	1	0	1	0	1	1	5
ccdc62	1	0	1	1	0	0	1	1	0	5
snrpd1	1	0	1	1	0	1	0	0	1	5
ncoa1	1	0	0	1	1	0	0	1	1	5
lrrc1	1	0	1	0	0	1	0	1	1	5
fam49a	1	0	0	0	1	1	1	1	0	5
ggps1	1	0	0	0	1	1	1	1	0	5
gab2	1	0	0	1	1	0	1	1	0	5
drg2	1	0	1	0	1	0	0	1	1	5
tmem256	1	0	1	0	1	0	1	1	0	5

cops3	1	0	0	1	1	0	0	1	1	5
cant1	1	0	1	1	1	0	1	0	0	5
ccdc92	1	0	0	1	1	1	1	0	0	5
bmp1	1	0	1	1	1	0	0	0	1	5
tubb2b	1	0	0	1	1	1	0	1	0	5
ephb4	1	0	1	0	1	1	0	0	1	5
slc30a9	1	0	0	1	1	1	0	0	1	5
trim8	1	0	1	1	1	0	0	0	1	5
cds1	1	0	1	1	0	0	1	1	0	5
nup62cl	1	0	0	1	0	1	1	0	1	5
ube2k	1	0	0	0	1	1	0	1	1	5
nrip3	1	0	1	1	1	0	0	0	1	5
slc20a2	1	0	0	0	1	1	0	1	1	5
abl1	1	0	1	0	1	0	1	1	0	5
lama1	1	0	1	0	1	1	0	1	0	5
f2rl2	1	0	0	1	1	0	1	0	1	5
ndrg1	1	0	1	0	1	1	0	0	1	5
dus1l	1	0	0	0	1	0	1	1	1	5
tpp1	1	0	1	0	1	0	0	1	1	5
arfgap3	1	0	1	0	1	1	1	0	0	5
pros1	1	1	1	1	1	0	0	0	0	5
serinc3	1	0	1	0	1	0	0	1	1	5
add3	1	0	1	1	1	1	0	0	0	5
itm2c	1	0	0	0	1	1	0	1	1	5
epn2	1	0	0	1	1	0	1	1	0	5
slc25a11	1	0	1	0	1	0	1	1	0	5
c1qbp	1	0	1	0	1	0	0	1	1	5
rabepk	1	0	0	1	1	0	0	1	1	5
pdia5	1	0	1	1	0	0	1	1	0	5
actr10	1	0	1	0	1	0	0	1	1	5
donson	1	0	1	0	1	1	1	0	0	5
il10rb	1	0	1	0	1	0	0	1	1	5
ube2l6	1	0	0	0	1	1	1	0	1	5
taok2	1	0	1	0	1	1	0	1	0	5
nfxl1	1	0	0	0	1	0	1	1	1	5
atp6v1a	1	0	1	1	1	0	0	1	0	5
timm10	1	0	0	0	1	0	1	1	1	5
camk1	1	0	1	1	1	0	0	0	1	5
eif4a1	1	0	1	1	1	0	0	1	0	5
mylk	1	0	1	1	0	1	0	0	1	5
colgalt1	1	0	1	1	1	0	0	0	1	5
rtn4ip1	1	0	0	0	1	1	1	0	1	5
rcc1	1	0	0	0	1	1	0	1	1	5
idh3b	1	0	0	1	1	1	0	1	0	5
ralgds	1	0	1	1	1	0	0	1	0	5

smpdl3b	1	0	1	1	0	1	0	0	1	5
tesk2	1	0	0	0	1	0	1	1	1	5
mettl22	1	0	1	0	1	0	0	1	1	5
tk2	1	0	0	0	1	1	0	1	1	5
prkch	1	0	0	1	1	1	0	0	1	5
prosc	1	0	1	1	1	0	0	1	0	5
notch1	1	0	1	1	1	0	0	0	1	5
itgae	1	0	1	0	1	1	0	1	0	5
tubb4b	1	0	0	1	1	0	1	1	0	5
plcb1	1	0	0	0	1	1	0	1	1	5
fpgs	1	0	0	0	1	0	1	1	1	5
spag5	1	0	0	0	1	1	0	1	1	5
lancl2	1	0	0	1	1	0	0	1	1	5
hspa9	1	0	1	0	1	0	1	0	1	5
pcyox1	1	0	1	0	1	0	0	1	1	5
slc25a15	1	0	0	1	1	0	1	0	1	5
ccnc	1	0	1	0	1	0	0	1	1	5
kdelr2	1	0	0	1	1	1	1	0	0	5
ccdc59	1	0	1	0	1	0	1	0	1	5
slc25a37	1	0	0	0	1	1	1	0	1	5
sat2	1	0	1	0	1	1	0	0	1	5
sdha	1	0	0	1	1	0	0	1	1	5
adipor1	1	0	1	1	1	0	0	1	0	5
ehmt2	1	0	0	0	1	1	1	0	1	5
dcaf4	1	0	1	1	1	0	0	1	0	5
rhbdd2	1	0	0	0	1	1	0	1	1	5
eif4e3	1	0	0	0	1	1	1	0	1	5
nfatc3	1	0	1	0	1	1	0	0	1	5
acox1	1	0	0	1	1	1	0	0	1	5
ppm1d	1	0	0	1	1	1	1	0	0	5
psmf1	1	0	0	0	1	1	0	1	1	5
polr2b	1	0	0	1	1	0	1	1	0	5
zmynd19	1	0	0	0	1	0	1	1	1	5
slc38a10	1	0	0	0	1	1	1	0	1	5
fam175b	1	0	1	0	1	1	0	0	1	5
ptges2	1	0	0	0	1	1	1	1	0	5
adprm	1	0	0	0	1	0	1	1	1	5
sdhaf2	1	0	0	0	1	1	1	1	0	5
mak16	1	0	0	0	1	1	0	1	1	5
sh3bgrl2	1	0	0	0	1	1	0	1	1	5
lsm14b	1	0	1	0	1	0	1	0	1	5
zmynd8	1	0	1	0	1	0	1	1	0	5
arpp19	1	0	1	1	1	0	0	1	0	5
ddr1	1	0	1	1	1	0	0	1	0	5
cript	1	0	0	0	1	0	1	1	1	5

gstm4	1	0	1	1	0	1	0	0	1	5
pld1	1	0	0	0	1	1	0	1	1	5
fam171a1	1	0	1	1	1	0	0	1	0	5
nsfl1c	1	0	0	1	1	0	0	1	1	5
apmap	1	0	1	1	1	0	0	0	1	5
mrpl12	1	0	0	0	1	0	1	1	1	5
ppp1r3c	1	0	1	1	1	0	0	0	1	5
coq8a	1	0	0	1	1	0	1	0	1	5
thyn1	1	0	0	0	1	1	0	1	1	5
fam198b	1	0	1	1	1	1	0	0	0	5
tnfrsf1a	1	0	1	0	0	1	1	0	1	5
ndufv2	1	0	0	0	1	0	1	1	1	5
lef1	1	0	1	1	1	0	0	1	0	5
def8	1	0	0	1	1	0	0	1	1	5
ctsc	1	0	0	1	1	1	0	0	1	5
rpp25l	1	0	1	0	1	0	0	1	1	5
linc00339	1	0	0	1	0	1	1	0	1	5
nup50	1	0	0	0	1	1	0	1	1	5
thoc1	1	0	1	0	1	0	0	1	1	5
ifrd1	1	0	0	1	1	0	1	0	1	5
man1b1	1	0	1	0	1	0	1	0	1	5
cyb5d1	1	0	0	0	1	0	1	1	1	5
nars2	1	0	0	0	1	1	1	0	1	5
glmp	1	0	1	0	1	0	0	1	1	5
errfi1	1	0	1	1	1	1	0	0	0	5
umps	1	0	0	0	1	0	1	1	1	5
xbp1	1	0	1	0	1	0	1	0	1	5
ei24	1	0	1	1	1	0	0	1	0	5
eif2ak4	1	0	0	0	1	1	0	1	1	5
dpm2	1	0	0	1	1	0	0	1	1	5
cntnap1	1	0	0	1	1	1	0	0	1	5
fbln1	1	0	1	0	1	1	0	0	1	5
cdip1	1	0	1	0	1	0	0	1	1	5
cd2bp2	1	0	0	0	1	1	0	1	1	5
ddx31	1	0	0	1	1	0	0	1	1	5
pcna	1	0	0	1	1	1	0	0	1	5
msrb2	1	0	0	0	1	1	1	0	1	5
uckl1	1	0	0	0	1	1	0	1	1	5
map1a	1	0	0	1	1	1	0	0	1	5
ncstn	1	0	0	1	1	1	1	0	0	5
cbx2	1	0	0	1	1	1	0	0	1	5
agps	1	0	1	0	1	1	0	0	1	5
phf23	1	0	0	0	1	1	0	1	1	5
rcn3	1	0	1	1	1	0	0	0	1	5
ppp3cc	1	0	1	0	1	0	0	1	1	5

prdm1	1	0	0	1	1	1	0	1	0	5
rgs10	1	0	1	0	1	0	1	1	0	5
pex16	1	0	1	1	1	0	0	1	0	5
hirip3	1	0	0	0	1	1	1	0	1	5
gmfb	1	0	0	1	1	1	0	1	0	5
elp5	1	0	1	0	1	0	0	1	1	5
slc5a6	1	0	1	1	0	0	1	0	1	5
atg4a	1	0	0	1	1	0	1	1	0	5
ube2e3	1	0	1	0	1	0	0	1	1	5
fasn	1	0	0	0	1	1	1	0	1	5
slc22a5	1	0	0	1	1	1	0	0	1	5
lamp1	1	0	0	0	1	0	1	1	1	5
bub3	1	0	0	0	1	1	0	1	1	5
socs4	1	0	0	1	1	1	0	1	0	5
bhlhb9	1	0	1	0	1	1	0	0	1	5
