# Supplementary material for: Intersection of the Orphan G Protein-Coupled Receptor, GPR19, with the Aging Process
Source: Int J Mol Sci. 2022 Nov 6;23(21):13598. doi: 10.3390/ijms232113598 (PMC9653598; doi:10.3390/ijms232113598)
Supplement: Supplementary file 1 [file ijms-23-13598-s001.zip › Table S3.pdf]

**Table S3.** Gene Ontology Term Enrichment Analysis. Using the Molecular Signatures Database (MSigDB) a Gene Ontology enrichment analysis was performed with GeneTrail v3.2 (https://genetrail.bioinf.uni-sb.de/). For each specifically enriched GO term (at least two independent proteins per term at a p value of >0.05) the total number of input hits (from the uploaded dataset), the expected number of hits as well as the adjusted p value of enrichment (plus the negative log10 version of this) is shown.

| GO Term | Number of hits | Expected score | Adjusted p-value | Negative Log p-value |
|---|---|---|---|---|
| GO AGING | 50 | 3.52737 | 1.05127E-35 | 34.97828573 |
| GO REGULATION OF CELL DEATH | 79 | 18.6561 | 7.36466E-25 | 24.1328473 |
| GO RESPONSE TO OXYGEN CONTAINING COMPOUND | 76 | 18.0262 | 1.05775E-23 | 22.97561697 |
| GO RESPONSE TO OXIDATIVE STRESS | 43 | 4.90167 | 7.10949E-23 | 22.14816155 |
| GO RESPONSE TO ABIOTIC STIMULUS | 65 | 13.7888 | 3.60039E-22 | 21.44365045 |
| GO CELLULAR RESPONSE TO DNA DAMAGE STIMULUS | 55 | 9.55138 | 4.87102E-22 | 21.31238009 |
| GO POSITIVE REGULATION OF PROTEIN METABOLIC PROCESS | 73 | 18.1179 | 1.0093E-21 | 20.99597973 |
| GO CELLULAR RESPONSE TO OXYGEN CONTAINING COMPOUND | 61 | 12.4832 | 1.78602E-21 | 20.74811368 |
| GO APOPTOTIC PROCESS | 78 | 21.6796 | 9.7341E-21 | 20.0117042 |
| GO NEURON DEATH | 37 | 3.8824 | 9.7341E-21 | 20.0117042 |
| GO DNA METABOLIC PROCESS | 53 | 9.53993 | 1.16535E-20 | 19.93354362 |
| GO REGULATION OF PROTEIN MODIFICATION PROCESS | 75 | 20.2595 | 1.57219E-20 | 19.80349497 |
| GO CELLULAR RESPONSE TO OXIDATIVE STRESS | 34 | 3.2296 | 3.29551E-20 | 19.48207737 |
| GO REGULATION OF RESPONSE TO STRESS | 67 | 16.3427 | 3.46277E-20 | 19.46057635 |
| GO NEGATIVE REGULATION OF CELL DEATH | 55 | 11.0173 | 1.27547E-19 | 18.89432975 |
| GO CELL AGING | 24 | 1.19106 | 1.72489E-19 | 18.7632386 |
| GO POSITIVE REGULATION OF BIOSYNTHETIC PROCESS | 77 | 22.3896 | 1.72489E-19 | 18.7632386 |
| GO POSITIVE REGULATION OF CELLULAR COMPONENT ORGANIZATION | 60 | 13.4338 | 1.72489E-19 | 18.7632386 |
| GO CELLULAR RESPONSE TO NITROGEN COMPOUND | 45 | 7.112 | 2.3312E-19 | 18.63242047 |
| GO RESPONSE TO NITROGEN COMPOUND | 54 | 10.9142 | 3.7816E-19 | 18.42232441 |
| GO HOMEOSTATIC PROCESS | 74 | 21.3131 | 8.03674E-19 | 18.09492008 |
| GO POSITIVE REGULATION OF PROTEIN MODIFICATION PROCESS | 59 | 13.5483 | 1.13037E-18 | 17.94677938 |
| GO REGULATION OF CELL POPULATION PROLIFERATION | 67 | 18.0377 | 3.83693E-18 | 17.41601612 |
| GO RESPONSE TO INORGANIC SUBSTANCE | 41 | 6.25306 | 5.18387E-18 | 17.2853459 |
| GO RESPONSE TO ENDOGENOUS STIMULUS | 67 | 18.324 | 8.00759E-18 | 17.09649817 |
| GO NEGATIVE REGULATION OF RESPONSE TO STIMULUS | 66 | 17.8774 | 1.00838E-17 | 16.99637578 |
| GO REGULATION OF CELLULAR RESPONSE TO STRESS | 45 | 7.94803 | 1.05275E-17 | 16.97767475 |
| GO DNA REPAIR | 40 | 6.13854 | 1.68656E-17 | 16.7729982 |
| GO POSITIVE REGULATION OF CELL DEATH | 44 | 7.66172 | 1.68656E-17 | 16.7729982 |
| GO REGULATION OF PHOSPHORUS METABOLIC PROCESS | 66 | 18.6218 | 6.97793E-17 | 16.15627339 |
| GO NEGATIVE REGULATION OF PROTEIN METABOLIC PROCESS | 52 | 11.7846 | 2.03335E-16 | 15.69178786 |
| GO REGULATION OF CATABOLIC PROCESS | 50 | 10.8799 | 2.18673E-16 | 15.66020484 |
| GO REGULATION OF CELLULAR CATABOLIC PROCESS | 46 | 9.12764 | 2.4163E-16 | 15.61684915 |
| GO RESPONSE TO REACTIVE OXYGEN SPECIES | 27 | 2.49664 | 3.11725E-16 | 15.50622837 |
| GO POSITIVE REGULATION OF PHOSPHORUS METABOLIC PROCESS | 52 | 12.0137 | 3.98929E-16 | 15.39910439 |
| GO REACTIVE OXYGEN SPECIES METABOLIC PROCESS | 24 | 1.8324 | 5.66731E-16 | 15.24662303 |

| | | | | |
|---|---|---|---|---|
| GO AMYLOID BETA METABOLIC PROCESS | 16 | 0.4581 | 7.32308E-16 | 15.13530622 |
| GO POSITIVE REGULATION OF CATABOLIC PROCESS | 34 | 4.74133 | 7.95912E-16 | 15.09913495 |
| GO REGULATION OF INTRACELLULAR SIGNAL TRANSDUCTION | 67 | 20.2824 | 9.28549E-16 | 15.03219517 |
| GO CELLULAR RESPONSE TO ENDOGENOUS STIMULUS | 58 | 15.3807 | 1.06098E-15 | 14.9742928 |
| GO RESPONSE TO DRUG | 50 | 11.4754 | 1.4265E-15 | 14.84572822 |
| GO RESPONSE TO PEPTIDE | 36 | 5.62318 | 1.9422E-15 | 14.71170605 |
| GO CHROMOSOME ORGANIZATION | 54 | 13.5483 | 1.95997E-15 | 14.70775058 |
| GO POSITIVE REGULATION OF DEVELOPMENTAL PROCESS | 57 | 15.1746 | 2.438E-15 | 14.6129663 |
| GO REGULATION OF REACTIVE OXYGEN SPECIES METABOLIC PROCESS | 20 | 1.14525 | 2.82016E-15 | 14.54972625 |
| GO REGULATION OF TRANSFERASE ACTIVITY | 48 | 10.7768 | 2.98908E-15 | 14.52446246 |
| GO POSITIVE REGULATION OF SIGNALING | 66 | 20.3167 | 3.47562E-15 | 14.45896771 |
| GO GROWTH | 48 | 10.9715 | 5.66367E-15 | 14.24690206 |
| GO PROTEIN PHOSPHORYLATION | 68 | 21.7941 | 6.78852E-15 | 14.1682249 |
| GO NEGATIVE REGULATION OF DEVELOPMENTAL PROCESS | 47 | 10.6165 | 8.13817E-15 | 14.08947324 |
| GO RESPONSE TO EXTRACELLULAR STIMULUS | 36 | 5.9553 | 8.90904E-15 | 14.05016909 |
| GO CELLULAR RESPONSE TO TOXIC SUBSTANCE | 26 | 2.65698 | 8.92744E-15 | 14.04927306 |
| GO NEGATIVE REGULATION OF CELLULAR COMPONENT ORGANIZATION | 40 | 7.73044 | 1.87151E-14 | 13.72780785 |
| GO RESPONSE TO OXYGEN LEVELS | 31 | 4.3405 | 2.3398E-14 | 13.63082126 |
| GO RESPONSE TO TOXIC SUBSTANCE | 35 | 5.80642 | 2.62557E-14 | 13.5807764 |
| GO MITOCHONDRION ORGANIZATION | 35 | 5.85223 | 3.18059E-14 | 13.49749231 |
| GO PROCESS UTILIZING AUTOPHAGIC MECHANISM | 34 | 5.47429 | 3.18059E-14 | 13.49749231 |
| GO REGULATION OF GROWTH | 39 | 7.58155 | 5.3581E-14 | 13.27098919 |
| GO CELLULAR RESPONSE TO PEPTIDE | 30 | 4.18016 | 6.28238E-14 | 13.2018758 |
| GO PEPTIDE METABOLIC PROCESS | 19 | 1.20251 | 7.51537E-14 | 13.12404963 |
| GO NEURON APOPTOTIC PROCESS | 25 | 2.67988 | 9.06777E-14 | 13.0424995 |
| GO POSITIVE REGULATION OF GENE EXPRESSION | 66 | 22.0461 | 1.40773E-13 | 12.85148063 |
| GO RESPONSE TO IONIZING RADIATION | 21 | 1.69497 | 1.4397E-13 | 12.841728 |
| GO RESPONSE TO RADIATION | 32 | 5.07346 | 1.65409E-13 | 12.78144086 |
| GO REGULATION OF TRANSPORT | 63 | 20.4656 | 2.21128E-13 | 12.65535626 |
| GO CELLULAR SENESCENCE | 16 | 0.755865 | 3.17479E-13 | 12.498285 |
| GO NEGATIVE REGULATION OF SIGNALING | 53 | 15.0371 | 4.09352E-13 | 12.38790308 |
| GO NEGATIVE REGULATION OF NEURON DEATH | 23 | 2.33631 | 4.69863E-13 | 12.32802875 |
| GO POSITIVE REGULATION OF MOLECULAR FUNCTION | 61 | 19.7556 | 6.1172E-13 | 12.21344732 |
| GO NEGATIVE REGULATION OF BIOSYNTHETIC PROCESS | 57 | 17.4765 | 6.734E-13 | 12.17172689 |
| GO POSITIVE REGULATION OF MULTICELLULAR ORGANISMAL PROCESS | 60 | 19.4005 | 1.01404E-12 | 11.99394491 |
| GO REGULATION OF CELLULAR COMPONENT BIOGENESIS | 43 | 10.25 | 1.02383E-12 | 11.98977215 |
| GO CELLULAR RESPONSE TO DRUG | 28 | 4.01983 | 1.12515E-12 | 11.94878958 |
| GO REGULATION OF MITOCHONDRION ORGANIZATION | 21 | 1.99273 | 2.28824E-12 | 11.64049843 |
| GO AMYLOID PRECURSOR PROTEIN CATABOLIC PROCESS | 13 | 0.423742 | 2.63889E-12 | 11.57857871 |
| GO REGULATION OF KINASE ACTIVITY | 41 | 9.643 | 3.07644E-12 | 11.51195155 |
| GO REGULATION OF DNA METABOLIC PROCESS | 25 | 3.21815 | 3.53458E-12 | 11.45166218 |
| GO REGULATION OF PROTEOLYSIS | 37 | 7.89077 | 4.1371E-12 | 11.38330398 |
| GO AMYLOID PRECURSOR PROTEIN METABOLIC PROCESS | 14 | 0.606982 | 6.28986E-12 | 11.20135902 |
| GO DOUBLE STRAND BREAK REPAIR | 23 | 2.71424 | 7.60665E-12 | 11.11880657 |
| GO REGULATION OF CELLULAR LOCALIZATION | 41 | 9.99803 | 9.15208E-12 | 11.03848019 |

| | | | | |
|---|---|---|---|---|
| GO NEUROINFLAMMATORY RESPONSE | 14 | 0.64134 | 1.16137E-11 | 10.9350294 |
| GO CELLULAR RESPONSE TO IONIZING RADIATION | 15 | 0.813127 | 1.18403E-11 | 10.92663729 |
| GO POSITIVE REGULATION OF ORGANELLE ORGANIZATION | 34 | 6.98602 | 1.86283E-11 | 10.72982678 |
| GO GLIAL CELL ACTIVATION | 13 | 0.515362 | 1.91189E-11 | 10.7185371 |
| GO REGULATION OF CELL AGING | 13 | 0.526815 | 2.39965E-11 | 10.6198521 |
| GO RESPONSE TO ORGANIC CYCLIC COMPOUND | 41 | 10.3645 | 2.66167E-11 | 10.57484579 |
| GO CELLULAR RESPONSE TO REACTIVE OXYGEN SPECIES | 19 | 1.77514 | 2.9689E-11 | 10.52740443 |
| GO REGULATION OF AUTOPHAGY | 25 | 3.58463 | 2.9689E-11 | 10.52740443 |
| GO REGULATION OF CELLULAR PROTEIN LOCALIZATION | 31 | 5.88658 | 3.45592E-11 | 10.46143632 |
| GO REGULATION OF ORGANELLE ORGANIZATION | 48 | 14.1553 | 3.57014E-11 | 10.44731475 |
| GO POSITIVE REGULATION OF TRANSFERASE ACTIVITY | 34 | 7.28379 | 5.26443E-11 | 10.27864864 |
| GO NEGATIVE REGULATION OF MULTICELLULAR ORGANISMAL PROCESS | 46 | 13.3651 | 7.52634E-11 | 10.12341617 |
| GO AMYLOID BETA FORMATION | 11 | 0.32067 | 9.41395E-11 | 10.02622811 |
| GO REGULATION OF BINDING | 26 | 4.18016 | 1.03213E-10 | 9.986265599 |
| GO POSITIVE REGULATION OF RNA BIOSYNTHETIC PROCESS | 54 | 18.1866 | 1.29244E-10 | 9.888589609 |
| GO RESPONSE TO GAMMA RADIATION | 13 | 0.629887 | 1.50748E-10 | 9.821748441 |
| GO POSITIVE REGULATION OF PROTEOLYSIS | 25 | 3.9053 | 1.60135E-10 | 9.795513736 |
| GO REGULATION OF PROTEIN LOCALIZATION | 41 | 11.086 | 1.91211E-10 | 9.718487127 |
| GO BEHAVIOR | 32 | 6.79133 | 2.09493E-10 | 9.678830484 |
| GO NEGATIVE REGULATION OF PHOSPHORYLATION | 27 | 4.7986 | 3.07884E-10 | 9.51161288 |
| GO INFLAMMATORY RESPONSE | 34 | 7.87932 | 3.88262E-10 | 9.410875113 |
| GO ANATOMICAL STRUCTURE HOMEOSTASIS | 27 | 4.85586 | 3.91941E-10 | 9.406779304 |
| GO POSITIVE REGULATION OF CATALYTIC ACTIVITY | 49 | 15.8503 | 4.50353E-10 | 9.34644694 |
| GO CELLULAR HOMEOSTASIS | 40 | 10.9257 | 4.82258E-10 | 9.316720559 |
| GO REGULATION OF PROTEIN STABILITY | 22 | 3.06927 | 4.82258E-10 | 9.316720559 |
| GO RESPONSE TO TEMPERATURE STIMULUS | 21 | 2.7486 | 5.00981E-10 | 9.300178745 |
| GO DNA BIOSYNTHETIC PROCESS | 19 | 2.14162 | 5.17982E-10 | 9.285685332 |
| GO REGULATION OF NUCLEOBASE CONTAINING COMPOUND METABOLIC PROCESS | 29 | 5.76061 | 5.36396E-10 | 9.270514469 |
| GO REGULATION OF RESPONSE TO EXTERNAL STIMULUS | 35 | 8.56647 | 7.18023E-10 | 9.143861644 |
| GO LEUKOCYTE ACTIVATION INVOLVED IN INFLAMMATORY RESPONSE | 11 | 0.423742 | 9.82923E-10 | 9.007480502 |
| GO REGULATION OF MAPK CASCADE | 34 | 8.2687 | 1.26106E-09 | 8.89926425 |
| GO RESPONSE TO ANTIBIOTIC | 23 | 3.60754 | 1.31742E-09 | 8.880275748 |
| GO POSITIVE REGULATION OF NEURON DEATH | 14 | 0.996367 | 1.62774E-09 | 8.788414964 |
| GO REGULATION OF DEVELOPMENTAL GROWTH | 23 | 3.6648 | 1.74681E-09 | 8.757754331 |
| GO PROTEOLYSIS | 54 | 19.6181 | 1.8914E-09 | 8.723216615 |
| GO REGULATION OF AMYLOID PRECURSOR PROTEIN CATABOLIC PROCESS | 10 | 0.32067 | 1.97202E-09 | 8.705088685 |
| GO REGULATION OF PROTEIN COMPLEX ASSEMBLY | 26 | 4.85586 | 2.01236E-09 | 8.696294324 |
| GO POSITIVE REGULATION OF BINDING | 18 | 2.0729 | 2.51479E-09 | 8.599498275 |
| GO RESPONSE TO HORMONE | 39 | 11.0631 | 2.51479E-09 | 8.599498275 |
| GO REGULATION OF RESPONSE TO DNA DAMAGE STIMULUS | 19 | 2.38212 | 2.57379E-09 | 8.589426891 |
| GO RESPONSE TO HYDROGEN PEROXIDE | 16 | 1.52318 | 2.58203E-09 | 8.588038716 |
| GO CELLULAR RESPONSE TO INORGANIC SUBSTANCE | 19 | 2.43938 | 3.70739E-09 | 8.430931726 |
| GO NEGATIVE REGULATION OF CELL POPULATION PROLIFERATION | 32 | 7.68463 | 3.73485E-09 | 8.427726836 |
| GO CELLULAR RESPONSE TO RADIATION | 18 | 2.14162 | 3.92678E-09 | 8.40596343 |
| GO REGULATION OF PROTEIN CATABOLIC PROCESS | 24 | 4.22597 | 3.92678E-09 | 8.40596343 |

| | | | | |
|---|---|---|---|---|
| GO DEVELOPMENTAL GROWTH | 31 | 7.24943 | 4.10584E-09 | 8.386597979 |
| GO RESPONSE TO X RAY | 10 | 0.355027 | 4.1499E-09 | 8.381962368 |
| GO POSITIVE REGULATION OF TRANSPORT | 38 | 10.7997 | 4.69646E-09 | 8.328229372 |
| GO DNA RECOMBINATION | 21 | 3.18379 | 5.46688E-09 | 8.262260459 |
| GO POSITIVE REGULATION OF KINASE ACTIVITY | 29 | 6.4363 | 5.72286E-09 | 8.242386878 |
| GO PROTEIN LOCALIZATION TO ORGANELLE | 37 | 10.376 | 6.03154E-09 | 8.219571788 |
| GO POSITIVE REGULATION OF REACTIVE OXYGEN SPECIES METABOLIC PROCESS | 11 | 0.526815 | 6.12658E-09 | 8.212781891 |
| GO NEGATIVE REGULATION OF DNA METABOLIC PROCESS | 14 | 1.1338 | 6.56819E-09 | 8.182554293 |
| GO REGULATION OF RESPONSE TO OXIDATIVE STRESS | 13 | 0.9162 | 6.98649E-09 | 8.155740958 |
| GO REGULATION OF AMYLOID BETA FORMATION | 9 | 0.251955 | 7.06557E-09 | 8.150852797 |
| GO NEGATIVE REGULATION OF PROTEIN MODIFICATION PROCESS | 29 | 6.52792 | 7.54337E-09 | 8.12243459 |
| GO RESPONSE TO PEPTIDE HORMONE | 25 | 4.7986 | 7.66345E-09 | 8.115575672 |
| GO TELOMERE ORGANIZATION | 17 | 1.96983 | 8.88172E-09 | 8.051502922 |
| GO NEGATIVE REGULATION OF AMYLOID PRECURSOR PROTEIN CATABOLIC PROCESS | 8 | 0.160335 | 1.07892E-08 | 7.967010756 |
| GO NEGATIVE REGULATION OF CATABOLIC PROCESS | 21 | 3.33268 | 1.11605E-08 | 7.952316348 |
| GO RESPONSE TO HEAT | 17 | 2.00419 | 1.11605E-08 | 7.952316348 |
| GO POSITIVE REGULATION OF INTRACELLULAR SIGNAL TRANSDUCTION | 38 | 11.1891 | 1.14272E-08 | 7.942060171 |
| GO APOPTOTIC SIGNALING PATHWAY | 29 | 6.68826 | 1.24229E-08 | 7.905777011 |
| GO NEGATIVE REGULATION OF NUCLEOBASE CONTAINING COMPOUND METABOLIC PROCESS | 16 | 1.74078 | 1.35193E-08 | 7.869045795 |
| GO REGULATION OF CHROMOSOME ORGANIZATION | 22 | 3.77932 | 1.55197E-08 | 7.809116678 |
| GO REGULATION OF NERVOUS SYSTEM DEVELOPMENT | 36 | 10.25 | 1.55197E-08 | 7.809116678 |
| GO REGULATION OF PROTEASOMAL PROTEIN CATABOLIC PROCESS | 17 | 2.06145 | 1.60745E-08 | 7.793862527 |
| GO CELLULAR RESPONSE TO HYDROGEN PEROXIDE | 13 | 0.996367 | 1.61728E-08 | 7.791214784 |
| GO REGULATION OF CELL DIFFERENTIATION | 53 | 20.4656 | 2.15232E-08 | 7.667093159 |
| GO REGULATION OF OXIDATIVE STRESS INDUCED INTRINSIC APOPTOTIC SIGNALING PATHWAY | 9 | 0.297765 | 2.15232E-08 | 7.667093159 |
| GO NEGATIVE REGULATION OF PHOSPHORUS METABOLIC PROCESS | 27 | 5.96675 | 2.27139E-08 | 7.643708291 |
| GO REGULATION OF CELLULAR PROTEIN CATABOLIC PROCESS | 19 | 2.7715 | 2.28146E-08 | 7.641787141 |
| GO POSITIVE REGULATION OF PROTEIN CATABOLIC PROCESS | 18 | 2.43938 | 2.31602E-08 | 7.635257695 |
| GO RESPONSE TO AMYLOID BETA | 10 | 0.446647 | 2.32114E-08 | 7.634298664 |
| GO MUSCLE CELL PROLIFERATION | 17 | 2.13016 | 2.4444E-08 | 7.611827725 |
| GO CELLULAR RESPONSE TO ANTIBIOTIC | 15 | 1.54609 | 2.46428E-08 | 7.608309948 |
| GO PEPTIDYL AMINO ACID MODIFICATION | 42 | 13.8804 | 2.87645E-08 | 7.541143171 |
| GO DNA GEOMETRIC CHANGE | 13 | 1.06508 | 3.19507E-08 | 7.495519623 |
| GO SIGNAL TRANSDUCTION BY PROTEIN PHOSPHORYLATION | 36 | 10.5707 | 3.2246E-08 | 7.49152415 |
| GO REGULATION OF CELLULAR AMIDE METABOLIC PROCESS | 23 | 4.3634 | 3.3042E-08 | 7.480933673 |
| GO CELLULAR RESPONSE TO ORGANIC CYCLIC COMPOUND | 27 | 6.09273 | 3.3133E-08 | 7.47973924 |
| GO CELLULAR RESPONSE TO OXYGEN LEVELS | 18 | 2.5081 | 3.32828E-08 | 7.477780145 |
| GO BASE EXCISION REPAIR | 10 | 0.469552 | 3.3443E-08 | 7.475694771 |
| GO RESPONSE TO ANTINEOPLASTIC AGENT | 13 | 1.07653 | 3.46995E-08 | 7.459676783 |
| GO NEGATIVE REGULATION OF APOPTOTIC SIGNALING PATHWAY | 18 | 2.531 | 3.748E-08 | 7.426200418 |
| GO POSITIVE REGULATION OF CELLULAR PROTEIN LOCALIZATION | 21 | 3.61899 | 3.90876E-08 | 7.407960995 |
| GO POSITIVE REGULATION OF CELLULAR PROTEIN CATABOLIC PROCESS | 15 | 1.6148 | 4.02043E-08 | 7.395727495 |
| GO RESPONSE TO OXYGEN RADICAL | 9 | 0.332122 | 4.29197E-08 | 7.367343322 |
| GO NEGATIVE REGULATION OF MOLECULAR FUNCTION | 39 | 12.4145 | 4.69601E-08 | 7.328270987 |
| GO REGULATION OF APOPTOTIC SIGNALING PATHWAY | 23 | 4.46647 | 4.77093E-08 | 7.321396955 |

| | | | | |
|---|---|---|---|---|
| GO RESPONSE TO COPPER ION | 10 | 0.492457 | 4.77093E-08 | 7.321396955 |
| GO CELLULAR AMIDE METABOLIC PROCESS | 36 | 10.7768 | 4.95394E-08 | 7.305049258 |
| GO REGULATION OF INFLAMMATORY RESPONSE | 21 | 3.69916 | 5.46031E-08 | 7.2627827 |
| GO REGULATION OF OXIDATIVE STRESS INDUCED CELL DEATH | 11 | 0.68715 | 5.62406E-08 | 7.249950055 |
| GO CELL CELL SIGNALING | 49 | 18.576 | 5.98472E-08 | 7.222956164 |
| GO MUSCLE CELL MIGRATION | 12 | 0.904747 | 6.10455E-08 | 7.214346345 |
| GO RESPONSE TO LIPID | 35 | 10.3531 | 6.4411E-08 | 7.191039958 |
| GO RESPONSE TO METAL ION | 22 | 4.16871 | 7.31438E-08 | 7.135822481 |
| GO REGULATION OF CELL DEVELOPMENT | 35 | 10.4103 | 7.33217E-08 | 7.134767474 |
| GO CARBOHYDRATE HOMEOSTASIS | 18 | 2.66843 | 7.60662E-08 | 7.118808279 |
| GO ASTROCYTE ACTIVATION | 8 | 0.22905 | 7.60855E-08 | 7.118698101 |
| GO CELL CYCLE PROCESS | 43 | 15.1402 | 9.67067E-08 | 7.014543436 |
| GO NEGATIVE REGULATION OF CELLULAR CATABOLIC PROCESS | 18 | 2.71424 | 9.67067E-08 | 7.014543436 |
| GO NEGATIVE REGULATION OF ORGANELLE ORGANIZATION | 22 | 4.24888 | 9.91249E-08 | 7.003817238 |
| GO GLIAL CELL DEVELOPMENT | 13 | 1.21396 | 1.17764E-07 | 6.928987451 |
| GO REGULATION OF IMMUNE SYSTEM PROCESS | 46 | 17.0986 | 1.18135E-07 | 6.927621414 |
| GO NEGATIVE REGULATION OF CELL DIFFERENTIATION | 30 | 7.99384 | 1.19478E-07 | 6.922712056 |
| GO NEGATIVE REGULATION OF INTRACELLULAR SIGNAL TRANSDUCTION | 25 | 5.60027 | 1.19736E-07 | 6.921775254 |
| GO SMOOTH MUSCLE CELL MIGRATION | 11 | 0.755865 | 1.26699E-07 | 6.897226813 |
| GO SUPEROXIDE METABOLIC PROCESS | 11 | 0.755865 | 1.26699E-07 | 6.897226813 |
| GO NEGATIVE REGULATION OF MITOCHONDRION ORGANIZATION | 10 | 0.561172 | 1.29789E-07 | 6.886762114 |
| GO POSITIVE REGULATION OF DNA METABOLIC PROCESS | 15 | 1.79804 | 1.37934E-07 | 6.860328669 |
| GO CELLULAR RESPONSE TO PEPTIDE HORMONE STIMULUS | 20 | 3.53882 | 1.40045E-07 | 6.853732392 |
| GO REGULATION OF PROTEIN SERINE THREONINE KINASE ACTIVITY | 25 | 5.66899 | 1.47195E-07 | 6.832106942 |
| GO PROTEIN CONTAINING COMPLEX ASSEMBLY | 52 | 21.1184 | 1.47216E-07 | 6.832044987 |
| GO REGULATION OF PEPTIDYL TYROSINE PHOSPHORYLATION | 18 | 2.80586 | 1.47968E-07 | 6.829832196 |
| GO SMOOTH MUSCLE CELL PROLIFERATION | 14 | 1.52318 | 1.53845E-07 | 6.812916614 |
| GO POSITIVE REGULATION OF CELL DIFFERENTIATION | 35 | 10.7653 | 1.55184E-07 | 6.809153058 |
| GO REPLICATIVE SENESCENCE | 7 | 0.148882 | 1.68665E-07 | 6.772975029 |
| GO CHEMICAL HOMEOSTASIS | 39 | 13.0902 | 1.71341E-07 | 6.766138703 |
| GO REGULATION OF CELL CYCLE | 39 | 13.2391 | 2.32003E-07 | 6.634506399 |
| GO POSITIVE REGULATION OF AUTOPHAGY | 13 | 1.30558 | 2.42671E-07 | 6.61498212 |
| GO DEFENSE RESPONSE | 47 | 18.2324 | 2.61856E-07 | 6.581937471 |
| GO POSITIVE REGULATION OF MITOCHONDRION ORGANIZATION | 13 | 1.31704 | 2.64818E-07 | 6.577052499 |
| GO ASTROCYTE DEVELOPMENT | 9 | 0.435195 | 2.68135E-07 | 6.571646493 |
| GO REGULATION OF TRANS SYNAPTIC SIGNALING | 23 | 4.95893 | 2.68135E-07 | 6.571646493 |
| GO MACROAUTOPHAGY | 19 | 3.30977 | 2.71114E-07 | 6.566848055 |
| GO REGULATION OF MACROAUTOPHAGY | 15 | 1.91257 | 2.78815E-07 | 6.554683865 |
| GO PROTEIN COMPLEX OLIGOMERIZATION | 26 | 6.36759 | 2.92249E-07 | 6.534246966 |
| GO TRANSMEMBRANE RECEPTOR PROTEIN TYROSINE KINASE SIGNALING PATHWAY | 29 | 7.87932 | 3.17137E-07 | 6.498753086 |
| GO POSITIVE REGULATION OF NUCLEOBASE CONTAINING COMPOUND METABOLIC PROCESS | 17 | 2.61117 | 3.26492E-07 | 6.486127456 |
| GO REGULATION OF DNA REPAIR | 13 | 1.35139 | 3.39334E-07 | 6.469372623 |
| GO POSITIVE REGULATION OF MAPK CASCADE | 25 | 5.9553 | 3.48116E-07 | 6.458276015 |
| GO CIRCULATORY SYSTEM DEVELOPMENT | 36 | 11.7274 | 3.53179E-07 | 6.452005127 |
| GO DNA CONFORMATION CHANGE | 19 | 3.38994 | 3.77984E-07 | 6.422526583 |

| | | | | |
|---|---|---|---|---|
| GO CELLULAR GLUCOSE HOMEOSTASIS | 14 | 1.66061 | 3.92663E-07 | 6.40598002 |
| GO POSITIVE REGULATION OF CELL DEVELOPMENT | 25 | 6.00111 | 3.95977E-07 | 6.402330039 |
| GO MUSCLE STRUCTURE DEVELOPMENT | 27 | 6.97457 | 4.04342E-07 | 6.393251145 |
| GO MACROMOLECULE DEACYLATION | 12 | 1.11089 | 4.07893E-07 | 6.389453748 |
| GO RESPONSE TO ACID CHEMICAL | 20 | 3.81368 | 4.12264E-07 | 6.384824587 |
| GO POSITIVE REGULATION OF PROTEOLYSIS INVOLVED IN CELLULAR PROTEIN CATABOLIC PROCESS | 13 | 1.38575 | 4.30227E-07 | 6.366302338 |
| GO NEGATIVE REGULATION OF NEURON APOPTOTIC PROCESS | 14 | 1.68352 | 4.49564E-07 | 6.347208473 |
| GO RELEASE OF CYTOCHROME C FROM MITOCHONDRIA | 10 | 0.664245 | 4.71055E-07 | 6.326928382 |
| GO CELL DEATH IN RESPONSE TO OXIDATIVE STRESS | 11 | 0.893295 | 5.21932E-07 | 6.282386075 |
| GO INTRINSIC APOPTOTIC SIGNALING PATHWAY IN RESPONSE TO OXIDATIVE STRESS | 9 | 0.481005 | 5.27208E-07 | 6.278018008 |
| GO STRESS INDUCED PREMATURE SENESCENCE | 6 | 0.09162 | 5.48528E-07 | 6.260801199 |
| GO RESPONSE TO LIGHT STIMULUS | 19 | 3.51592 | 6.27875E-07 | 6.202126809 |
| GO ENZYME LINKED RECEPTOR PROTEIN SIGNALING PATHWAY | 35 | 11.4525 | 6.3043E-07 | 6.200363128 |
| GO OXIDATION REDUCTION PROCESS | 34 | 10.8913 | 6.38442E-07 | 6.194878551 |
| GO REGULATION OF ESTABLISHMENT OF PROTEIN LOCALIZATION | 29 | 8.17708 | 6.43011E-07 | 6.191781598 |
| GO RECOMBINATIONAL REPAIR | 13 | 1.44301 | 6.44856E-07 | 6.190537255 |
| GO CELLULAR RESPONSE TO GAMMA RADIATION | 8 | 0.332122 | 6.76269E-07 | 6.16988052 |
| GO CELL GROWTH | 23 | 5.26815 | 7.01861E-07 | 6.153748889 |
| GO TELOMERE MAINTENANCE VIA TELOMERE LENGTHENING | 11 | 0.927652 | 7.13983E-07 | 6.146312129 |
| GO POSITIVE REGULATION OF PEPTIDYL TYROSINE PHOSPHORYLATION | 15 | 2.10726 | 8.37876E-07 | 6.076820249 |
| GO NEGATIVE REGULATION OF OXIDATIVE STRESS INDUCED INTRINSIC APOPTOTIC SIGNALING PATHWAY | 7 | 0.206145 | 8.41092E-07 | 6.075156498 |
| GO REGULATION OF INTRINSIC APOPTOTIC SIGNALING PATHWAY | 14 | 1.78659 | 8.51191E-07 | 6.069972977 |
| GO TELOMERIC D LOOP DISASSEMBLY | 6 | 0.103072 | 8.60115E-07 | 6.065443478 |
| GO CELL CYCLE | 49 | 20.3854 | 8.75956E-07 | 6.057517708 |
| GO REGULATION OF DEFENSE RESPONSE | 29 | 8.31451 | 8.793E-07 | 6.055862927 |
| GO CELLULAR RESPONSE TO HORMONE STIMULUS | 28 | 7.82206 | 9.45733E-07 | 6.024231457 |
| GO INTRINSIC APOPTOTIC SIGNALING PATHWAY | 18 | 3.2296 | 9.55525E-07 | 6.019757946 |
| GO MACROPHAGE ACTIVATION | 11 | 0.96201 | 9.69663E-07 | 6.013379176 |
| GO REGULATION OF NEUROINFLAMMATORY RESPONSE | 8 | 0.355027 | 1.00103E-06 | 5.999552907 |
| GO RESPONSE TO ACTIVITY | 10 | 0.73296 | 1.00103E-06 | 5.999552907 |
| GO CHROMATIN ORGANIZATION | 30 | 8.91004 | 1.00499E-06 | 5.99783826 |
| GO MUSCLE ADAPTATION | 12 | 1.23687 | 1.09134E-06 | 5.962039927 |
| GO POSITIVE REGULATION OF NERVOUS SYSTEM DEVELOPMENT | 24 | 5.96675 | 1.38162E-06 | 5.859611389 |
| GO RESPONSE TO UV | 13 | 1.55754 | 1.39115E-06 | 5.85662604 |
| GO MACROMOLECULE CATABOLIC PROCESS | 41 | 15.5181 | 1.40686E-06 | 5.851749118 |
| GO REGULATION OF MICROGLIAL CELL ACTIVATION | 7 | 0.22905 | 1.43344E-06 | 5.843620481 |
| GO COGNITION | 18 | 3.35558 | 1.60219E-06 | 5.795285983 |
| GO NEGATIVE REGULATION OF DEFENSE RESPONSE | 15 | 2.23324 | 1.60952E-06 | 5.793303622 |
| GO MULTICELLULAR ORGANISM AGING | 8 | 0.389385 | 1.79932E-06 | 5.744891593 |
| GO RESPONSE TO KETONE | 15 | 2.25614 | 1.81352E-06 | 5.741477651 |
| GO TELOMERIC LOOP DISASSEMBLY | 6 | 0.125977 | 1.94071E-06 | 5.712039356 |
| GO TOR SIGNALING | 12 | 1.31704 | 1.99575E-06 | 5.699893862 |
| GO POSITIVE REGULATION OF CHROMOSOME ORGANIZATION | 14 | 1.93547 | 2.00663E-06 | 5.697532699 |
| GO NEGATIVE REGULATION OF CATALYTIC ACTIVITY | 29 | 8.68099 | 2.02649E-06 | 5.693255535 |
| GO REGULATION OF CELLULAR COMPONENT MOVEMENT | 33 | 10.9142 | 2.02925E-06 | 5.692664445 |

| | | | | |
|---|---|---|---|---|
| GO SIGNAL TRANSDUCTION BY P53 CLASS MEDIATOR | 17 | 3.02346 | 2.04323E-06 | 5.689682743 |
| GO NEGATIVE REGULATION OF TELOMERE MAINTENANCE | 8 | 0.400837 | 2.11481E-06 | 5.674728645 |
| GO POSITIVE REGULATION OF PROTEIN BINDING | 11 | 1.05363 | 2.11481E-06 | 5.674728645 |
| GO NEGATIVE REGULATION OF TRANSFERASE ACTIVITY | 17 | 3.04636 | 2.24187E-06 | 5.649389575 |
| GO POSITIVE REGULATION OF CELL POPULATION PROLIFERATION | 32 | 10.3989 | 2.24553E-06 | 5.648681138 |
| GO RNA DEPENDENT DNA BIOSYNTHETIC PROCESS | 10 | 0.813127 | 2.24575E-06 | 5.648638592 |
| GO APOPTOTIC MITOCHONDRIAL CHANGES | 12 | 1.33994 | 2.2915E-06 | 5.639880138 |
| GO REGULATION OF TRANSMEMBRANE TRANSPORT | 24 | 6.16144 | 2.29373E-06 | 5.639457705 |
| GO AUTOPHAGY OF MITOCHONDRION | 10 | 0.82458 | 2.50442E-06 | 5.601292837 |
| GO DNA REPLICATION | 17 | 3.09217 | 2.69276E-06 | 5.569802353 |
| GO CELLULAR RESPONSE TO X RAY | 6 | 0.13743 | 2.72458E-06 | 5.564700436 |
| GO ORGANELLE DISASSEMBLY | 11 | 1.08799 | 2.76696E-06 | 5.557997119 |
| GO GLIAL CELL DIFFERENTIATION | 15 | 2.34776 | 2.77873E-06 | 5.55615365 |
| GO REGULATION OF MITOCHONDRIAL FISSION | 7 | 0.263407 | 2.92556E-06 | 5.533790991 |
| GO NEGATIVE REGULATION OF RNA BIOSYNTHETIC PROCESS | 38 | 14.1324 | 3.00952E-06 | 5.521502766 |
| GO POSITIVE REGULATION OF RESPONSE TO DNA DAMAGE STIMULUS | 11 | 1.09944 | 3.00952E-06 | 5.521502766 |
| GO REGULATION OF PROTEASOMAL UBIQUITIN DEPENDENT PROTEIN CATABOLIC PROCESS | 12 | 1.38575 | 3.11314E-06 | 5.506801348 |
| GO REGULATION OF INTRACELLULAR TRANSPORT | 21 | 4.85586 | 3.20477E-06 | 5.494203133 |
| GO GLIOGENESIS | 17 | 3.14944 | 3.35364E-06 | 5.474483559 |
| GO ASTROCYTE DIFFERENTIATION | 10 | 0.858937 | 3.41723E-06 | 5.46632579 |
| GO POSITIVE REGULATION OF CELL AGING | 6 | 0.148882 | 3.80464E-06 | 5.41968643 |
| GO RESPONSE TO XENOBIOTIC STIMULUS | 17 | 3.18379 | 3.85024E-06 | 5.414512198 |
| GO REGULATION OF SYNAPTIC PLASTICITY | 14 | 2.0729 | 4.07976E-06 | 5.389365384 |
| GO NEGATIVE REGULATION OF CELL CYCLE | 25 | 6.90586 | 4.28115E-06 | 5.368439555 |
| GO REGULATION OF ANATOMICAL STRUCTURE MORPHOGENESIS | 34 | 11.9221 | 4.36967E-06 | 5.35955136 |
| GO CELLULAR RESPONSE TO OXYGEN RADICAL | 7 | 0.286312 | 4.54631E-06 | 5.342340954 |
| GO CELLULAR RESPONSE TO ABIOTIC STIMULUS | 18 | 3.64189 | 4.58156E-06 | 5.338986622 |
| GO REGENERATION | 14 | 2.11871 | 5.16977E-06 | 5.286528778 |
| GO LYSOSOMAL TRANSPORT | 11 | 1.17961 | 5.56996E-06 | 5.254147924 |
| GO NEGATIVE REGULATION OF RESPONSE TO DNA DAMAGE STIMULUS | 10 | 0.9162 | 5.74237E-06 | 5.240908828 |
| GO PROTEIN REPAIR | 5 | 0.068715 | 6.03597E-06 | 5.219252927 |
| GO REGULATION OF DNA BIOSYNTHETIC PROCESS | 11 | 1.19106 | 6.03597E-06 | 5.219252927 |
| GO SECRETION | 44 | 18.5416 | 6.93244E-06 | 5.159113881 |
| GO NEGATIVE REGULATION OF INFLAMMATORY RESPONSE | 12 | 1.51173 | 7.10471E-06 | 5.148453644 |
| GO REGULATION OF TELOMERE MAINTENANCE VIA TELOMERE LENGTHENING | 9 | 0.698602 | 7.14149E-06 | 5.146211168 |
| GO POSITIVE REGULATION OF TRANSCRIPTION BY RNA POLYMERASE II | 36 | 13.4567 | 7.62275E-06 | 5.117888323 |
| GO REGULATION OF PROTEIN OLIGOMERIZATION | 8 | 0.492457 | 7.62275E-06 | 5.117888323 |
| GO PROTEIN LOCALIZATION TO CHROMOSOME | 10 | 0.950557 | 7.66307E-06 | 5.115597207 |
| GO REGULATION OF SMALL MOLECULE METABOLIC PROCESS | 18 | 3.79078 | 0.000007786 | 5.108685601 |
| GO PROTEIN LOCALIZATION TO CHROMOSOME TELOMERIC REGION | 7 | 0.32067 | 0.000008414 | 5.074997492 |
| GO CELLULAR MACROMOLECULE CATABOLIC PROCESS | 35 | 12.9184 | 8.64686E-06 | 5.063141573 |
| GO REGULATION OF LIPID LOCALIZATION | 12 | 1.54609 | 8.68436E-06 | 5.061262182 |
| GO NEGATIVE REGULATION OF RESPONSE TO OXIDATIVE STRESS | 8 | 0.50391 | 8.73074E-06 | 5.058948945 |
| GO POSITIVE REGULATION OF TRANSMEMBRANE TRANSPORT | 14 | 2.23324 | 9.01238E-06 | 5.045160505 |
| GO CENTRAL NERVOUS SYSTEM DEVELOPMENT | 32 | 11.1433 | 9.06673E-06 | 5.042549317 |

| | | | | |
|---|---|---|---|---|
| GO NEUROGENESIS | 43 | 18.0835 | 9.08024E-06 | 5.041902672 |
| GO PROTEIN HOMOOLIGOMERIZATION | 18 | 3.84804 | 9.37981E-06 | 5.027805959 |
| GO MODULATION OF AGE RELATED BEHAVIORAL DECLINE | 5 | 0.0801675 | 9.70176E-06 | 5.013149473 |
| GO CELLULAR RESPONSE TO EXTRACELLULAR STIMULUS | 16 | 3.02346 | 9.87946E-06 | 5.005266793 |
| GO RESPONSE TO CORTICOSTEROID | 13 | 1.90111 | 9.87946E-06 | 5.005266793 |
| GO MUSCLE CELL DIFFERENTIATION | 18 | 3.87094 | 1.00705E-05 | 4.996948966 |
| GO RESPONSE TO INSULIN | 16 | 3.03491 | 1.02957E-05 | 4.987344121 |
| GO RESPONSE TO ALCOHOL | 15 | 2.64553 | 1.05338E-05 | 4.977414932 |
| GO MUSCLE TISSUE DEVELOPMENT | 19 | 4.3405 | 1.08373E-05 | 4.965078904 |
| GO COVALENT CHROMATIN MODIFICATION | 21 | 5.2796 | 1.08699E-05 | 4.963774451 |
| GO MUSCLE CELL APOPTOTIC PROCESS | 9 | 0.744412 | 1.08972E-05 | 4.962685078 |
| GO REGULATION OF MEMBRANE POTENTIAL | 20 | 4.8215 | 1.14209E-05 | 4.942299671 |
| GO POSITIVE REGULATION OF SYNAPTIC TRANSMISSION | 13 | 1.93547 | 0.000011691 | 4.932148339 |
| GO NEGATIVE REGULATION OF CHROMOSOME ORGANIZATION | 12 | 1.60335 | 1.18727E-05 | 4.925450506 |
| GO DETERMINATION OF ADULT LIFESPAN | 6 | 0.194692 | 1.19732E-05 | 4.921789763 |
| GO REGULATION OF INTRACELLULAR PROTEIN TRANSPORT | 15 | 2.69134 | 1.26617E-05 | 4.897507981 |
| GO REGULATION OF RELEASE OF CYTOCHROME C FROM MITOCHONDRIA | 8 | 0.538267 | 1.29127E-05 | 4.888982939 |
| GO CELLULAR CHEMICAL HOMEOSTASIS | 28 | 9.04747 | 1.33501E-05 | 4.874515481 |
| GO NEGATIVE REGULATION OF CELLULAR AMIDE METABOLIC PROCESS | 12 | 1.62625 | 1.34865E-05 | 4.870100743 |
| GO RESPONSE TO STEROID HORMONE | 19 | 4.42066 | 1.37047E-05 | 4.863130467 |
| GO AMYLOID BETA CLEARANCE | 7 | 0.355027 | 0.000014122 | 4.850103793 |
| GO CALMODULIN DEPENDENT KINASE SIGNALING PATHWAY | 5 | 0.09162 | 1.47153E-05 | 4.83223088 |
| GO REGULATION OF MITOCHONDRIAL MEMBRANE POTENTIAL | 9 | 0.77877 | 1.48815E-05 | 4.827353291 |
| GO CELL CYCLE ARREST | 15 | 2.73715 | 1.51671E-05 | 4.81909745 |
| GO GLUCOSE METABOLIC PROCESS | 11 | 1.33994 | 1.60671E-05 | 4.794062503 |
| GO RESPONSE TO WOUNDING | 25 | 7.48993 | 1.61841E-05 | 4.790911447 |
| GO REGULATION OF MULTICELLULAR ORGANISM GROWTH | 9 | 0.790222 | 1.64584E-05 | 4.783612387 |
| GO REGULATION OF RESPONSE TO DRUG | 10 | 1.05363 | 1.65508E-05 | 4.781181009 |
| GO REGULATION OF SUPEROXIDE METABOLIC PROCESS | 7 | 0.36648 | 1.66393E-05 | 4.778864948 |
| GO PEPTIDYL TYROSINE MODIFICATION | 18 | 4.05418 | 1.79256E-05 | 4.746526299 |
| GO SYNAPTIC SIGNALING | 26 | 8.08546 | 1.80031E-05 | 4.744652706 |
| GO REGULATION OF UBIQUITIN DEPENDENT PROTEIN CATABOLIC PROCESS | 12 | 1.68352 | 1.83647E-05 | 4.736016162 |
| GO REGULATION OF SIGNAL TRANSDUCTION BY P53 CLASS MEDIATOR | 13 | 2.03854 | 1.92418E-05 | 4.715754304 |
| GO MITOCHONDRIAL FISSION | 7 | 0.377932 | 1.93968E-05 | 4.712269912 |
| GO MULTICELLULAR ORGANISM GROWTH | 12 | 1.69497 | 1.93968E-05 | 4.712269912 |
| GO NEGATIVE REGULATION OF DNA REPAIR | 7 | 0.377932 | 1.93968E-05 | 4.712269912 |
| GO NEGATIVE REGULATION OF INTRINSIC APOPTOTIC SIGNALING PATHWAY | 10 | 1.07653 | 1.93968E-05 | 4.712269912 |
| GO REGULATION OF OXIDOREDUCTASE ACTIVITY | 10 | 1.07653 | 1.93968E-05 | 4.712269912 |
| GO REGULATION OF RESPONSE TO REACTIVE OXYGEN SPECIES | 7 | 0.377932 | 1.93968E-05 | 4.712269912 |
| GO POSITIVE REGULATION OF UBIQUITIN DEPENDENT PROTEIN CATABOLIC PROCESS | 10 | 1.08799 | 2.10076E-05 | 4.67762356 |
| GO REGULATION OF PROTEIN BINDING | 14 | 2.43938 | 2.18212E-05 | 4.66112137 |
| GO CELLULAR RESPONSE TO STARVATION | 12 | 1.71787 | 2.18295E-05 | 4.660956212 |
| GO NEGATIVE REGULATION OF RESPONSE TO EXTERNAL STIMULUS | 17 | 3.67625 | 2.18295E-05 | 4.660956212 |
| GO REGULATION OF SYSTEM PROCESS | 23 | 6.59664 | 2.32125E-05 | 4.634278083 |
| GO REGULATION OF NEUROTRANSMITTER LEVELS | 17 | 3.69916 | 2.35783E-05 | 4.627487511 |

| | | | | |
|---|---|---|---|---|
| GO POSITIVE REGULATION OF NEURON APOPTOTIC PROCESS | 8 | 0.59553 | 2.37181E-05 | 4.624920104 |
| GO POSITIVE REGULATION OF AUTOPHAGY OF MITOCHONDRION | 6 | 0.22905 | 2.41363E-05 | 4.617329305 |
| GO REGULATION OF GENE EXPRESSION EPIGENETIC | 17 | 3.71061 | 2.43607E-05 | 4.613310236 |
| GO MONOSACCHARIDE METABOLIC PROCESS | 12 | 1.74078 | 2.44486E-05 | 4.611746005 |
| GO ORGANONITROGEN COMPOUND CATABOLIC PROCESS | 36 | 14.2698 | 0.000025509 | 4.593306566 |
| GO REGULATION OF DNA BINDING | 11 | 1.42011 | 2.55625E-05 | 4.592396675 |
| GO REGULATION OF DOUBLE STRAND BREAK REPAIR | 9 | 0.847485 | 0.000026274 | 4.580473804 |
| GO POSITIVE REGULATION OF PROTEIN SERINE THREONINE KINASE ACTIVITY | 17 | 3.74497 | 2.71533E-05 | 4.566177382 |
| GO RESPONSE TO STARVATION | 13 | 2.14162 | 3.07299E-05 | 4.512438853 |
| GO NEGATIVE REGULATION OF DNA BIOSYNTHETIC PROCESS | 7 | 0.41229 | 3.11293E-05 | 4.506830645 |
| GO REGULATION OF MAP KINASE ACTIVITY | 17 | 3.79078 | 3.16416E-05 | 4.499741564 |
| GO ESTABLISHMENT OF PROTEIN LOCALIZATION TO ORGANELLE | 22 | 6.21871 | 3.22639E-05 | 4.491283137 |
| GO NEGATIVE REGULATION OF PROTEOLYSIS | 17 | 3.81368 | 3.40805E-05 | 4.467494042 |
| GO ORGANELLE FISSION | 20 | 5.24524 | 3.56626E-05 | 4.447786998 |
| GO RESPONSE TO CARBOHYDRATE | 14 | 2.56536 | 3.66628E-05 | 4.43577437 |
| GO REGULATION OF MACROPHAGE ACTIVATION | 8 | 0.64134 | 3.78527E-05 | 4.421903137 |
| GO REGULATION OF TELOMERE MAINTENANCE | 9 | 0.893295 | 3.83535E-05 | 4.416194998 |
| GO REGULATION OF PEPTIDASE ACTIVITY | 19 | 4.78714 | 3.85199E-05 | 4.414314849 |
| GO CARBOHYDRATE METABOLIC PROCESS | 22 | 6.29887 | 3.89042E-05 | 4.410003511 |
| GO NEGATIVE REGULATION OF LONG TERM SYNAPTIC POTENTIATION | 5 | 0.125977 | 4.28298E-05 | 4.368253954 |
| GO REGULATION OF ASPARTIC TYPE PEPTIDASE ACTIVITY | 5 | 0.125977 | 4.28298E-05 | 4.368253954 |
| GO PROTEIN CATABOLIC PROCESS | 29 | 10.2729 | 4.32886E-05 | 4.36362646 |
| GO REGULATION OF SYNAPSE STRUCTURE OR ACTIVITY | 14 | 2.61117 | 4.38249E-05 | 4.358279066 |
| GO INCLUSION BODY ASSEMBLY | 6 | 0.263407 | 0.000045444 | 4.342523449 |
| GO REGULATION OF CHOLESTEROL EFFLUX | 6 | 0.263407 | 0.000045444 | 4.342523449 |
| GO REGULATION OF CARBOHYDRATE METABOLIC PROCESS | 13 | 2.23324 | 0.000045835 | 4.338802764 |
| GO CELLULAR MACROMOLECULE LOCALIZATION | 46 | 21.4734 | 4.62639E-05 | 4.334757759 |
| GO LIPID LOCALIZATION | 18 | 4.39776 | 4.91068E-05 | 4.308858365 |
| GO CELLULAR RESPONSE TO HEAT | 11 | 1.53463 | 4.91764E-05 | 4.308243267 |
| GO POSITIVE REGULATION OF CELLULAR COMPONENT BIOGENESIS | 21 | 5.88658 | 4.96779E-05 | 4.303836771 |
| GO NEGATIVE REGULATION OF CELL AGING | 6 | 0.27486 | 5.53863E-05 | 4.256597646 |
| GO INSULIN RECEPTOR SIGNALING PATHWAY | 11 | 1.55754 | 5.57721E-05 | 4.253583003 |
| GO VACUOLAR TRANSPORT | 11 | 1.55754 | 5.57721E-05 | 4.253583003 |
| GO POSITIVE REGULATION OF DEVELOPMENTAL GROWTH | 12 | 1.91257 | 5.75219E-05 | 4.240166777 |
| GO REGULATION OF MUSCLE ADAPTATION | 9 | 0.950557 | 5.89734E-05 | 4.229343833 |
| GO CELLULAR DETOXIFICATION | 10 | 1.24832 | 6.03476E-05 | 4.219339997 |
| GO POSITIVE REGULATION OF ESTABLISHMENT OF PROTEIN LOCALIZATION | 19 | 4.95893 | 6.06917E-05 | 4.216870698 |
| GO RESPONSE TO ENDOPLASMIC RETICULUM STRESS | 15 | 3.11508 | 6.07686E-05 | 4.216320769 |
| GO REGULATION OF CELLULAR RESPONSE TO DRUG | 6 | 0.286312 | 6.66368E-05 | 4.176285867 |
| GO REGULATION OF PROTEIN LOCALIZATION TO NUCLEUS | 10 | 1.27123 | 6.96457E-05 | 4.157105692 |
| GO CELLULAR RESPONSE TO CARBOHYDRATE STIMULUS | 11 | 1.60335 | 7.12951E-05 | 4.146940317 |
| GO SMALL MOLECULE METABOLIC PROCESS | 42 | 19.057 | 7.32024E-05 | 4.13547468 |
| GO RHYTHMIC PROCESS | 15 | 3.17234 | 7.44406E-05 | 4.128190135 |
| GO LONG TERM SYNAPTIC POTENTIATION | 9 | 0.984915 | 7.54976E-05 | 4.122066854 |
| GO POSITIVE REGULATION OF CELLULAR AMIDE METABOLIC PROCESS | 11 | 1.6148 | 7.54976E-05 | 4.122066854 |

| | | | | |
|---|---|---|---|---|
| GO NEGATIVE REGULATION OF TELOMERE MAINTENANCE VIA TELOMERE LENGTHENING | 6 | 0.297765 | 0.00007981 | 4.097942689 |
| GO POSITIVE REGULATION OF CELL CYCLE PROCESS | 15 | 3.19525 | 8.02187E-05 | 4.09572438 |
| GO PROTEIN KINASE B SIGNALING | 14 | 2.7715 | 8.02187E-05 | 4.09572438 |
| GO REGULATION OF LONG TERM SYNAPTIC POTENTIATION | 7 | 0.492457 | 8.14187E-05 | 4.089275836 |
| GO IMPORT INTO CELL | 24 | 7.74189 | 8.37432E-05 | 4.077050448 |
| GO REGULATION OF CELLULAR CARBOHYDRATE METABOLIC PROCESS | 10 | 1.30558 | 8.44014E-05 | 4.073650349 |
| GO REGULATION OF GENERATION OF PRECURSOR METABOLITES AND ENERGY | 11 | 1.63771 | 8.44014E-05 | 4.073650349 |
| GO REGULATION OF GLIOGENESIS | 10 | 1.30558 | 8.44014E-05 | 4.073650349 |
| GO NEGATIVE REGULATION OF INTRACELLULAR TRANSPORT | 8 | 0.73296 | 8.58212E-05 | 4.066405417 |
| GO REGULATION OF PROTEIN TYROSINE KINASE ACTIVITY | 9 | 1.00782 | 8.78301E-05 | 4.056356623 |
| GO MEMORY | 10 | 1.31704 | 9.01725E-05 | 4.04492589 |
| GO CELLULAR RESPONSE TO INSULIN STIMULUS | 13 | 2.40502 | 9.26077E-05 | 4.033352902 |
| GO NEGATIVE REGULATION OF REACTIVE OXYGEN SPECIES METABOLIC PROCESS | 6 | 0.309217 | 9.37924E-05 | 4.027832351 |
| GO PROTEIN LOCALIZATION TO NUCLEUS | 14 | 2.82877 | 9.80647E-05 | 4.008487296 |
| GO NEGATIVE REGULATION OF CELL DEVELOPMENT | 16 | 3.71061 | 9.91568E-05 | 4.003677497 |
| GO CELLULAR RESPONSE TO MANGANESE ION | 5 | 0.160335 | 9.98878E-05 | 4.000487552 |
| GO REGULATION OF ATP BIOSYNTHETIC PROCESS | 5 | 0.160335 | 9.98878E-05 | 4.000487552 |
| GO REGULATION OF SIGNALING RECEPTOR ACTIVITY | 22 | 6.73407 | 0.00010055 | 3.997617925 |
| GO REGULATION OF NEURON DIFFERENTIATION | 23 | 7.29524 | 0.000103246 | 3.986126765 |
| GO LIPID METABOLIC PROCESS | 34 | 13.972 | 0.000104964 | 3.978959627 |
| GO MITOCHONDRIAL TRANSPORT | 14 | 2.85167 | 0.000104964 | 3.978959627 |
| GO MYELOID LEUKOCYTE ACTIVATION | 23 | 7.30669 | 0.000104964 | 3.978959627 |
| GO POSITIVE REGULATION OF PEPTIDASE ACTIVITY | 12 | 2.05 | 0.000104964 | 3.978959627 |
| GO CELLULAR RESPONSE TO LIPID | 22 | 6.76843 | 0.000107358 | 3.969165588 |
| GO CELLULAR RESPONSE TO COPPER ION | 6 | 0.32067 | 0.000109343 | 3.961209015 |
| GO REGULATION OF CELLULAR RESPIRATION | 6 | 0.32067 | 0.000109343 | 3.961209015 |
| GO LOCOMOTION | 43 | 20.1335 | 0.00011077 | 3.955577844 |
| GO POSITIVE REGULATION OF MACROAUTOPHAGY | 8 | 0.767317 | 0.000112233 | 3.949879428 |
| GO CELL ACTIVATION | 36 | 15.3578 | 0.000114165 | 3.942467019 |
| GO AMYLOID FIBRIL FORMATION | 5 | 0.171787 | 0.000127503 | 3.894479597 |
| GO FORMATION OF EXTRACHROMOSOMAL CIRCULAR DNA | 5 | 0.171787 | 0.000127503 | 3.894479597 |
| GO REGULATION OF LIPID METABOLIC PROCESS | 17 | 4.27178 | 0.000127503 | 3.894479597 |
| GO POSITIVE REGULATION OF GROWTH | 14 | 2.90893 | 0.000127815 | 3.893418176 |
| GO CELLULAR RESPONSE TO EXTERNAL STIMULUS | 16 | 3.80223 | 0.000128017 | 3.892732354 |
| GO SIGNAL RELEASE | 19 | 5.26815 | 0.000128428 | 3.891340281 |
| GO POSITIVE REGULATION OF MAP KINASE ACTIVITY | 14 | 2.92039 | 0.00013257 | 3.877554744 |
| GO PROTEASOMAL PROTEIN CATABOLIC PROCESS | 19 | 5.29105 | 0.000135838 | 3.866978721 |
| GO CELL MOTILITY | 39 | 17.5681 | 0.000143628 | 3.842760887 |
| GO NEGATIVE REGULATION OF TRANSPORT | 19 | 5.31396 | 0.000143628 | 3.842760887 |
| GO REGULATION OF HYDROLASE ACTIVITY | 34 | 14.224 | 0.000146847 | 3.833134921 |
| GO POSITIVE REGULATION OF MUSCLE TISSUE DEVELOPMENT | 8 | 0.801675 | 0.000146995 | 3.832697437 |
| GO MITOTIC CELL CYCLE | 29 | 11.0517 | 0.000151265 | 3.820261548 |
| GO REGULATION OF CELL PROJECTION ORGANIZATION | 23 | 7.50139 | 0.000153044 | 3.815183692 |
| GO DETOXIFICATION | 10 | 1.42011 | 0.000156945 | 3.804252516 |
| GO POSITIVE REGULATION OF CHOLESTEROL EFFLUX | 5 | 0.18324 | 0.000160707 | 3.793965206 |

| | | | | |
|---|---|---|---|---|
| GO STRIATED MUSCLE ADAPTATION | 7 | 0.561172 | 0.000161503 | 3.791819406 |
| GO POSITIVE REGULATION OF RESPONSE TO EXTERNAL STIMULUS | 15 | 3.43575 | 0.00016866 | 3.772987904 |
| GO MUSCLE ORGAN DEVELOPMENT | 17 | 4.38631 | 0.000172213 | 3.763934068 |
| GO POSITIVE REGULATION OF CELL CYCLE | 17 | 4.38631 | 0.000172213 | 3.763934068 |
| GO SYNAPTIC TRANSMISSION DOPAMINERGIC | 6 | 0.355027 | 0.000173839 | 3.759852785 |
| GO EPITHELIAL CELL APOPTOTIC PROCESS | 9 | 1.12234 | 0.000181488 | 3.741152085 |
| GO POSITIVE REGULATION OF PROTEIN LOCALIZATION TO NUCLEUS | 8 | 0.836032 | 0.000191054 | 3.718843865 |
| GO POSITIVE REGULATION OF SMALL MOLECULE METABOLIC PROCESS | 9 | 1.1338 | 0.000194753 | 3.710515844 |
| GO REGULATION OF CIRCADIAN RHYTHM | 9 | 1.1338 | 0.000194753 | 3.710515844 |
| GO VACUOLE ORGANIZATION | 11 | 1.82095 | 0.000197381 | 3.704694655 |
| GO POSITIVE REGULATION OF NEUROINFLAMMATORY RESPONSE | 5 | 0.194692 | 0.000201046 | 3.696704563 |
| GO PEPTIDYL SERINE MODIFICATION | 15 | 3.51592 | 0.000215021 | 3.667519123 |
| GO REGULATION OF MUSCLE SYSTEM PROCESS | 13 | 2.63407 | 0.000215021 | 3.667519123 |
| GO POSITIVE REGULATION OF NEURON PROJECTION DEVELOPMENT | 14 | 3.06927 | 0.000217605 | 3.66233113 |
| GO NEGATIVE REGULATION OF EXTRINSIC APOPTOTIC SIGNALING PATHWAY | 9 | 1.1567 | 0.000223253 | 3.651202696 |
| GO REGULATION OF TRANSCRIPTION REGULATORY REGION DNA BINDING | 7 | 0.59553 | 0.000223253 | 3.651202696 |
| GO REGULATION OF EPITHELIAL CELL APOPTOTIC PROCESS | 8 | 0.858937 | 0.000224772 | 3.64825779 |
| GO CIRCULATORY SYSTEM PROCESS | 20 | 6.04692 | 0.000228504 | 3.641106193 |
| GO LOCOMOTORY BEHAVIOR | 12 | 2.24469 | 0.000230908 | 3.63656102 |
| GO NEGATIVE REGULATION OF MUSCLE CELL APOPTOTIC PROCESS | 6 | 0.377932 | 0.000231611 | 3.635240818 |
| GO POSITIVE REGULATION OF LOCOMOTION | 20 | 6.05837 | 0.000233262 | 3.632156005 |
| GO SYNAPTIC VESICLE LOCALIZATION | 11 | 1.86676 | 0.000240553 | 3.618789223 |
| GO NOTCH RECEPTOR PROCESSING LIGAND DEPENDENT | 4 | 0.0801675 | 0.000242538 | 3.615220208 |
| GO ESTABLISHMENT OF PROTEIN LOCALIZATION TO TELOMERE | 5 | 0.206145 | 0.000246355 | 3.608438619 |
| GO NEGATIVE REGULATION OF CELLULAR SENESCENCE | 5 | 0.206145 | 0.000246355 | 3.608438619 |
| GO NEGATIVE REGULATION OF RELEASE OF CYTOCHROME C FROM MITOCHONDRIA | 5 | 0.206145 | 0.000246355 | 3.608438619 |
| GO REACTIVE OXYGEN SPECIES BIOSYNTHETIC PROCESS | 9 | 1.17961 | 0.00025297 | 3.596930979 |
| GO CELLULAR COMPONENT DISASSEMBLY | 20 | 6.10418 | 0.000255937 | 3.591866925 |
| GO CYTOKINE PRODUCTION | 24 | 8.41759 | 0.000285582 | 3.544269169 |
| GO REGULATION OF LIPID TRANSPORT | 9 | 1.20251 | 0.000290781 | 3.536433974 |
| GO NEGATIVE REGULATION OF KINASE ACTIVITY | 13 | 2.72569 | 0.000293688 | 3.532113798 |
| GO REGULATION OF MUSCLE ORGAN DEVELOPMENT | 10 | 1.54609 | 0.0002962 | 3.528414946 |
| GO POSITIVE REGULATION OF OXIDOREDUCTASE ACTIVITY | 7 | 0.629887 | 0.000301494 | 3.520721326 |
| GO NEGATIVE REGULATION OF TELOMERE MAINTENANCE VIA TELOMERASE | 5 | 0.217597 | 0.000303132 | 3.518368215 |
| GO CARDIOVASCULAR SYSTEM DEVELOPMENT | 23 | 7.87932 | 0.000308418 | 3.510860283 |
| GO VESICLE MEDIATED TRANSPORT IN SYNAPSE | 12 | 2.32486 | 0.000312399 | 3.505290365 |
| GO SYNAPSE ORGANIZATION | 17 | 4.62681 | 0.000315716 | 3.500703408 |
| GO IMMUNE SYSTEM DEVELOPMENT | 28 | 10.9257 | 0.000320249 | 3.494512218 |
| GO PROTEIN STABILIZATION | 11 | 1.93547 | 0.000321024 | 3.493462498 |
| GO REGULATION OF PROTEIN TARGETING | 8 | 0.9162 | 0.000332269 | 3.478510176 |
| GO RESPONSE TO GROWTH FACTOR | 23 | 7.93658 | 0.0003413 | 3.466863712 |
| GO NEURON INTRINSIC APOPTOTIC SIGNALING PATHWAY IN RESPONSE TO OXIDATIVE STRESS | 4 | 0.09162 | 0.000343547 | 3.46401384 |
| GO PEPTIDYL LYSINE DEACETYLATION | 4 | 0.09162 | 0.000343547 | 3.46401384 |
| GO REGULATION OF AMYLOID FIBRIL FORMATION | 4 | 0.09162 | 0.000343547 | 3.46401384 |
| GO REGULATION OF ASTROCYTE ACTIVATION | 4 | 0.09162 | 0.000343547 | 3.46401384 |

| | | | | |
|---|---|---|---|---|
| GO REGULATION OF NUCLEOTIDE BIOSYNTHETIC PROCESS | 6 | 0.41229 | 0.000343547 | 3.46401384 |
| GO REGULATION OF HISTONE MODIFICATION | 10 | 1.58044 | 0.000344359 | 3.462988562 |
| GO RESPONSE TO ALKALOID | 9 | 1.23687 | 0.000346556 | 3.460226578 |
| GO POSITIVE REGULATION OF PROTEIN COMPLEX ASSEMBLY | 13 | 2.78296 | 0.000350065 | 3.455851308 |
| GO CELLULAR PROTEIN CATABOLIC PROCESS | 24 | 8.56647 | 0.000361756 | 3.441584257 |
| GO REGULATION OF CELL CYCLE PROCESS | 24 | 8.56647 | 0.000361756 | 3.441584257 |
| GO RESPONSE TO MITOCHONDRIAL DEPOLARISATION | 5 | 0.22905 | 0.000361756 | 3.441584257 |
| GO TEMPERATURE HOMEOSTASIS | 11 | 1.96983 | 0.000365103 | 3.437584598 |
| GO NEGATIVE REGULATION OF CELLULAR PROTEIN LOCALIZATION | 9 | 1.24832 | 0.00036702 | 3.435310269 |
| GO EPITHELIAL CELL PROLIFERATION | 16 | 4.21452 | 0.000387034 | 3.412250882 |
| GO ORGANONITROGEN COMPOUND BIOSYNTHETIC PROCESS | 41 | 19.8701 | 0.000388096 | 3.411060833 |
| GO REGULATION OF MUSCLE HYPERTROPHY | 7 | 0.664245 | 0.000394711 | 3.40372077 |
| GO POSITIVE REGULATION OF CELL PROJECTION ORGANIZATION | 16 | 4.22597 | 0.000397404 | 3.400767766 |
| GO ACTIVATION OF PROTEIN KINASE ACTIVITY | 15 | 3.75642 | 0.000417036 | 3.379826454 |
| GO EXTRACELLULAR STRUCTURE ORGANIZATION | 17 | 4.76424 | 0.000434862 | 3.361648541 |
| GO RESPONSE TO MANGANESE ION | 5 | 0.240502 | 0.000435779 | 3.360733702 |
| GO HISTONE PHOSPHORYLATION | 6 | 0.435195 | 0.000437225 | 3.359295014 |
| GO RESPONSE TO CATECHOLAMINE | 6 | 0.435195 | 0.000437225 | 3.359295014 |
| GO NEGATIVE REGULATION OF ESTABLISHMENT OF PROTEIN LOCALIZATION | 11 | 2.01564 | 0.000438428 | 3.358101718 |
| GO NEURON PROJECTION ORGANIZATION | 8 | 0.96201 | 0.000440001 | 3.356546336 |
| GO REGULATION OF DNA BINDING TRANSCRIPTION FACTOR ACTIVITY | 17 | 4.77569 | 0.000442792 | 3.353800234 |
| GO REGULATION OF VESICLE MEDIATED TRANSPORT | 19 | 5.85223 | 0.00046369 | 3.33377227 |
| GO DNA STRAND RENATURATION | 4 | 0.103072 | 0.000469649 | 3.328226598 |
| GO REGULATION OF ASPARTIC TYPE ENDOPEPTIDASE ACTIVITY INVOLVED IN AMYLOID PRECURSOR PROTEIN CATABOLIC PROCESS | 4 | 0.103072 | 0.000469649 | 3.328226598 |
| GO LYTIC VACUOLE ORGANIZATION | 7 | 0.68715 | 0.000471184 | 3.326809465 |
| GO POSITIVE REGULATION OF SMOOTH MUSCLE CELL PROLIFERATION | 8 | 0.973462 | 0.000471184 | 3.326809465 |
| GO REGULATION OF CHROMATIN ORGANIZATION | 11 | 2.03854 | 0.000477227 | 3.321274993 |
| GO NEGATIVE REGULATION OF GROWTH | 13 | 2.88603 | 0.000483372 | 3.31571851 |
| GO REGULATION OF AUTOPHAGY OF MITOCHONDRION | 6 | 0.446647 | 0.000489077 | 3.31062276 |
| GO NEGATIVE REGULATION OF TRANSCRIPTION BY RNA POLYMERASE II | 25 | 9.36814 | 0.000496406 | 3.304162978 |
| GO POSITIVE REGULATION OF DNA BINDING | 7 | 0.698602 | 0.00051354 | 3.289425723 |
| GO POSITIVE REGULATION OF STEROL TRANSPORT | 5 | 0.251955 | 0.00051354 | 3.289425723 |
| GO REGULATION OF PROTEIN MODIFICATION BY SMALL PROTEIN CONJUGATION OR REMOVAL | 12 | 2.47374 | 0.000519953 | 3.284035912 |
| GO MUSCLE HYPERTROPHY | 8 | 0.996367 | 0.000543095 | 3.265124196 |
| GO NEURON DIFFERENTIATION | 34 | 15.2776 | 0.00054356 | 3.26475251 |
| GO REGULATION OF SUPRAMOLECULAR FIBER ORGANIZATION | 15 | 3.85949 | 0.00054356 | 3.26475251 |
| GO MULTICELLULAR ORGANISMAL HOMEOSTASIS | 18 | 5.39413 | 0.000544099 | 3.264322072 |
| GO REGULATION OF SECRETION | 24 | 8.87569 | 0.000600672 | 3.221362612 |
| GO POSITIVE REGULATION OF INTRACELLULAR PROTEIN TRANSPORT | 10 | 1.70642 | 0.00060147 | 3.220786029 |
| GO GLUCOSE IMPORT | 7 | 0.721507 | 0.000611048 | 3.213924673 |
| GO POSITIVE REGULATION OF CARBOHYDRATE METABOLIC PROCESS | 7 | 0.721507 | 0.000611048 | 3.213924673 |
| GO RESPONSE TO CADMIUM ION | 7 | 0.721507 | 0.000611048 | 3.213924673 |
| GO CHOLESTEROL EFFLUX | 6 | 0.469552 | 0.00061595 | 3.21045454 |
| GO NOTCH RECEPTOR PROCESSING | 4 | 0.114525 | 0.000627083 | 3.202674973 |
| GO DENDRITE DEVELOPMENT | 12 | 2.531 | 0.000630556 | 3.200276337 |

| | | | | |
|---|---|---|---|---|
| GO NEGATIVE REGULATION OF NERVOUS SYSTEM DEVELOPMENT | 14 | 3.43575 | 0.000630556 | 3.200276337 |
| GO REGULATION OF WNT SIGNALING PATHWAY | 15 | 3.92821 | 0.000648735 | 3.187932671 |
| GO TISSUE REGENERATION | 7 | 0.73296 | 0.000663875 | 3.177913686 |
| GO POSITIVE REGULATION OF DNA RECOMBINATION | 6 | 0.481005 | 0.000688227 | 3.162268293 |
| GO RESPONSE TO DEXAMETHASONE | 6 | 0.481005 | 0.000688227 | 3.162268293 |
| GO REGULATION OF MITOTIC CELL CYCLE | 21 | 7.19217 | 0.000700238 | 3.154754325 |
| GO REGULATION OF NEURON PROJECTION DEVELOPMENT | 18 | 5.5201 | 0.000712281 | 3.14734864 |
| GO REGULATION OF CELL ACTIVATION | 19 | 6.06982 | 0.000713961 | 3.146325511 |
| GO POSITIVE REGULATION OF DNA REPAIR | 7 | 0.744412 | 0.000720267 | 3.142506483 |
| GO EXOCYTOSIS | 26 | 10.2385 | 0.00072301 | 3.140855696 |
| GO POSITIVE REGULATION OF GLUCOSE TRANSMEMBRANE TRANSPORT | 6 | 0.492457 | 0.000766881 | 3.115272022 |
| GO PROTEIN DESTABILIZATION | 6 | 0.492457 | 0.000766881 | 3.115272022 |
| GO LIPID STORAGE | 7 | 0.755865 | 0.000783264 | 3.106091834 |
| GO CELLULAR RESPONSE TO TOPOLOGICALLY INCORRECT PROTEIN | 10 | 1.77514 | 0.00079966 | 3.097094627 |
| GO NEUROTRANSMITTER TRANSPORT | 13 | 3.05782 | 0.000815261 | 3.088703333 |
| GO MITOCHONDRIAL DNA METABOLIC PROCESS | 4 | 0.125977 | 0.000818196 | 3.087142648 |
| GO NEGATIVE REGULATION OF TELOMERASE ACTIVITY | 4 | 0.125977 | 0.000818196 | 3.087142648 |
| GO POSITIVE REGULATION OF ATP BIOSYNTHETIC PROCESS | 4 | 0.125977 | 0.000818196 | 3.087142648 |
| GO REGULATION OF MULTI ORGANISM PROCESS | 16 | 4.53519 | 0.000831955 | 3.079900164 |
| GO DNA INTEGRITY CHECKPOINT | 10 | 1.78659 | 0.00083343 | 3.079130871 |
| GO POSITIVE REGULATION OF GLIOGENESIS | 7 | 0.767317 | 0.000844435 | 3.073433774 |
| GO CELLULAR RESPONSE TO KETONE | 8 | 1.07653 | 0.000859411 | 3.065799092 |
| GO REGULATION OF CARBOHYDRATE BIOSYNTHETIC PROCESS | 8 | 1.07653 | 0.000859411 | 3.065799092 |
| GO RESPONSE TO TOPOLOGICALLY INCORRECT PROTEIN | 11 | 2.21033 | 0.000901075 | 3.045239059 |
| GO POSTTRANSCRIPTIONAL REGULATION OF GENE EXPRESSION | 20 | 6.76843 | 0.000919261 | 3.036561165 |
| GO CIRCADIAN RHYTHM | 11 | 2.22178 | 0.000937654 | 3.027957389 |
| GO NEGATIVE REGULATION OF SMOOTH MUSCLE CELL PROLIFERATION | 6 | 0.515362 | 0.000937654 | 3.027957389 |
| GO PROTEIN ACETYLATION | 11 | 2.22178 | 0.000937654 | 3.027957389 |
| GO REGULATION OF LIPID STORAGE | 6 | 0.515362 | 0.000937654 | 3.027957389 |
| GO REGULATION OF STEROL TRANSPORT | 6 | 0.515362 | 0.000937654 | 3.027957389 |
| GO SUPRAMOLECULAR FIBER ORGANIZATION | 21 | 7.36396 | 0.000937654 | 3.027957389 |
| GO CELL DEATH IN RESPONSE TO HYDROGEN PEROXIDE | 5 | 0.297765 | 0.000955038 | 3.019979348 |
| GO NEGATIVE REGULATION OF RESPONSE TO DRUG | 5 | 0.297765 | 0.000955038 | 3.019979348 |
| GO POSITIVE REGULATION OF TRANSCRIPTION REGULATORY REGION DNA BINDING | 5 | 0.297765 | 0.000955038 | 3.019979348 |
| GO NON RECOMBINATIONAL REPAIR | 8 | 1.09944 | 0.000969087 | 3.013637232 |
| GO NEGATIVE REGULATION OF SYNAPTIC TRANSMISSION | 7 | 0.790222 | 0.000978044 | 3.009641607 |
| GO REGULATION OF DENDRITIC SPINE DEVELOPMENT | 7 | 0.790222 | 0.000978044 | 3.009641607 |
| GO REGULATION OF CALCIUM MEDIATED SIGNALING | 8 | 1.11089 | 0.00103343 | 2.985718935 |
| GO DOPAMINE BIOSYNTHETIC PROCESS | 4 | 0.13743 | 0.00103416 | 2.985412264 |
| GO MUSCLE ATROPHY | 4 | 0.13743 | 0.00103416 | 2.985412264 |
| GO NEGATIVE REGULATION OF BINDING | 10 | 1.84385 | 0.00103416 | 2.985412264 |
| GO NEURON DEVELOPMENT | 29 | 12.4489 | 0.00103416 | 2.985412264 |
| GO POSITIVE REGULATION OF MICROGLIAL CELL ACTIVATION | 4 | 0.13743 | 0.00103416 | 2.985412264 |
| GO ENERGY DERIVATION BY OXIDATION OF ORGANIC COMPOUNDS | 13 | 3.14944 | 0.00103956 | 2.98315044 |
| GO NEGATIVE REGULATION OF GENE EXPRESSION EPIGENETIC | 9 | 1.46592 | 0.00104978 | 2.978901705 |

| | | | | |
|---|---|---|---|---|
| GO POSITIVE REGULATION OF CHROMATIN ORGANIZATION | 8 | 1.12234 | 0.00109168 | 2.961904646 |
| GO ESTABLISHMENT OF PROTEIN LOCALIZATION TO CHROMOSOME | 5 | 0.309217 | 0.00109629 | 2.960074547 |
| GO PROTEIN MATURATION | 13 | 3.17234 | 0.00110949 | 2.954876608 |
| GO CELL SURFACE RECEPTOR SIGNALING PATHWAY INVOLVED IN CELL CELL SIGNALING | 20 | 6.8944 | 0.00112774 | 2.947791015 |
| GO HISTONE DEACETYLATION | 7 | 0.813127 | 0.00112774 | 2.947791015 |
| GO REGULATION OF ESTABLISHMENT OF PROTEIN LOCALIZATION TO MITOCHONDRION | 7 | 0.813127 | 0.00112774 | 2.947791015 |
| GO REGULATION OF PROTEIN ACETYLATION | 7 | 0.813127 | 0.00112774 | 2.947791015 |
| GO SELECTIVE AUTOPHAGY | 6 | 0.538267 | 0.00112774 | 2.947791015 |
| GO SIGNAL TRANSDUCTION IN RESPONSE TO DNA DAMAGE | 9 | 1.48882 | 0.00115945 | 2.935747975 |
| GO ORGANIC HYDROXY COMPOUND TRANSPORT | 12 | 2.73715 | 0.00119431 | 2.922882931 |
| GO REGULATION OF ION TRANSMEMBRANE TRANSPORT | 17 | 5.24524 | 0.00121156 | 2.916655073 |
| GO REGULATION OF DNA RECOMBINATION | 8 | 1.14525 | 0.00122648 | 2.911339529 |
| GO REGULATION OF TELOMERASE ACTIVITY | 6 | 0.54972 | 0.00124565 | 2.904603968 |
| GO NEGATIVE REGULATION OF MACROPHAGE DERIVED FOAM CELL DIFFERENTIATION | 4 | 0.148882 | 0.00130182 | 2.885449061 |
| GO POSITIVE REGULATION OF INSULIN LIKE GROWTH FACTOR RECEPTOR SIGNALING PATHWAY | 4 | 0.148882 | 0.00130182 | 2.885449061 |
| GO LIPID OXIDATION | 8 | 1.1567 | 0.00130203 | 2.885379009 |
| GO REGULATION OF GLUCOSE METABOLIC PROCESS | 7 | 0.836032 | 0.00130794 | 2.883412178 |
| GO REGULATION OF GLUCOSE TRANSMEMBRANE TRANSPORT | 7 | 0.836032 | 0.00130794 | 2.883412178 |
| GO REGULATION OF HORMONE LEVELS | 18 | 5.87513 | 0.00142118 | 2.847350913 |
| GO REGULATION OF PEPTIDE SECRETION | 17 | 5.32541 | 0.00143302 | 2.843747748 |
| GO POSITIVE REGULATION OF INTRACELLULAR TRANSPORT | 11 | 2.35921 | 0.0014784 | 2.830208046 |
| GO PROTEIN LIPID COMPLEX SUBUNIT ORGANIZATION | 6 | 0.572625 | 0.0015136 | 2.819988881 |
| GO PROTEIN PROCESSING | 11 | 2.37067 | 0.00153654 | 2.813456129 |
| GO POSITIVE REGULATION OF INFLAMMATORY RESPONSE | 9 | 1.55754 | 0.00157715 | 2.802127 |
| GO CHEMOKINE BIOSYNTHETIC PROCESS | 4 | 0.160335 | 0.00162323 | 2.789619939 |
| GO REGULATION OF AMYLOID BETA CLEARANCE | 4 | 0.160335 | 0.00162323 | 2.789619939 |
| GO REGULATION OF AUTOPHAGY OF MITOCHONDRION IN RESPONSE TO MITOCHONDRIAL DEPOLARIZATION | 4 | 0.160335 | 0.00162323 | 2.789619939 |
| GO RESPONSE TO LEUCINE | 4 | 0.160335 | 0.00162323 | 2.789619939 |
| GO DNA DAMAGE RESPONSE SIGNAL TRANSDUCTION BY P53 CLASS MEDIATOR | 8 | 1.20251 | 0.00164824 | 2.78297955 |
| GO REGULATION OF CELL CYCLE ARREST | 8 | 1.20251 | 0.00164824 | 2.78297955 |
| GO BLOOD VESSEL MORPHOGENESIS | 19 | 6.52792 | 0.00165289 | 2.781756048 |
| GO CELLULAR RESPONSE TO STEROID HORMONE STIMULUS | 12 | 2.85167 | 0.00168367 | 2.773743026 |
| GO INTERACTION WITH SYMBIONT | 7 | 0.881842 | 0.00174778 | 2.757513235 |
| GO NEGATIVE REGULATION OF AUTOPHAGY | 7 | 0.881842 | 0.00174778 | 2.757513235 |
| GO INTRACELLULAR TRANSPORT | 40 | 20.6832 | 0.00176553 | 2.753124898 |
| GO FAT CELL DIFFERENTIATION | 11 | 2.41648 | 0.00177419 | 2.750999873 |
| GO REGULATION OF ASTROCYTE DIFFERENTIATION | 5 | 0.355027 | 0.00186515 | 2.729286235 |
| GO CELL CYCLE PHASE TRANSITION | 19 | 6.60809 | 0.0019138 | 2.71810345 |
| GO CALCIUM MEDIATED SIGNALING | 11 | 2.45083 | 0.00197661 | 2.704079012 |
| GO CELL CYCLE CHECKPOINT | 11 | 2.45083 | 0.00197661 | 2.704079012 |
| GO INTERSTRAND CROSS LINK REPAIR | 6 | 0.606982 | 0.00197661 | 2.704079012 |
| GO MITOCHONDRIAL TRANSCRIPTION | 4 | 0.171787 | 0.00197661 | 2.704079012 |
| GO POSITIVE REGULATION OF AMYLOID PRECURSOR PROTEIN CATABOLIC PROCESS | 4 | 0.171787 | 0.00197661 | 2.704079012 |
| GO POSITIVE REGULATION OF GLUCONEOGENESIS | 4 | 0.171787 | 0.00197661 | 2.704079012 |
| GO POSITIVE REGULATION OF MITOCHONDRIAL FISSION | 4 | 0.171787 | 0.00197661 | 2.704079012 |

| | | | | |
|---|---|---|---|---|
| GO PROTEIN LOCALIZATION TO MITOCHONDRION | 9 | 1.6148 | 0.00197661 | 2.704079012 |
| GO REGULATION OF NEUROTRANSMITTER TRANSPORT | 9 | 1.6148 | 0.00197661 | 2.704079012 |
| GO TUMOR NECROSIS FACTOR SUPERFAMILY CYTOKINE PRODUCTION | 9 | 1.6148 | 0.00197661 | 2.704079012 |
| GO INTERLEUKIN 1 BETA PRODUCTION | 7 | 0.904747 | 0.00198214 | 2.702865674 |
| GO PEPTIDYL LYSINE MODIFICATION | 15 | 4.40921 | 0.00198214 | 2.702865674 |
| GO DEVELOPMENTAL CELL GROWTH | 11 | 2.46229 | 0.00203536 | 2.691358765 |
| GO REGULATION OF DENDRITE DEVELOPMENT | 9 | 1.62625 | 0.00206824 | 2.684399067 |
| GO FATTY ACID METABOLIC PROCESS | 12 | 2.93184 | 0.00210053 | 2.677671111 |
| GO TELOMERE CAPPING | 6 | 0.618435 | 0.00213344 | 2.670919567 |
| GO MYELOID CELL HOMEOSTASIS | 9 | 1.63771 | 0.00216332 | 2.664879235 |
| GO REPRODUCTION | 34 | 16.5374 | 0.00216332 | 2.664879235 |
| GO PEPTIDE SECRETION | 19 | 6.69971 | 0.00221646 | 2.654340102 |
| GO ION HOMEOSTASIS | 23 | 9.13909 | 0.00221676 | 2.654281324 |
| GO CARBOHYDRATE TRANSMEMBRANE TRANSPORT | 8 | 1.27123 | 0.00227485 | 2.643047235 |
| GO NEGATIVE REGULATION OF CELL GROWTH | 10 | 2.06145 | 0.00228127 | 2.641823311 |
| GO ENDOCYTOSIS | 18 | 6.13854 | 0.00228882 | 2.64038836 |
| GO MUSCLE SYSTEM PROCESS | 16 | 5.01619 | 0.00228882 | 2.64038836 |
| GO NEGATIVE REGULATION OF HYDROLASE ACTIVITY | 16 | 5.01619 | 0.00228882 | 2.64038836 |
| GO POSITIVE REGULATION OF PROTEIN TYROSINE KINASE ACTIVITY | 6 | 0.629887 | 0.00230738 | 2.636880876 |
| GO EATING BEHAVIOR | 5 | 0.377932 | 0.00232791 | 2.633033814 |
| GO SUPEROXIDE ANION GENERATION | 5 | 0.377932 | 0.00232791 | 2.633033814 |
| GO CHAPERONE MEDIATED AUTOPHAGY | 4 | 0.18324 | 0.00235699 | 2.62764226 |
| GO NEGATIVE REGULATION OF CELLULAR RESPONSE TO DRUG | 4 | 0.18324 | 0.00235699 | 2.62764226 |
| GO POSITIVE REGULATION OF ASTROCYTE DIFFERENTIATION | 4 | 0.18324 | 0.00235699 | 2.62764226 |
| GO REGULATION OF BROWN FAT CELL DIFFERENTIATION | 4 | 0.18324 | 0.00235699 | 2.62764226 |
| GO REGULATION OF TRANSPORTER ACTIVITY | 12 | 2.97765 | 0.00235699 | 2.62764226 |
| GO CHEMOKINE PRODUCTION | 7 | 0.939105 | 0.0023824 | 2.62298532 |
| GO REGULATION OF RESPONSE TO ENDOPLASMIC RETICULUM STRESS | 7 | 0.939105 | 0.0023824 | 2.62298532 |
| GO REGULATION OF IMMUNE RESPONSE | 26 | 11.1433 | 0.00239791 | 2.620167121 |
| GO DNA DEPENDENT DNA REPLICATION | 9 | 1.67206 | 0.00244356 | 2.611976993 |
| GO STEROID METABOLIC PROCESS | 13 | 3.48156 | 0.00245509 | 2.609932583 |
| GO RECEPTOR METABOLIC PROCESS | 10 | 2.09581 | 0.00253302 | 2.596361381 |
| GO REGULATION OF REACTIVE OXYGEN SPECIES BIOSYNTHETIC PROCESS | 7 | 0.950557 | 0.0025392 | 2.595303091 |
| GO STRIATED MUSCLE CELL DIFFERENTIATION | 12 | 3.01201 | 0.00258141 | 2.588143012 |
| GO NEGATIVE REGULATION OF OXIDATIVE STRESS INDUCED NEURON INTRINSIC APOPTOTIC SIGNALING PATHWAY | 3 | 0.0572625 | 0.00263491 | 2.579234214 |
| GO POSITIVE REGULATION OF ASTROCYTE ACTIVATION | 3 | 0.0572625 | 0.00263491 | 2.579234214 |
| GO POSITIVE REGULATION OF MAPKKK CASCADE BY FIBROBLAST GROWTH FACTOR RECEPTOR SIGNALING PATHWAY | 3 | 0.0572625 | 0.00263491 | 2.579234214 |
| GO REGULATION OF INTERLEUKIN 1 BIOSYNTHETIC PROCESS | 3 | 0.0572625 | 0.00263491 | 2.579234214 |
| GO REGULATION OF SPONTANEOUS SYNAPTIC TRANSMISSION | 3 | 0.0572625 | 0.00263491 | 2.579234214 |
| GO RESPONSE TO CAMPTOTHECIN | 3 | 0.0572625 | 0.00263491 | 2.579234214 |
| GO RESPONSE TO FLUORIDE | 3 | 0.0572625 | 0.00263491 | 2.579234214 |
| GO GENERATION OF PRECURSOR METABOLITES AND ENERGY | 17 | 5.65753 | 0.00264497 | 2.577579249 |
| GO REGULATION OF MUSCLE CELL DIFFERENTIATION | 9 | 1.69497 | 0.00264497 | 2.577579249 |
| GO CHROMOSOME SEGREGATION | 13 | 3.52737 | 0.00273088 | 2.563697383 |
| GO ORGANIC CYCLIC COMPOUND CATABOLIC PROCESS | 19 | 6.83714 | 0.00273088 | 2.563697383 |

| | | | | |
|---|---|---|---|---|
| GO POSITIVE REGULATION OF IMMUNE SYSTEM PROCESS | 27 | 11.9335 | 0.00278134 | 2.555745918 |
| GO NEGATIVE REGULATION OF PROTEIN OLIGOMERIZATION | 4 | 0.194692 | 0.00278674 | 2.554903549 |
| GO POSITIVE REGULATION OF SUPEROXIDE ANION GENERATION | 4 | 0.194692 | 0.00278674 | 2.554903549 |
| GO REVERSE CHOLESTEROL TRANSPORT | 4 | 0.194692 | 0.00278674 | 2.554903549 |
| GO CELLULAR RESPIRATION | 10 | 2.13016 | 0.00280317 | 2.552350563 |
| GO CELLULAR LIPID METABOLIC PROCESS | 24 | 9.97513 | 0.00283431 | 2.547552651 |
| GO REGULATION OF SYNAPTIC VESICLE TRANSPORT | 7 | 0.973462 | 0.00283431 | 2.547552651 |
| GO SECONDARY ALCOHOL METABOLIC PROCESS | 7 | 0.973462 | 0.00283431 | 2.547552651 |
| GO ACTIVATION OF MAPK ACTIVITY | 9 | 1.71787 | 0.00286815 | 2.542398139 |
| GO POSITIVE REGULATION OF NEURON DIFFERENTIATION | 14 | 4.06564 | 0.00287624 | 2.541174878 |
| GO LEUKOCYTE DIFFERENTIATION | 17 | 5.7148 | 0.00291786 | 2.53493555 |
| GO REGULATION OF VASCULATURE DEVELOPMENT | 13 | 3.56173 | 0.00294192 | 2.531369141 |
| GO REGULATION OF PLASMA LIPOPROTEIN PARTICLE LEVELS | 7 | 0.984915 | 0.00301353 | 2.520924481 |
| GO CELL CELL SIGNALING BY WNT | 17 | 5.74915 | 0.00310248 | 2.508291009 |
| GO MEMBRANE PROTEIN PROTEOLYSIS | 6 | 0.675697 | 0.00310248 | 2.508291009 |
| GO NEGATIVE REGULATION OF RNA CATABOLIC PROCESS | 6 | 0.675697 | 0.00310248 | 2.508291009 |
| GO TUBE DEVELOPMENT | 26 | 11.3609 | 0.00310248 | 2.508291009 |
| GO POSITIVE REGULATION OF MITOTIC CELL CYCLE | 9 | 1.74078 | 0.00311088 | 2.507116741 |
| GO REGULATION OF EXTRINSIC APOPTOTIC SIGNALING PATHWAY | 9 | 1.74078 | 0.00311088 | 2.507116741 |
| GO POSITIVE REGULATION OF SMOOTH MUSCLE CELL MIGRATION | 5 | 0.41229 | 0.0031641 | 2.499749799 |
| GO MEMBRANE PROTEIN INTRACELLULAR DOMAIN PROTEOLYSIS | 4 | 0.206145 | 0.00328756 | 2.483126312 |
| GO POSITIVE REGULATION OF RESPONSE TO OXIDATIVE STRESS | 4 | 0.206145 | 0.00328756 | 2.483126312 |
| GO POSITIVE REGULATION OF LIPID TRANSPORT | 6 | 0.68715 | 0.00334604 | 2.475468872 |
| GO CHROMATIN ORGANIZATION INVOLVED IN REGULATION OF TRANSCRIPTION | 8 | 1.3743 | 0.0034988 | 2.456080882 |
| GO REGULATION OF ION TRANSPORT | 20 | 7.60446 | 0.00352583 | 2.452738631 |
| GO TRANSMEMBRANE TRANSPORT | 35 | 17.7972 | 0.00360934 | 2.442572205 |
| GO CHROMOSOME SEPARATION | 7 | 1.01927 | 0.00360996 | 2.44249761 |
| GO CANONICAL WNT SIGNALING PATHWAY | 13 | 3.65335 | 0.00366213 | 2.436266243 |
| GO REGULATION OF CATION TRANSMEMBRANE TRANSPORT | 13 | 3.65335 | 0.00366213 | 2.436266243 |
| GO REGULATION OF FAT CELL DIFFERENTIATION | 8 | 1.38575 | 0.00366244 | 2.436229481 |
| GO INTERLEUKIN 1 BIOSYNTHETIC PROCESS | 3 | 0.068715 | 0.00371719 | 2.42978524 |
| GO REGULATION OF MITOCHONDRIAL ATP SYNTHESIS COUPLED ELECTRON TRANSPORT | 3 | 0.068715 | 0.00371719 | 2.42978524 |
| GO REGULATION OF ENDOCYTOSIS | 10 | 2.22178 | 0.00374658 | 2.42636499 |
| GO DEVELOPMENTAL MATURATION | 12 | 3.17234 | 0.00385711 | 2.413737975 |
| GO MAINTENANCE OF LOCATION | 13 | 3.67625 | 0.00385711 | 2.413737975 |
| GO NEGATIVE REGULATION OF LIPID STORAGE | 4 | 0.217597 | 0.0038658 | 2.412760618 |
| GO MONOCARBOXYLIC ACID METABOLIC PROCESS | 16 | 5.31396 | 0.00397135 | 2.401061836 |
| GO DENDRITIC SPINE DEVELOPMENT | 7 | 1.04218 | 0.00404082 | 2.393530495 |
| GO REGULATION OF ORGAN GROWTH | 7 | 1.04218 | 0.00404082 | 2.393530495 |
| GO MYELOID LEUKOCYTE DIFFERENTIATION | 10 | 2.25614 | 0.00418198 | 2.378618048 |
| GO PROTEIN ACYLATION | 11 | 2.72569 | 0.00427753 | 2.368806936 |
| GO INTERLEUKIN 1 PRODUCTION | 7 | 1.05363 | 0.00428873 | 2.367671294 |
| GO NEGATIVE REGULATION OF EPITHELIAL CELL APOPTOTIC PROCESS | 5 | 0.446647 | 0.0043159 | 2.364928627 |
| GO NERVOUS SYSTEM PROCESS | 32 | 15.8388 | 0.00444427 | 2.352199565 |
| GO CATECHOLAMINE BIOSYNTHETIC PROCESS | 4 | 0.22905 | 0.00452067 | 2.344797194 |

| | | | | |
|---|---|---|---|---|
| GO MITOTIC G2 DNA DAMAGE CHECKPOINT | 4 | 0.22905 | 0.00452067 | 2.344797194 |
| GO REGULATION OF NEUROTRANSMITTER UPTAKE | 4 | 0.22905 | 0.00452067 | 2.344797194 |
| GO REGULATION OF PROTEIN HOMOOLIGOMERIZATION | 4 | 0.22905 | 0.00452067 | 2.344797194 |
| GO STEROL TRANSPORT | 7 | 1.06508 | 0.00452302 | 2.344571492 |
| GO POSITIVE REGULATION OF SUPRAMOLECULAR FIBER ORGANIZATION | 10 | 2.2905 | 0.0046387 | 2.333603714 |
| GO ANATOMICAL STRUCTURE MATURATION | 9 | 1.8553 | 0.0047011 | 2.327800511 |
| GO BROWN FAT CELL DIFFERENTIATION | 5 | 0.4581 | 0.00473091 | 2.325055314 |
| GO POSITIVE REGULATION OF GLIAL CELL DIFFERENTIATION | 5 | 0.4581 | 0.00473091 | 2.325055314 |
| GO REGULATION OF PROTEIN DEACETYLATION | 5 | 0.4581 | 0.00473091 | 2.325055314 |
| GO REGULATION OF VACUOLE ORGANIZATION | 5 | 0.4581 | 0.00473091 | 2.325055314 |
| GO IMMUNE EFFECTOR PROCESS | 28 | 13.1246 | 0.00496806 | 2.303813168 |
| GO ORGANIC HYDROXY COMPOUND METABOLIC PROCESS | 16 | 5.43994 | 0.00500129 | 2.300917962 |
| GO AMYLOID BETA CLEARANCE BY CELLULAR CATABOLIC PROCESS | 3 | 0.0801675 | 0.00505302 | 2.296448983 |
| GO LATE ENDOSOME TO LYSOSOME TRANSPORT | 3 | 0.0801675 | 0.00505302 | 2.296448983 |
| GO POSITIVE REGULATION OF CELLULAR RESPIRATION | 3 | 0.0801675 | 0.00505302 | 2.296448983 |
| GO POSITIVE REGULATION OF PROTEIN MODIFICATION BY SMALL PROTEIN CONJUGATION OR REMOVAL | 8 | 1.46592 | 0.00505302 | 2.296448983 |
| GO SPONTANEOUS SYNAPTIC TRANSMISSION | 3 | 0.0801675 | 0.00505302 | 2.296448983 |
| GO LEUKOCYTE PROLIFERATION | 12 | 3.28687 | 0.00509378 | 2.292959816 |
| GO HOMEOSTASIS OF NUMBER OF CELLS | 11 | 2.79441 | 0.00510179 | 2.292277422 |
| GO REGULATION OF GLIAL CELL DIFFERENTIATION | 6 | 0.755865 | 0.00512591 | 2.290229023 |
| GO INFLAMMATORY CELL APOPTOTIC PROCESS | 4 | 0.240502 | 0.00513288 | 2.289638889 |
| GO MITOCHONDRIAL GENOME MAINTENANCE | 4 | 0.240502 | 0.00513288 | 2.289638889 |
| GO MITOPHAGY | 4 | 0.240502 | 0.00513288 | 2.289638889 |
| GO NEGATIVE REGULATION OF INTRACELLULAR PROTEIN TRANSPORT | 5 | 0.469552 | 0.00513288 | 2.289638889 |
| GO NEGATIVE REGULATION OF STRIATED MUSCLE CELL APOPTOTIC PROCESS | 4 | 0.240502 | 0.00513288 | 2.289638889 |
| GO NEUROTRANSMITTER UPTAKE | 5 | 0.469552 | 0.00513288 | 2.289638889 |
| GO POSITIVE REGULATION OF NUCLEOTIDE BIOSYNTHETIC PROCESS | 4 | 0.240502 | 0.00513288 | 2.289638889 |
| GO REGULATION OF SUPEROXIDE ANION GENERATION | 4 | 0.240502 | 0.00513288 | 2.289638889 |
| GO RESPONSE TO ELECTRICAL STIMULUS | 5 | 0.469552 | 0.00513288 | 2.289638889 |
| GO RESPONSE TO HYPEROXIA | 4 | 0.240502 | 0.00513288 | 2.289638889 |
| GO RESPONSE TO SALT STRESS | 4 | 0.240502 | 0.00513288 | 2.289638889 |
| GO CELLULAR RESPONSE TO ACID CHEMICAL | 10 | 2.33631 | 0.00521258 | 2.282947267 |
| GO STEROL METABOLIC PROCESS | 7 | 1.09944 | 0.00522842 | 2.281629533 |
| GO NEGATIVE REGULATION OF PROTEIN COMPLEX ASSEMBLY | 8 | 1.48882 | 0.00546029 | 2.262784291 |
| GO MEMBRANE PROTEIN ECTODOMAIN PROTEOLYSIS | 5 | 0.481005 | 0.00562865 | 2.249595756 |
| GO RESPONSE TO TESTOSTERONE | 5 | 0.481005 | 0.00562865 | 2.249595756 |
| GO NEGATIVE REGULATION OF LIPID METABOLIC PROCESS | 6 | 0.77877 | 0.0058291 | 2.234398494 |
| GO REGULATION OF POSTSYNAPSE ORGANIZATION | 7 | 1.12234 | 0.00585334 | 2.232596249 |
| GO INTERLEUKIN 6 BIOSYNTHETIC PROCESS | 4 | 0.251955 | 0.00594047 | 2.226179193 |
| GO NEGATIVE REGULATION OF SMOOTH MUSCLE CELL MIGRATION | 4 | 0.251955 | 0.00594047 | 2.226179193 |
| GO HEART DEVELOPMENT | 17 | 6.13854 | 0.00600124 | 2.221759005 |
| GO REGULATION OF PROTEIN TARGETING TO MITOCHONDRION | 5 | 0.492457 | 0.00617611 | 2.209284978 |
| GO POSITIVE REGULATION OF DENDRITE DEVELOPMENT | 6 | 0.790222 | 0.00622683 | 2.205732991 |
| GO SIGNAL RELEASE FROM SYNAPSE | 9 | 1.94692 | 0.00628557 | 2.201655333 |
| GO SKELETAL MUSCLE ORGAN DEVELOPMENT | 9 | 1.94692 | 0.00628557 | 2.201655333 |

| | | | | |
|---|---|---|---|---|
| GO PROTEIN TARGETING TO MITOCHONDRION | 7 | 1.14525 | 0.00650975 | 2.18643569 |
| GO POSITIVE REGULATION OF LIPID METABOLIC PROCESS | 8 | 1.53463 | 0.00652834 | 2.185197235 |
| GO NEGATIVE REGULATION OF ESTABLISHMENT OF PROTEIN LOCALIZATION TO MITOCHONDRION | 3 | 0.09162 | 0.00657216 | 2.182291872 |
| GO POSITIVE REGULATION OF ASPARTIC TYPE PEPTIDASE ACTIVITY | 3 | 0.09162 | 0.00657216 | 2.182291872 |
| GO POSITIVE REGULATION OF OXIDATIVE PHOSPHORYLATION | 3 | 0.09162 | 0.00657216 | 2.182291872 |
| GO POSITIVE REGULATION OF RESPONSE TO REACTIVE OXYGEN SPECIES | 3 | 0.09162 | 0.00657216 | 2.182291872 |
| GO WOUND HEALING | 17 | 6.1958 | 0.00657216 | 2.182291872 |
| GO SMALL MOLECULE BIOSYNTHETIC PROCESS | 19 | 7.42122 | 0.00662499 | 2.178814773 |
| GO ATP BIOSYNTHETIC PROCESS | 5 | 0.50391 | 0.00669071 | 2.174527794 |
| GO GLIAL CELL PROLIFERATION | 5 | 0.50391 | 0.00669071 | 2.174527794 |
| GO REGULATION OF DOUBLE STRAND BREAK REPAIR VIA HOMOLOGOUS RECOMBINATION | 5 | 0.50391 | 0.00669071 | 2.174527794 |
| GO REGULATION OF INSULIN LIKE GROWTH FACTOR RECEPTOR SIGNALING PATHWAY | 4 | 0.263407 | 0.00675021 | 2.170682716 |
| GO TUMOR NECROSIS FACTOR BIOSYNTHETIC PROCESS | 4 | 0.263407 | 0.00675021 | 2.170682716 |
| GO REGULATION OF ANATOMICAL STRUCTURE SIZE | 16 | 5.63463 | 0.00689164 | 2.161677417 |
| GO NEGATIVE REGULATION OF FAT CELL DIFFERENTIATION | 5 | 0.515362 | 0.0073185 | 2.135577923 |
| GO REGULATION OF GLUCONEOGENESIS | 5 | 0.515362 | 0.0073185 | 2.135577923 |
| GO ENDOTHELIAL CELL PROLIFERATION | 8 | 1.56899 | 0.00738261 | 2.131790073 |
| GO CELLULAR CARBOHYDRATE METABOLIC PROCESS | 11 | 2.94329 | 0.00739597 | 2.131004859 |
| GO LEUKOCYTE MEDIATED IMMUNITY | 21 | 8.77261 | 0.00742081 | 2.129548688 |
| GO CELL PROJECTION ORGANIZATION | 33 | 17.1329 | 0.00750649 | 2.12456309 |
| GO INTRINSIC APOPTOTIC SIGNALING PATHWAY IN RESPONSE TO DNA DAMAGE | 7 | 1.17961 | 0.00752155 | 2.123692653 |
| GO MODULATION BY HOST OF VIRAL PROCESS | 4 | 0.27486 | 0.00768577 | 2.114312616 |
| GO RESPONSE TO MUSCLE ACTIVITY | 4 | 0.27486 | 0.00768577 | 2.114312616 |
| GO SMOOTH MUSCLE CELL APOPTOTIC PROCESS | 4 | 0.27486 | 0.00768577 | 2.114312616 |
| GO VESICLE LOCALIZATION | 12 | 3.47011 | 0.00768577 | 2.114312616 |
| GO RESPONSE TO NUTRIENT | 10 | 2.47374 | 0.00768752 | 2.114213741 |
| GO NEGATIVE REGULATION OF CYTOKINE PRODUCTION | 11 | 2.9662 | 0.00779172 | 2.108366662 |
| GO ANATOMICAL STRUCTURE FORMATION INVOLVED IN MORPHOGENESIS | 26 | 12.174 | 0.00783375 | 2.106030292 |
| GO POSITIVE REGULATION OF TRANSPORTER ACTIVITY | 7 | 1.20251 | 0.00830521 | 2.080649382 |
| GO REGULATION OF NUCLEOCYTOPLASMIC TRANSPORT | 7 | 1.20251 | 0.00830521 | 2.080649382 |
| GO POSITIVE REGULATION OF VASCULATURE DEVELOPMENT | 9 | 2.03854 | 0.00835377 | 2.078117486 |
| GO HYPEROSMOTIC SALINITY RESPONSE | 3 | 0.103072 | 0.00836687 | 2.077436979 |
| GO MITOCHONDRIAL FRAGMENTATION INVOLVED IN APOPTOTIC PROCESS | 3 | 0.103072 | 0.00836687 | 2.077436979 |
| GO SINGLE STRAND BREAK REPAIR | 3 | 0.103072 | 0.00836687 | 2.077436979 |
| GO SKIN MORPHOGENESIS | 3 | 0.103072 | 0.00836687 | 2.077436979 |
| GO UBIQUITIN RECYCLING | 3 | 0.103072 | 0.00836687 | 2.077436979 |
| GO MYELOID CELL DIFFERENTIATION | 14 | 4.6039 | 0.008475 | 2.071860293 |
| GO NEGATIVE REGULATION OF TOR SIGNALING | 5 | 0.538267 | 0.00856978 | 2.067030327 |
| GO REGULATION OF PROTEIN LOCALIZATION TO MEMBRANE | 9 | 2.05 | 0.00860154 | 2.065423787 |
| GO EXTRINSIC APOPTOTIC SIGNALING PATHWAY | 10 | 2.51955 | 0.00863208 | 2.063884543 |
| GO NEGATIVE REGULATION OF NEURON DIFFERENTIATION | 10 | 2.51955 | 0.00863208 | 2.063884543 |
| GO POSITIVE REGULATION OF PROTEIN OLIGOMERIZATION | 4 | 0.286312 | 0.00863208 | 2.063884543 |
| GO REGULATION OF DNA REPLICATION | 7 | 1.21396 | 0.00863208 | 2.063884543 |
| GO REGULATION OF NEUROTRANSMITTER SECRETION | 7 | 1.21396 | 0.00863208 | 2.063884543 |
| GO REGULATION OF OXIDATIVE PHOSPHORYLATION | 4 | 0.286312 | 0.00863208 | 2.063884543 |

| | | | | |
|---|---|---|---|---|
| GO REGULATION OF LIPID BIOSYNTHETIC PROCESS | 9 | 2.06145 | 0.00886625 | 2.052260027 |
| GO CARBOHYDRATE TRANSPORT | 8 | 1.62625 | 0.0089309 | 2.049104773 |
| GO CARDIOCYTE DIFFERENTIATION | 8 | 1.62625 | 0.0089309 | 2.049104773 |
| GO REGULATION OF FATTY ACID METABOLIC PROCESS | 5 | 0.54972 | 0.00924797 | 2.033953588 |
| GO POSITIVE REGULATION OF LIPID BIOSYNTHETIC PROCESS | 6 | 0.87039 | 0.00948604 | 2.022915048 |
| GO ADULT BEHAVIOR | 8 | 1.64916 | 0.00972828 | 2.011963938 |
| GO NEURON DEATH IN RESPONSE TO OXIDATIVE STRESS | 4 | 0.297765 | 0.00974449 | 2.011240886 |
| GO POSITIVE REGULATION OF BEHAVIOR | 4 | 0.297765 | 0.00974449 | 2.011240886 |
| GO POSITIVE REGULATION OF RESPONSE TO DRUG | 4 | 0.297765 | 0.00974449 | 2.011240886 |
| GO POSITIVE REGULATION OF SECRETION | 14 | 4.68407 | 0.00980154 | 2.008705683 |
| GO DNA MODIFICATION | 7 | 1.24832 | 0.00994812 | 2.002258985 |
| GO NUCLEOTIDE EXCISION REPAIR | 7 | 1.24832 | 0.00994812 | 2.002258985 |
| GO ANIMAL ORGAN REGENERATION | 6 | 0.881842 | 0.010018 | 1.999218973 |
| GO CELLULAR RESPONSE TO UV | 6 | 0.881842 | 0.010018 | 1.999218973 |
| GO REGULATION OF LEUKOCYTE MIGRATION | 9 | 2.10726 | 0.010162 | 1.993020809 |
| GO POSITIVE REGULATION OF PEPTIDE SECRETION | 11 | 3.08072 | 0.0101737 | 1.992521073 |
| GO CELLULAR RESPONSE TO POTASSIUM ION | 3 | 0.114525 | 0.0103172 | 1.986438151 |
| GO CYTOKINE METABOLIC PROCESS | 7 | 1.25977 | 0.0103172 | 1.986438151 |
| GO INFLAMMATORY RESPONSE TO WOUNDING | 3 | 0.114525 | 0.0103172 | 1.986438151 |
| GO NEGATIVE REGULATION OF MUSCLE ADAPTATION | 3 | 0.114525 | 0.0103172 | 1.986438151 |
| GO NEGATIVE REGULATION OF PROTEIN HOMOOLIGOMERIZATION | 3 | 0.114525 | 0.0103172 | 1.986438151 |
| GO NEURON PROJECTION MAINTENANCE | 3 | 0.114525 | 0.0103172 | 1.986438151 |
| GO POSITIVE REGULATION OF CHEMOKINE BIOSYNTHETIC PROCESS | 3 | 0.114525 | 0.0103172 | 1.986438151 |
| GO POSITIVE REGULATION OF DOUBLE STRAND BREAK REPAIR VIA HOMOLOGOUS RECOMBINATION | 3 | 0.114525 | 0.0103172 | 1.986438151 |
| GO POSITIVE REGULATION OF MITOPHAGY | 3 | 0.114525 | 0.0103172 | 1.986438151 |
| GO REGULATION OF REMOVAL OF SUPEROXIDE RADICALS | 3 | 0.114525 | 0.0103172 | 1.986438151 |
| GO STRIATED MUSCLE ATROPHY | 3 | 0.114525 | 0.0103172 | 1.986438151 |
| GO CELLULAR RESPONSE TO BIOTIC STIMULUS | 10 | 2.59972 | 0.0105746 | 1.975736051 |
| GO REGULATION OF VASCULAR SMOOTH MUSCLE CELL PROLIFERATION | 5 | 0.572625 | 0.0106628 | 1.972128737 |
| GO RNA STABILIZATION | 5 | 0.572625 | 0.0106628 | 1.972128737 |
| GO NEGATIVE REGULATION OF MUSCLE HYPERTROPHY | 4 | 0.309217 | 0.0107832 | 1.96725234 |
| GO RESPONSE TO AMINO ACID | 7 | 1.27123 | 0.0107832 | 1.96725234 |
| GO REGULATION OF CELLULAR RESPONSE TO HEAT | 6 | 0.904747 | 0.0111329 | 1.953391692 |
| GO POSITIVE REGULATION OF ION TRANSMEMBRANE TRANSPORT | 8 | 1.69497 | 0.011191 | 1.951131104 |
| GO ENDOMEMBRANE SYSTEM ORGANIZATION | 14 | 4.76424 | 0.0112021 | 1.950700555 |
| GO REGULATION OF UBIQUITIN PROTEIN TRANSFERASE ACTIVITY | 5 | 0.584077 | 0.0115182 | 1.938615385 |
| GO POSITIVE REGULATION OF LIPID LOCALIZATION | 6 | 0.9162 | 0.0117856 | 1.928648303 |
| GO PROTEIN LOCALIZATION TO SYNAPSE | 6 | 0.9162 | 0.0117856 | 1.928648303 |
| GO CARDIAC MUSCLE CELL DIFFERENTIATION | 7 | 1.29413 | 0.0118251 | 1.927195178 |
| GO RESPONSE TO CYTOKINE | 27 | 13.2849 | 0.0118575 | 1.926006867 |
| GO CELL MORPHOGENESIS INVOLVED IN NEURON DIFFERENTIATION | 17 | 6.59664 | 0.0119917 | 1.921119245 |
| GO ADAPTIVE THERMOGENESIS | 8 | 1.71787 | 0.011999 | 1.920854947 |
| GO POSITIVE REGULATION OF HYDROLASE ACTIVITY | 20 | 8.50921 | 0.011999 | 1.920854947 |
| GO POSITIVE REGULATION OF MACROPHAGE ACTIVATION | 4 | 0.32067 | 0.011999 | 1.920854947 |
| GO POSITIVE REGULATION OF MONOOXYGENASE ACTIVITY | 4 | 0.32067 | 0.011999 | 1.920854947 |

| | | | | |
|---|---|---|---|---|
| GO RESPONSE TO INCREASED OXYGEN LEVELS | 4 | 0.32067 | 0.011999 | 1.920854947 |
| GO SKELETAL MUSCLE ADAPTATION | 4 | 0.32067 | 0.011999 | 1.920854947 |
| GO LIPID MODIFICATION | 10 | 2.65698 | 0.0121651 | 1.914884317 |
| GO CELLULAR KETONE METABOLIC PROCESS | 9 | 2.17597 | 0.0121938 | 1.913860932 |
| GO REGULATION OF STEROID METABOLIC PROCESS | 7 | 1.30558 | 0.0122717 | 1.91109527 |
| GO DNA SYNTHESIS INVOLVED IN DNA REPAIR | 5 | 0.59553 | 0.0122903 | 1.910437516 |
| GO CATECHOLAMINE UPTAKE INVOLVED IN SYNAPTIC TRANSMISSION | 3 | 0.125977 | 0.0125225 | 1.90230896 |
| GO POSITIVE REGULATION OF AMYLOID BETA FORMATION | 3 | 0.125977 | 0.0125225 | 1.90230896 |
| GO PROGRAMMED CELL DEATH IN RESPONSE TO REACTIVE OXYGEN SPECIES | 3 | 0.125977 | 0.0125225 | 1.90230896 |
| GO REGULATION OF DENDRITIC SPINE MAINTENANCE | 3 | 0.125977 | 0.0125225 | 1.90230896 |
| GO REGULATION OF DNA CATABOLIC PROCESS | 3 | 0.125977 | 0.0125225 | 1.90230896 |
| GO REGULATION OF TRANSCRIPTION FROM RNA POLYMERASE II PROMOTER IN RESPONSE TO OXIDATIVE STRESS | 3 | 0.125977 | 0.0125225 | 1.90230896 |
| GO SYNAPTIC VESICLE CYCLE | 9 | 2.18743 | 0.0125225 | 1.90230896 |
| GO TUBULIN DEACETYLATION | 3 | 0.125977 | 0.0125225 | 1.90230896 |
| GO VIRAL LATENCY | 3 | 0.125977 | 0.0125225 | 1.90230896 |
| GO NEGATIVE REGULATION OF CELL CYCLE PROCESS | 12 | 3.73351 | 0.0131047 | 1.882572917 |
| GO REGULATION OF MACROPHAGE DERIVED FOAM CELL DIFFERENTIATION | 4 | 0.332122 | 0.0132984 | 1.876200608 |
| GO ERYTHROCYTE HOMEOSTASIS | 7 | 1.33994 | 0.0140047 | 1.85372619 |
| GO HOMOLOGOUS RECOMBINATION | 5 | 0.618435 | 0.0141952 | 1.847858484 |
| GO REGULATION OF MONOOXYGENASE ACTIVITY | 5 | 0.618435 | 0.0141952 | 1.847858484 |
| GO REGULATION OF RESPONSE TO WOUNDING | 8 | 1.77514 | 0.014413 | 1.841245613 |
| GO TISSUE REMODELING | 8 | 1.77514 | 0.014413 | 1.841245613 |
| GO MYOBLAST DIFFERENTIATION | 6 | 0.96201 | 0.0145147 | 1.838191936 |
| GO MITOTIC G2 M TRANSITION CHECKPOINT | 4 | 0.343575 | 0.0147676 | 1.83069008 |
| GO NEGATIVE REGULATION OF MACROAUTOPHAGY | 4 | 0.343575 | 0.0147676 | 1.83069008 |
| GO REGULATION OF FATTY ACID OXIDATION | 4 | 0.343575 | 0.0147676 | 1.83069008 |
| GO RESPONSE TO ARSENIC CONTAINING SUBSTANCE | 4 | 0.343575 | 0.0147676 | 1.83069008 |
| GO CELL ACTIVATION INVOLVED IN IMMUNE RESPONSE | 19 | 8.0282 | 0.0148647 | 1.827843851 |
| GO PRESYNAPTIC ENDOCYTOSIS | 5 | 0.629887 | 0.0151868 | 1.818533726 |
| GO PHOSPHOLIPID EFFLUX | 3 | 0.13743 | 0.0152221 | 1.817525429 |
| GO POSITIVE REGULATION OF INTERLEUKIN 6 BIOSYNTHETIC PROCESS | 3 | 0.13743 | 0.0152221 | 1.817525429 |
| GO REGULATION OF LYSOSOMAL LUMEN PH | 3 | 0.13743 | 0.0152221 | 1.817525429 |
| GO NEUTRAL LIPID METABOLIC PROCESS | 7 | 1.3743 | 0.0158454 | 1.800096793 |
| GO POSITIVE REGULATION OF PROTEIN LOCALIZATION TO MEMBRANE | 7 | 1.3743 | 0.0158454 | 1.800096793 |
| GO SYNAPTIC VESICLE EXOCYTOSIS | 7 | 1.3743 | 0.0158454 | 1.800096793 |
| GO REGULATION OF STEROID BIOSYNTHETIC PROCESS | 6 | 0.984915 | 0.0160747 | 1.793857124 |
| GO ANION HOMEOSTASIS | 5 | 0.64134 | 0.0161885 | 1.79079339 |
| GO POSITIVE REGULATION OF INTRINSIC APOPTOTIC SIGNALING PATHWAY | 5 | 0.64134 | 0.0161885 | 1.79079339 |
| GO REGULATION OF CELL CYCLE PHASE TRANSITION | 14 | 4.98184 | 0.0161885 | 1.79079339 |
| GO SECOND MESSENGER MEDIATED SIGNALING | 14 | 4.98184 | 0.0161885 | 1.79079339 |
| GO G2 DNA DAMAGE CHECKPOINT | 4 | 0.355027 | 0.0162106 | 1.79020091 |
| GO POSITIVE REGULATION OF DOUBLE STRAND BREAK REPAIR | 4 | 0.355027 | 0.0162106 | 1.79020091 |
| GO REGULATION OF GLIAL CELL PROLIFERATION | 4 | 0.355027 | 0.0162106 | 1.79020091 |
| GO VASODILATION | 4 | 0.355027 | 0.0162106 | 1.79020091 |
| GO NEGATIVE REGULATION OF PEPTIDASE ACTIVITY | 10 | 2.78296 | 0.0163741 | 1.785842562 |

| | | | | |
|---|---|---|---|---|
| GO ESTABLISHMENT OF ORGANELLE LOCALIZATION | 15 | 5.61172 | 0.0170725 | 1.767702879 |
| GO REGULATION OF CELL CYCLE G1 S PHASE TRANSITION | 8 | 1.8324 | 0.0170927 | 1.76718933 |
| GO RESPONSE TO BIOTIC STIMULUS | 23 | 10.8455 | 0.0172316 | 1.763674395 |
| GO POSITIVE REGULATION OF BLOOD VESSEL DIAMETER | 5 | 0.652792 | 0.0172728 | 1.762637256 |
| GO ACTIVATION OF PROTEIN KINASE B ACTIVITY | 4 | 0.36648 | 0.0178556 | 1.748225552 |
| GO CELLULAR RESPONSE TO DEXAMETHASONE STIMULUS | 4 | 0.36648 | 0.0178556 | 1.748225552 |
| GO POSITIVE REGULATION OF LEUKOCYTE MIGRATION | 7 | 1.40866 | 0.0178556 | 1.748225552 |
| GO PROTEIN LIPID COMPLEX ASSEMBLY | 4 | 0.36648 | 0.0178556 | 1.748225552 |
| GO REGULATION OF ENDOPLASMIC RETICULUM STRESS INDUCED INTRINSIC APOPTOTIC SIGNALING PATHWAY | 4 | 0.36648 | 0.0178556 | 1.748225552 |
| GO REPLICATION FORK PROCESSING | 4 | 0.36648 | 0.0178556 | 1.748225552 |
| GO HYDROGEN PEROXIDE METABOLIC PROCESS | 3 | 0.148882 | 0.0180246 | 1.744134364 |
| GO MYELOID LEUKOCYTE MEDIATED IMMUNITY | 16 | 6.26452 | 0.0180246 | 1.744134364 |
| GO POSITIVE REGULATION OF EXECUTION PHASE OF APOPTOSIS | 3 | 0.148882 | 0.0180246 | 1.744134364 |
| GO REGULATION OF METALLOPEPTIDASE ACTIVITY | 3 | 0.148882 | 0.0180246 | 1.744134364 |
| GO NEUROTRANSMITTER BIOSYNTHETIC PROCESS | 6 | 1.01927 | 0.0185251 | 1.732239439 |
| GO NEUROTRANSMITTER METABOLIC PROCESS | 7 | 1.42011 | 0.0185251 | 1.732239439 |
| GO PEPTIDE HORMONE SECRETION | 10 | 2.84022 | 0.0186762 | 1.728711484 |
| GO NEGATIVE REGULATION OF MAPK CASCADE | 8 | 1.86676 | 0.0188531 | 1.724617229 |
| GO CARDIAC MUSCLE TISSUE DEVELOPMENT | 9 | 2.34776 | 0.0191892 | 1.716943131 |
| GO ACYLGLYCEROL HOMEOSTASIS | 4 | 0.377932 | 0.0194188 | 1.711777611 |
| GO LIVER REGENERATION | 4 | 0.377932 | 0.0194188 | 1.711777611 |
| GO LONG TERM MEMORY | 4 | 0.377932 | 0.0194188 | 1.711777611 |
| GO MULTICELLULAR ORGANISM REPRODUCTION | 21 | 9.58574 | 0.0194188 | 1.711777611 |
| GO NEGATIVE REGULATION OF EXOCYTOSIS | 4 | 0.377932 | 0.0194188 | 1.711777611 |
| GO NEGATIVE REGULATION OF GENE SILENCING | 4 | 0.377932 | 0.0194188 | 1.711777611 |
| GO POSITIVE REGULATION OF MULTICELLULAR ORGANISM GROWTH | 4 | 0.377932 | 0.0194188 | 1.711777611 |
| GO POSITIVE REGULATION OF PROTEIN KINASE B SIGNALING | 8 | 1.87821 | 0.0194188 | 1.711777611 |
| GO POSITIVE REGULATION OF TRANSCRIPTION FROM RNA POLYMERASE II PROMOTER IN RESPONSE TO STRESS | 4 | 0.377932 | 0.0194188 | 1.711777611 |
| GO REGULATION OF PEPTIDYL LYSINE ACETYLATION | 5 | 0.675697 | 0.0194188 | 1.711777611 |
| GO RESPONSE TO MINERALOCORTICOID | 4 | 0.377932 | 0.0194188 | 1.711777611 |
| GO RESPONSE TO BACTERIUM | 17 | 6.96312 | 0.0196875 | 1.705809429 |
| GO CELL CYCLE G1 S PHASE TRANSITION | 10 | 2.88603 | 0.0206647 | 1.684770895 |
| GO POSITIVE REGULATION OF RESPONSE TO WOUNDING | 5 | 0.68715 | 0.0207331 | 1.683335758 |
| GO RESPONSE TO ETHANOL | 7 | 1.45447 | 0.0207331 | 1.683335758 |
| GO RIBONUCLEOSIDE TRIPHOSPHATE BIOSYNTHETIC PROCESS | 5 | 0.68715 | 0.0207331 | 1.683335758 |
| GO GLIAL CELL APOPTOTIC PROCESS | 3 | 0.160335 | 0.0210309 | 1.677142142 |
| GO LOW DENSITY LIPOPROTEIN PARTICLE REMODELING | 3 | 0.160335 | 0.0210309 | 1.677142142 |
| GO MODULATION BY HOST OF VIRAL GENOME REPLICATION | 3 | 0.160335 | 0.0210309 | 1.677142142 |
| GO NEGATIVE REGULATION OF POSTTRANSCRIPTIONAL GENE SILENCING | 3 | 0.160335 | 0.0210309 | 1.677142142 |
| GO POSITIVE REGULATION OF OXIDATIVE STRESS INDUCED CELL DEATH | 3 | 0.160335 | 0.0210309 | 1.677142142 |
| GO POSITIVE REGULATION OF TUMOR NECROSIS FACTOR BIOSYNTHETIC PROCESS | 3 | 0.160335 | 0.0210309 | 1.677142142 |
| GO REGULATION OF HELICASE ACTIVITY | 3 | 0.160335 | 0.0210309 | 1.677142142 |
| GO REGULATION OF LONG TERM SYNAPTIC DEPRESSION | 3 | 0.160335 | 0.0210309 | 1.677142142 |
| GO REGULATION OF AUTOPHAGOSOME ASSEMBLY | 4 | 0.389385 | 0.0211837 | 1.673998183 |
| GO CELLULAR RESPONSE TO XENOBIOTIC STIMULUS | 8 | 1.91257 | 0.0212812 | 1.672003887 |

| | | | | |
|---|---|---|---|---|
| GO NUCLEAR CHROMOSOME SEGREGATION | 10 | 2.90893 | 0.0215688 | 1.666174017 |
| GO POSITIVE REGULATION OF CYTOKINE BIOSYNTHETIC PROCESS | 5 | 0.698602 | 0.0219441 | 1.658682226 |
| GO ANTIBIOTIC METABOLIC PROCESS | 6 | 1.06508 | 0.0221765 | 1.654106995 |
| GO POSITIVE REGULATION OF CELL CYCLE PHASE TRANSITION | 6 | 1.06508 | 0.0221765 | 1.654106995 |
| GO REGULATION OF ANION TRANSPORT | 6 | 1.06508 | 0.0221765 | 1.654106995 |
| GO CELLULAR RESPONSE TO ALKALOID | 4 | 0.400837 | 0.0231495 | 1.635458385 |
| GO FOAM CELL DIFFERENTIATION | 4 | 0.400837 | 0.0231495 | 1.635458385 |
| GO NEURON CELLULAR HOMEOSTASIS | 4 | 0.400837 | 0.0231495 | 1.635458385 |
| GO STRIATED MUSCLE CELL APOPTOTIC PROCESS | 4 | 0.400837 | 0.0231495 | 1.635458385 |
| GO MODIFICATION DEPENDENT MACROMOLECULE CATABOLIC PROCESS | 17 | 7.0891 | 0.0232227 | 1.634087288 |
| GO AUTOPHAGOSOME ORGANIZATION | 6 | 1.07653 | 0.0232245 | 1.634053627 |
| GO FEMALE GAMETE GENERATION | 7 | 1.50028 | 0.0240522 | 1.618845194 |
| GO POSITIVE REGULATION OF ENDOCYTOSIS | 6 | 1.08799 | 0.0244102 | 1.612428662 |
| GO FATTY ACID HOMEOSTASIS | 3 | 0.171787 | 0.0245282 | 1.610334321 |
| GO HIGH DENSITY LIPOPROTEIN PARTICLE ASSEMBLY | 3 | 0.171787 | 0.0245282 | 1.610334321 |
| GO RESPONSE TO POTASSIUM ION | 3 | 0.171787 | 0.0245282 | 1.610334321 |
| GO RESPONSE TO STIMULUS INVOLVED IN REGULATION OF MUSCLE ADAPTATION | 3 | 0.171787 | 0.0245282 | 1.610334321 |
| GO REGULATION OF HEART GROWTH | 5 | 0.721507 | 0.0247109 | 1.607111437 |
| GO REGULATION OF CELLULAR KETONE METABOLIC PROCESS | 7 | 1.51173 | 0.0249047 | 1.603718685 |
| GO NEGATIVE REGULATION OF MULTI ORGANISM PROCESS | 8 | 1.96983 | 0.0249939 | 1.602165972 |
| GO CELLULAR RESPONSE TO CADMIUM ION | 4 | 0.41229 | 0.0250711 | 1.600826611 |
| GO INSULIN LIKE GROWTH FACTOR RECEPTOR SIGNALING PATHWAY | 4 | 0.41229 | 0.0250711 | 1.600826611 |
| GO NEGATIVE REGULATION OF ENDOTHELIAL CELL PROLIFERATION | 4 | 0.41229 | 0.0250711 | 1.600826611 |
| GO REGULATION OF COFACTOR METABOLIC PROCESS | 4 | 0.41229 | 0.0250711 | 1.600826611 |
| GO SKELETAL MUSCLE TISSUE REGENERATION | 4 | 0.41229 | 0.0250711 | 1.600826611 |
| GO ORGANELLE LOCALIZATION | 18 | 7.8106 | 0.0252529 | 1.597688741 |
| GO POSITIVE REGULATION OF COLD INDUCED THERMOGENESIS | 6 | 1.09944 | 0.0253213 | 1.596514001 |
| GO INTERSPECIES INTERACTION BETWEEN ORGANISMS | 22 | 10.5363 | 0.025449 | 1.594329278 |
| GO CELL MATURATION | 8 | 1.98128 | 0.0256584 | 1.590770429 |
| GO REGULATION OF RECEPTOR SIGNALING PATHWAY VIA STAT | 7 | 1.52318 | 0.0256723 | 1.590535221 |
| GO REGULATION OF LEUKOCYTE PROLIFERATION | 9 | 2.47374 | 0.0257035 | 1.590007736 |
| GO CELLULAR RESPONSE TO CORTICOSTEROID STIMULUS | 5 | 0.73296 | 0.0260175 | 1.584734437 |
| GO REGULATION OF STRIATED MUSCLE CELL DIFFERENTIATION | 6 | 1.11089 | 0.0264727 | 1.577201762 |
| GO POSITIVE REGULATION OF ION TRANSPORT | 10 | 3.01201 | 0.0268399 | 1.571219107 |
| GO TUBE MORPHOGENESIS | 20 | 9.20781 | 0.0269318 | 1.569734619 |
| GO POSITIVE REGULATION OF HEART GROWTH | 4 | 0.423742 | 0.0271976 | 1.565469418 |
| GO POSITIVE REGULATION OF TELOMERE MAINTENANCE VIA TELOMERE LENGTHENING | 4 | 0.423742 | 0.0271976 | 1.565469418 |
| GO ORGAN GROWTH | 8 | 2.00419 | 0.0272772 | 1.564200212 |
| GO AXO DENDRITIC TRANSPORT | 5 | 0.744412 | 0.0275366 | 1.560089684 |
| GO COFACTOR CATABOLIC PROCESS | 5 | 0.744412 | 0.0275366 | 1.560089684 |
| GO CATECHOLAMINE UPTAKE | 3 | 0.18324 | 0.0279751 | 1.553228352 |
| GO CELL CYCLE G2 M PHASE TRANSITION | 10 | 3.03491 | 0.0279751 | 1.553228352 |
| GO POSITIVE REGULATION OF GLIAL CELL PROLIFERATION | 3 | 0.18324 | 0.0279751 | 1.553228352 |
| GO REGULATION OF CAMP DEPENDENT PROTEIN KINASE ACTIVITY | 3 | 0.18324 | 0.0279751 | 1.553228352 |
| GO REGULATION OF INCLUSION BODY ASSEMBLY | 3 | 0.18324 | 0.0279751 | 1.553228352 |

| | | | | |
|---|---|---|---|---|
| GO REGULATION OF MITOPHAGY | 3 | 0.18324 | 0.0279751 | 1.553228352 |
| GO REGULATION OF NUCLEAR CELL CYCLE DNA REPLICATION | 3 | 0.18324 | 0.0279751 | 1.553228352 |
| GO RESPONSE TO EPINEPHRINE | 3 | 0.18324 | 0.0279751 | 1.553228352 |
| GO REGULATION OF RESPONSE TO BIOTIC STIMULUS | 7 | 1.55754 | 0.0284496 | 1.545923835 |
| GO REGULATION OF TUBE SIZE | 7 | 1.55754 | 0.0284496 | 1.545923835 |
| GO REGULATION OF INFLAMMATORY RESPONSE TO WOUNDING | 2 | 0.0343575 | 0.0286711 | 1.542555645 |
| GO POSITIVE REGULATION OF DNA BIOSYNTHETIC PROCESS | 5 | 0.755865 | 0.0289971 | 1.537645434 |
| GO NEGATIVE REGULATION OF INSULIN SECRETION | 4 | 0.435195 | 0.0292257 | 1.534235078 |
| GO POSITIVE REGULATION OF SIGNALING RECEPTOR ACTIVITY | 4 | 0.435195 | 0.0292257 | 1.534235078 |
| GO REGULATION OF PROTEIN LOCALIZATION TO CELL SURFACE | 4 | 0.435195 | 0.0292257 | 1.534235078 |
| GO RESPONSE TO FOOD | 4 | 0.435195 | 0.0292257 | 1.534235078 |
| GO INTERLEUKIN 6 PRODUCTION | 7 | 1.56899 | 0.0293827 | 1.531908299 |
| GO REGULATION OF PEPTIDYL SERINE PHOSPHORYLATION | 7 | 1.56899 | 0.0293827 | 1.531908299 |
| GO T CELL PROLIFERATION | 8 | 2.03854 | 0.0296236 | 1.528362165 |
| GO NEGATIVE REGULATION OF LOCOMOTION | 11 | 3.61899 | 0.0298494 | 1.525064394 |
| GO NEGATIVE REGULATION OF PROTEIN BINDING | 6 | 1.14525 | 0.0298494 | 1.525064394 |
| GO MULTI ORGANISM REPRODUCTIVE PROCESS | 23 | 11.4181 | 0.0301315 | 1.520979248 |
| GO POSITIVE REGULATION OF TYROSINE PHOSPHORYLATION OF STAT PROTEIN | 5 | 0.767317 | 0.0304466 | 1.516461198 |
| GO REGULATION OF BEHAVIOR | 5 | 0.767317 | 0.0304466 | 1.516461198 |
| GO REGULATION OF CELLULAR RESPONSE TO INSULIN STIMULUS | 5 | 0.767317 | 0.0304466 | 1.516461198 |
| GO SYNAPTIC VESICLE RECYCLING | 5 | 0.767317 | 0.0304466 | 1.516461198 |
| GO NEGATIVE REGULATION OF CELL MOTILITY | 10 | 3.08072 | 0.0305393 | 1.515140922 |
| GO POSITIVE REGULATION OF NEUROTRANSMITTER TRANSPORT | 4 | 0.446647 | 0.0315271 | 1.501315976 |
| GO TORC1 SIGNALING | 4 | 0.446647 | 0.0315271 | 1.501315976 |
| GO HORMONE TRANSPORT | 11 | 3.65335 | 0.0317897 | 1.497713571 |
| GO MYOBLAST PROLIFERATION | 3 | 0.194692 | 0.0317982 | 1.497597463 |
| GO POSITIVE REGULATION OF CIRCADIAN RHYTHM | 3 | 0.194692 | 0.0317982 | 1.497597463 |
| GO REGULATION OF CELL GROWTH INVOLVED IN CARDIAC MUSCLE CELL DEVELOPMENT | 3 | 0.194692 | 0.0317982 | 1.497597463 |
| GO REGULATION OF TRANSMISSION OF NERVE IMPULSE | 3 | 0.194692 | 0.0317982 | 1.497597463 |
| GO CELLULAR PROTEIN CONTAINING COMPLEX ASSEMBLY | 24 | 12.2084 | 0.0322137 | 1.49195939 |
| GO FEEDING BEHAVIOR | 6 | 1.16815 | 0.0323768 | 1.489766077 |
| GO REGULATION OF STRESS ACTIVATED PROTEIN KINASE SIGNALING CASCADE | 9 | 2.58826 | 0.0331738 | 1.479204778 |
| GO PROTEIN IMPORT | 8 | 2.08435 | 0.0332743 | 1.477891072 |
| GO NEGATIVE REGULATION OF CELL CYCLE G1 S PHASE TRANSITION | 6 | 1.17961 | 0.0338445 | 1.470511898 |
| GO MACROPHAGE DIFFERENTIATION | 4 | 0.4581 | 0.0340031 | 1.468481487 |
| GO DEVELOPMENTAL GROWTH INVOLVED IN MORPHOGENESIS | 9 | 2.59972 | 0.0340229 | 1.468228671 |
| GO LEARNING | 7 | 1.62625 | 0.0348889 | 1.457312723 |
| GO REGULATION OF CALCIUM ION TRANSMEMBRANE TRANSPORT | 7 | 1.62625 | 0.0348889 | 1.457312723 |
| GO CATION TRANSMEMBRANE TRANSPORT | 20 | 9.45976 | 0.0350719 | 1.455040706 |
| GO SEXUAL REPRODUCTION | 20 | 9.48267 | 0.0360249 | 1.443397216 |
| GO HEAD DEVELOPMENT | 19 | 8.79552 | 0.0360484 | 1.443114007 |
| GO CELLULAR RESPONSE TO ARSENIC CONTAINING SUBSTANCE | 3 | 0.206145 | 0.0360754 | 1.442788845 |
| GO HISTONE H3 DEACETYLATION | 3 | 0.206145 | 0.0360754 | 1.442788845 |
| GO NEGATIVE REGULATION OF VASCULAR SMOOTH MUSCLE CELL PROLIFERATION | 3 | 0.206145 | 0.0360754 | 1.442788845 |
| GO REGULATION OF DEFENSE RESPONSE TO BACTERIUM | 3 | 0.206145 | 0.0360754 | 1.442788845 |

| | | | | |
|---|---|---|---|---|
| GO RESOLUTION OF MEIOTIC RECOMBINATION INTERMEDIATES | 3 | 0.206145 | 0.0360754 | 1.442788845 |
| GO RESPONSE TO UV B | 3 | 0.206145 | 0.0360754 | 1.442788845 |
| GO STRESS GRANULE ASSEMBLY | 3 | 0.206145 | 0.0360754 | 1.442788845 |
| GO TRANSCYTOSIS | 3 | 0.206145 | 0.0360754 | 1.442788845 |
| GO MEIOTIC CELL CYCLE PROCESS | 8 | 2.11871 | 0.036129 | 1.442144059 |
| GO NEGATIVE REGULATION OF LIPID LOCALIZATION | 4 | 0.469552 | 0.036349 | 1.439507533 |
| GO POSITIVE REGULATION OF DENDRITIC SPINE DEVELOPMENT | 4 | 0.469552 | 0.036349 | 1.439507533 |
| GO TRIGLYCERIDE METABOLIC PROCESS | 4 | 0.469552 | 0.036349 | 1.439507533 |
| GO LEUKOCYTE APOPTOTIC PROCESS | 6 | 1.20251 | 0.0363556 | 1.439428684 |
| GO MITOTIC DNA INTEGRITY CHECKPOINT | 6 | 1.20251 | 0.0363556 | 1.439428684 |
| GO POSITIVE REGULATION OF PEPTIDYL SERINE PHOSPHORYLATION | 6 | 1.20251 | 0.0363556 | 1.439428684 |
| GO NEGATIVE REGULATION OF CELL ACTIVATION | 8 | 2.13016 | 0.037072 | 1.430953984 |
| GO RESPONSE TO AXON INJURY | 5 | 0.813127 | 0.0373823 | 1.427333982 |
| GO FOREBRAIN DEVELOPMENT | 12 | 4.3405 | 0.0380365 | 1.419799452 |
| GO ADIPOSE TISSUE DEVELOPMENT | 4 | 0.481005 | 0.039121 | 1.407590052 |
| GO CELLULAR RESPONSE TO GLUCOSE STARVATION | 4 | 0.481005 | 0.039121 | 1.407590052 |
| GO DNA DEPENDENT DNA REPLICATION MAINTENANCE OF FIDELITY | 4 | 0.481005 | 0.039121 | 1.407590052 |
| GO NUCLEOSIDE TRIPHOSPHATE BIOSYNTHETIC PROCESS | 5 | 0.82458 | 0.0394259 | 1.404218384 |
| GO MYOFIBROBLAST DIFFERENTIATION | 2 | 0.04581 | 0.0401628 | 1.396176017 |
| GO NEGATIVE REGULATION OF METALLOENDOPEPTIDASE ACTIVITY | 2 | 0.04581 | 0.0401628 | 1.396176017 |
| GO POSITIVE REGULATION OF DNA CATABOLIC PROCESS | 2 | 0.04581 | 0.0401628 | 1.396176017 |
| GO REGULATION OF MYOFIBROBLAST DIFFERENTIATION | 2 | 0.04581 | 0.0401628 | 1.396176017 |
| GO REGULATION OF SUPEROXIDE DISMUTASE ACTIVITY | 2 | 0.04581 | 0.0401628 | 1.396176017 |
| GO NOTCH SIGNALING PATHWAY | 8 | 2.16452 | 0.0403526 | 1.394128478 |
| GO DENDRITIC SPINE MAINTENANCE | 3 | 0.217597 | 0.0404048 | 1.393567039 |
| GO NEGATIVE REGULATION OF CHEMOKINE PRODUCTION | 3 | 0.217597 | 0.0404048 | 1.393567039 |
| GO NEGATIVE REGULATION OF OXIDATIVE STRESS INDUCED NEURON DEATH | 3 | 0.217597 | 0.0404048 | 1.393567039 |
| GO NEGATIVE REGULATION OF TISSUE REMODELING | 3 | 0.217597 | 0.0404048 | 1.393567039 |
| GO POSITIVE REGULATION OF MACROPHAGE MIGRATION | 3 | 0.217597 | 0.0404048 | 1.393567039 |
| GO REGULATION OF RESPONSE TO FOOD | 3 | 0.217597 | 0.0404048 | 1.393567039 |
| GO MODIFICATION OF MORPHOLOGY OR PHYSIOLOGY OF OTHER ORGANISM INVOLVED IN SYMBIOTIC INTERACTION | 6 | 1.23687 | 0.0407758 | 1.38959751 |
| GO RAS PROTEIN SIGNAL TRANSDUCTION | 13 | 4.99329 | 0.0407758 | 1.38959751 |
| GO REGULATION OF SYNAPSE ASSEMBLY | 6 | 1.23687 | 0.0407758 | 1.38959751 |
| GO NEGATIVE REGULATION OF CELL CYCLE PHASE TRANSITION | 9 | 2.69134 | 0.0409954 | 1.387264872 |
| GO NEGATIVE REGULATION OF DNA RECOMBINATION | 4 | 0.492457 | 0.0415486 | 1.381443605 |
| GO PHENOL CONTAINING COMPOUND BIOSYNTHETIC PROCESS | 4 | 0.492457 | 0.0415486 | 1.381443605 |
| GO RESPONSE TO ISCHEMIA | 4 | 0.492457 | 0.0415486 | 1.381443605 |
| GO RHYTHMIC BEHAVIOR | 4 | 0.492457 | 0.0415486 | 1.381443605 |
| GO PLACENTA DEVELOPMENT | 7 | 1.69497 | 0.0417729 | 1.379105374 |
| GO REGULATION OF GENE SILENCING | 7 | 1.69497 | 0.0417729 | 1.379105374 |
| GO RESPONSE TO MOLECULE OF BACTERIAL ORIGIN | 11 | 3.82513 | 0.0425149 | 1.371458838 |
| GO POSITIVE REGULATION OF CYTOKINE PRODUCTION | 13 | 5.0391 | 0.0436462 | 1.360053562 |
| GO REGULATION OF PROTEIN LOCALIZATION TO CELL PERIPHERY | 6 | 1.25977 | 0.0441132 | 1.355431437 |
| GO NEGATIVE REGULATION OF RESPONSE TO ENDOPLASMIC RETICULUM STRESS | 4 | 0.50391 | 0.0445932 | 1.350731362 |
| GO NEGATIVE REGULATION OF STRESS ACTIVATED PROTEIN KINASE SIGNALING CASCADE | 4 | 0.50391 | 0.0445932 | 1.350731362 |

| | | | | |
|---|---|---|---|---|
| GO POSITIVE REGULATION OF CARDIAC MUSCLE TISSUE DEVELOPMENT | 4 | 0.50391 | 0.0445932 | 1.350731362 |
| GO CELL MORPHOGENESIS INVOLVED IN DIFFERENTIATION | 18 | 8.32597 | 0.0449725 | 1.34705297 |
| GO DRUG METABOLIC PROCESS | 11 | 3.85949 | 0.0451427 | 1.345412469 |
| GO NEGATIVE REGULATION OF ENDOPLASMIC RETICULUM STRESS INDUCED INTRINSIC APOPTOTIC SIGNALING PATHWAY | 3 | 0.22905 | 0.0451427 | 1.345412469 |
| GO NEGATIVE REGULATION OF NUCLEOCYTOPLASMIC TRANSPORT | 3 | 0.22905 | 0.0451427 | 1.345412469 |
| GO REGULATION OF PRODUCTION OF SMALL RNA INVOLVED IN GENE SILENCING BY RNA | 3 | 0.22905 | 0.0451427 | 1.345412469 |
| GO SMALL GTPASE MEDIATED SIGNAL TRANSDUCTION | 15 | 6.33323 | 0.0451427 | 1.345412469 |
| GO PERIPHERAL NERVOUS SYSTEM DEVELOPMENT | 5 | 0.858937 | 0.0451455 | 1.345385533 |
| GO REGULATION OF IMMUNE EFFECTOR PROCESS | 12 | 4.45502 | 0.0451455 | 1.345385533 |
| GO BLOOD VESSEL ENDOTHELIAL CELL MIGRATION | 6 | 1.28268 | 0.0475513 | 1.322837605 |
| GO NEGATIVE REGULATION OF PEPTIDE HORMONE SECRETION | 4 | 0.515362 | 0.0476426 | 1.322004546 |
| GO REGULATION OF ERYTHROCYTE DIFFERENTIATION | 4 | 0.515362 | 0.0476426 | 1.322004546 |
| GO CELL PART MORPHOGENESIS | 17 | 7.70753 | 0.0481234 | 1.317643697 |
| GO RECEPTOR SIGNALING PATHWAY VIA STAT | 7 | 1.75223 | 0.0491014 | 1.308906125 |
| GO CELLULAR COMPONENT MORPHOGENESIS | 24 | 12.7008 | 0.0498446 | 1.302381885 |
| GO ADULT LOCOMOTORY BEHAVIOR | 5 | 0.881842 | 0.0499658 | 1.301327155 |
